# Population genomic response to geographic gradients by widespread and endemic fishes of the Arabian Peninsula

**DOI:** 10.1002/ece3.6199

**Published:** 2020-04-12

**Authors:** Joseph D. DiBattista, Pablo Saenz‐Agudelo, Marek J. Piatek, Edgar Fernando Cagua, Brian W. Bowen, John Howard Choat, Luiz A. Rocha, Michelle R. Gaither, Jean‐Paul A. Hobbs, Tane H. Sinclair‐Taylor, Jennifer H. McIlwain, Mark A. Priest, Camrin D. Braun, Nigel E. Hussey, Steven T. Kessel, Michael L. Berumen

**Affiliations:** ^1^ Division of Biological and Environmental Science and Engineering Red Sea Research Center King Abdullah University of Science and Technology Thuwal Saudi Arabia; ^2^ School of Molecular and Life Sciences Curtin University Perth WA Australia; ^3^ Australian Museum Research Institute Australian Museum Sydney NSW Australia; ^4^ Instituto de Ciencias Ambientales y Evolutivas Universidad Austral de Chile Valdivia Chile; ^5^ Computational Bioscience Research Center King Abdullah University of Science and Technology Thuwal Saudi Arabia; ^6^ Biosciences Division Oak Ridge National Laboratory Oak Ridge TN USA; ^7^ Centre for Integrative Ecology School of Biological Sciences University of Canterbury Christchurch New Zealand; ^8^ Hawai‘i Institute of Marine Biology Kāne‘ohe HI USA; ^9^ School of Marine and Tropical Biology James Cook University Townsville Qld Australia; ^10^ Section of Ichthyology California Academy of Sciences San Francisco CA USA; ^11^ Genomics and Bioinformatics Cluster Department of Biology University of Central Florida Orlando FL USA; ^12^ School of Biological Sciences University of Queensland Brisbane Qld Australia; ^13^ Australian Institute of Marine Science Townsville Qld Australia; ^14^ Marine Spatial Ecology Lab School of Biological Sciences and ARC Centre of Excellence for Coral Reef Studies University of Queensland St. Lucia Qld Australia; ^15^ School of Aquatic and Fishery Sciences University of Washington Seattle WA USA; ^16^ Biological Sciences University of Windsor Windsor ON Canada; ^17^ Daniel P. Haerther Center for Conservation and Research John G. Shedd Aquarium Chicago IL USA

**Keywords:** butterflyfishes, coral reefs, ddRAD, Indo‐West Pacific, single nucleotide polymorphism, vicariance

## Abstract

Genetic structure within marine species may be driven by local adaptation to their environment, or alternatively by historical processes, such as geographic isolation. The gulfs and seas bordering the Arabian Peninsula offer an ideal setting to examine connectivity patterns in coral reef fishes with respect to environmental gradients and vicariance. The Red Sea is characterized by a unique marine fauna, historical periods of desiccation and isolation, as well as environmental gradients in salinity, temperature, and primary productivity that vary both by latitude and by season. The adjacent Arabian Sea is characterized by a sharper environmental gradient, ranging from extensive coral cover and warm temperatures in the southwest, to sparse coral cover, cooler temperatures, and seasonal upwelling in the northeast. Reef fish, however, are not confined to these seas, with some Red Sea fishes extending varying distances into the northern Arabian Sea, while their pelagic larvae are presumably capable of much greater dispersal. These species must therefore cope with a diversity of conditions that invoke the possibility of steep clines in natural selection. Here, we test for genetic structure in two widespread reef fish species (a butterflyfish and surgeonfish) and eight range‐restricted butterflyfishes across the Red Sea and Arabian Sea using genome‐wide single nucleotide polymorphisms. We performed multiple matrix regression with randomization analyses on genetic distances for all species, as well as reconstructed scenarios for population subdivision in the species with signatures of isolation. We found that (a) widespread species displayed more genetic subdivision than regional endemics and (b) this genetic structure was not correlated with contemporary environmental parameters but instead may reflect historical events. We propose that the endemic species may be adapted to a diversity of local conditions, but the widespread species are instead subject to ecological filtering where different combinations of genotypes persist under divergent ecological regimes.

## Graphical abstract

We test for genetic structure in two widespread species (a butterflyfish and surgeonfish) and eight range‐restricted butterflyfishes across the Red Sea and Arabian Sea using genome‐wide single nucleotide polymorphisms, multiple matrix regression with randomization analyses, and demographic reconstruction. We found that (a) widespread species displayed more genetic subdivision than regional endemics and (b) this genetic structure was not correlated with contemporary environmental parameters but instead may reflect historical events.

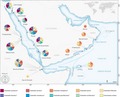

## 1 INTRODUCTION

In the marine environment, coral reef fishes are a model group for understanding processes of speciation because they are well‐characterized and represent the most diverse vertebrate communities on the planet (Nelson, 2006). Reef fishes have broad geographic ranges (typically much greater than terrestrial species; Jones, Caley, & Munday, 2002), a nearly ubiquitous pelagic larval stage with relatively few barriers to dispersal, and occupy a variety of habitats. Understanding how such traits influence the evolution and distributions of reef fishes has long motivated researchers (Bowen et al., 2013; Cowman & Bellwood, 2013). Our understanding of reef fish evolution and biogeography has benefitted greatly from the rapid development of molecular analyses, and new genomic approaches have illuminated the processes driving genetic differentiation in natural populations at a variety of temporal and spatial scales (e.g., Gaither et al., 2015).

Genome‐wide single nucleotide polymorphisms (SNPs), generated by restriction site‐associated DNA sequencing (RAD‐seq), have proven valuable for studying divergence driven by natural selection in model fishes adapted to freshwater (cichlids, Wagner et al., 2013) and euryhaline environments (threespine stickleback, Hohenlohe et al., 2010), and a number of nonmodel reef fishes (Beltrán, Schizas, Appeldoorn, & Prada, 2017; Bernal, Gaither, Simison, & Rocha, 2017; DiBattista, Travers, et al., 2017; Gould & Dunlap, 2017; Harrison et al., 2017; Picq, McMillan, & Puebla, 2016; Puebla, Bermingham, & McMillan, 2014; Stockwell et al., 2016). Using RAD‐seq technology, Gaither et al. (2015) demonstrated that the strongest signals of selection in a widespread surgeonfish (*Acanthurus olivaceus*) were associated with divergent environmental conditions in a peripheral population. Similarly, Saenz‐Agudelo et al. (2015) showed that loci under selection in a range‐restricted clownfish (*Amphiprion bicinctus*) were geographically structured by environmental gradients across the Red Sea, as well as into the Gulf of Aden and Arabian Sea. Testing the generality of these patterns as precursors to speciation requires evaluating co‐distributed taxa that inhabit contrasting environmental or ecological regimes.

The reefs surrounding the Arabian Peninsula present an excellent arena for testing the genomic consequences of environmental transitions. In contrast to the reef systems of the central Indo‐West Pacific, these peripheral reefs occupy one of the most geologically and oceanographically volatile regions in tropical oceans (DiBattista, Choat, et al., 2016; DiBattista, Gaither, et al., 2017; DiBattista, Roberts, et al., 2016; DiBattista et al., 2015; Simpson, Harrison, Claereboudt, & Planes, 2014; Xu, Ruch, & Jónsson, 2015), and are defined by three prominent features: (a) a sharp increase in nutrient availability in the southern Red Sea, (b) the narrow, shallow Strait of Bab Al Mandab that constitutes the only connection between the Red Sea and Indian Ocean, and (c) seasonal upwelling associated with the northern Indian Ocean monsoon. First, the eutrophic region south of ~17°N in the Red Sea may limit larval dispersal of marine fauna, a hypothesis supported by the disjunctive distribution of some reef fish species (Roberts, Shepherd, & Ormond, 1992), as well as genetic differentiation between populations of reef organisms (Nanninga, Saenz‐Agudelo, Manica, & Berumen, 2014; Giles, Saenz‐Agudelo, Hussey, Ravasi, & Berumen, 2015; Saenz‐Agudelo et al., 2015; Reimer et al., 2017; but see Robitzch, Banguera‐Hinestroza, Sawall, Al‐Sofyani, & Voolstra, 2015). Second, water exchange through the Strait of Bab Al Mandab was repeatedly restricted during Pleistocene glacial cycles when sea level lowered as much as 140 m (Braithwaite, 1987; Rohling et al., 1998). Third, the Indian Ocean monsoon causes profound seasonal changes in ocean temperature, salinity, and productivity (Smeed, 2004; Sofianos, Johns, & Murray, 2002). At the western extreme of the Arabian Sea, the Gulf of Aden and waters of Djibouti have a high and relatively stable temperature regime with extensive limestone reefs and high coral cover (Wilkinson, 2008). At the eastern extreme, the southern coastline of Oman is subject to a “pseudo‐high‐latitude effect,” where seasonally cool sea surface temperatures and monsoonal upwelling events result in rocky reefs with sparse coral cover but dense algal cover (Barber et al., 1995; Savidge, Lennon, & Matthews, 1990; Sheppard, Price, & Roberts, 1992). These substantial changes in reef habitat occur over less than 2,000 km, well within the capacity for larval dispersal and gene flow of most reef fishes (Keith, Herbert, Norton, Hawkins, & Newton, 2011; Keith, Woolsey, Madin, Byrne, & Baird, 2015; Lessios & Robertson, 2006).

The habitat discontinuities, local environmental fluctuations, and vicariance of the coastal seas of the Arabian Peninsula provide an opportunity to investigate the relative importance of dispersal, selection, and historical processes in defining intraspecific or even interspecific genetic architecture. Evidence of selection and local adaptation can be detected with correlations between environmental variables and allele frequencies (Coyne & Orr, 2004; Schluter, 2000). For example, reef fish species distributed across the southern Red Sea and through the Gulf of Aden (including Djibouti) show abrupt changes in demographic features, including life span, over relatively small spatial scales and moderate environmental variation (Taylor, Lindfield, & Choat, 2015; Taylor, Trip, & Choat, 2018). To the east of the Gulf of Aden, the environmentally turbulent Oman upwelling coast is likely to have even greater effects on reef fish demography and assemblage composition (Burt et al., 2011; Priest et al., 2016). Historical processes associated with Pleistocene glacial cycles may have also influenced fish demography and faunal composition (DiBattista, Roberts, et al., 2016). If the contemporary environmental conditions influence genetic architecture, we would then expect strong correlations between those conditions and SNP frequency. Alternatively, if historical conditions were of greater importance, we would predict a weak correlation or no correlation between the two variables.

Reef fishes in the Red Sea, Gulf of Aden, Arabian Sea, and Sea of Oman also provide an opportunity to test for local adaptation across strong environmental gradients in both endemic (i.e., range‐restricted) and widespread reef fishes. We expect that range‐restricted species, which complete their life cycle among these reefs and adjacent oceans, are adapted to local conditions and inherent environmental fluctuations. In contrast, widespread species routinely maintain gene flow with other parts of the Indian Ocean (DiBattista et al., 2013), and larvae arriving from outside the region may be less adapted to these conditions. Although gene flow can prevent local adaptation in widespread species (Lenormand, 2002; Slatkin, 1987), strong differences in ecological conditions between locations can also reduce effective gene flow (Orsini, Vanoverbeke, Swillen, Mergeay, & Meester, 2013). If local adaptation was greater in endemic species compared to widespread species, we would expect the endemics to have less genetic structure than widespread species across a similar geographic range.

Here we use genome‐wide SNPs to investigate genetic structure in reef fishes of the Arabian Peninsula. We adopt a multi‐taxon approach comprising eight regional endemics and two widespread reef fishes, all with larvae capable of long‐distance dispersal. We aim to test (a) for associations between SNPs and contemporary environmental gradients in each species, (b) whether widespread species have greater genetic structure than endemic species across the same geographic region, and (c) determine the influence of vicariant processes on genetic structure by reconstructing scenarios for population subdivision in the species that display signatures of isolation.

## 2 MATERIALS AND METHODS

### 2.1 Sample collection and study

**Figure 1:**
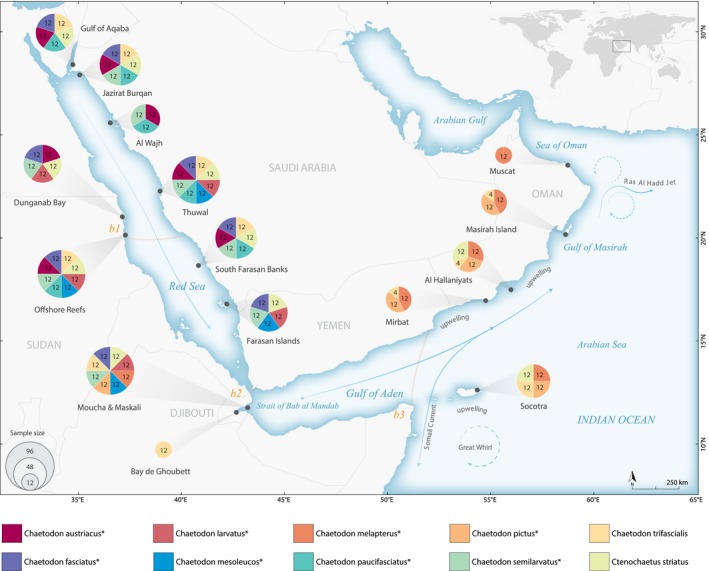
Map indicating collection sites for reef fishes sampled in the Red Sea and Arabian Sea including eight regional endemics (indicated by asterisks) and two widespread species. Colored circles indicate the proportion of samples per species at a site as indicated by the key; circle size is scaled by sample size. Major oceanographic currents and features are represented by arrows. Three putative barriers to larval dispersal are outlined by opaque orange solid lines: b1, 17°N in the Red Sea; b2, Strait of Bab Al Mandab between the Red Sea and Gulf of Aden; and b3, monsoonal upwelling system in the Arabian Sea

We collected tissue samples (fin clip or gill filaments) from individual fish using pole spears at sites between the Gulf of Aqaba in the northern Red Sea (N 28.404°, E 34.738°) and Muscat in the Sea of Oman at the north‐western boundary of the Arabian Sea (N 23.525°, E 58.740°; Figure 1 and Table 1). Nine species are from the butterflyfish family Chaetodontidae and are characterized by a range of geographic distributions (Table 1). We also included a widespread surgeonfish [*Ctenochaetus striatus* (Quoy & Gaimard, 1825)] from the family Acanthuridae. Some of the species were rare or absent at sampling sites, particularly for the regional endemics, which resulted in missing species and data for some locations. While we accept that our sample sizes are modest (Table 1), the number of collections per site and geographic breadth of sampling are far greater than any RAD‐seq population genomics study of reef fishes to date. Tissues were preserved in a saturated salt‐DMSO solution or 95% ethanol and subsequently stored at −20°C.

**Table 1:** STACKS results and genetic diversity metrics for range‐restricted endemics and widespread reef fish sampled in the Red Sea to Arabian Sea (also see Figure 1)

Species	Sample^a^ (*N*)	Number of populations (Geographic range of sampling)	Species^b^ distribution	Number of reads used	Number of polymorphic loci passing filter	*H*_O_	*H*_E_	*F*_IS_
*Chaetodon austriacus* (exquisite butterflyfish)	78	7 (Gulf of Aqaba to South Farasan Banks, Saudi Arabia)	Northern to central Red Sea	87,565,224	10,711	0.0021 (0.2270)	0.0023 (0.2423)	0.0007 (0.0786)
*Chaetodon fasciatus* (Red Sea racoon butterflyfish)	89	8 (Gulf of Aqaba to Djibouti)	Northern Red Sea to Gulf of Aden	77,353,808	2,650	0.0018 (0.2507)	0.0018 (0.2553)	0.0004 (0.0525)
*Chaetodon larvatus* (hooded butterflyfish)	54	5 (Thuwal to Moucha & Maskali, Djibouti)	Southern Red Sea to Gulf of Aden	77,207,403	12,393	0.0015 (0.2456)	0.0016 (0.2577)	0.0005 (0.0735)
*Chaetodon melapterus* (Arabian butterflyfish)	69	6 (Djibouti to Muscat, Oman)	Southern Red Sea to Arabian Gulf	88,380,960	4,384	0.0019 (0.2260)	0.0021 (0.2415)	0.0007 (0.0849)
*Chaetodon mesoleucos* (white‐face butterflyfish)	40	4 (Thuwal to Djibouti)	Southern Red Sea to Gulf of Aden	49,020,448	11,151	0.0013 (0.2687)	0.0014 (0.2764)	0.0003 (0.0659)
*Chaetodon paucifasciatus* (Eritrean butterflyfish)	71	6 (Gulf of Aqaba to South Farasan Banks, Saudi Arabia)	Northern to central Red Sea	89,837,377	13,539	0.0023 (0.2038)	0.0025 (0.2239)	0.0011 (0.0994)
*Chaetodon pictus* (horseshoe butterflyfish)	57	5 (Djibouti to Masirah Island, Oman)	Southern Red Sea to Arabian Gulf	40,198,908	4,131	0.0022 (0.2253)	0.0024 (0.2486)	0.0010 (0.1088)
*Chaetodon semilarvatus* (bluecheek butterflyfish)	93	8 (Jazirat Burqan to Djibouti)	Northern Red Sea to Gulf of Aden	80,036,042	2,053	0.0012 (0.2753)	0.0012 (0.2764)	0.0002 (0.0426)
Regional endemic average				73,700,021	7,627	0.0018 (0.2403)	0.0019 (0.2529)	0.0006 (0.0758)
*Chaetodon trifascialis* (chevron butterflyfish)	102	9 (Gulf of Aqaba to Masirah Island, Oman)	Indo‐Pacific	98,948,301	1,271	0.0010 (0.2489)	0.0010 (0.2494)	0.0002 (0.0420)
*Ctenochaetus striatus* (striated surgeonfish)	101	10 (Gulf of Aqaba to Al Hallaniyats, Oman)	Indo‐Pacific	110,087,471	1,508	0.0019 (0.1863)	0.0021 (0.2072)	0.0010 (0.0945)
Widespread average				104,517,886	1,390	0.0015 (0.2176)	0.0016 (0.2283)	0.0006 (0.1365)

Note

Numbers outside and inside parentheses for genetic diversity metrics are based on all single nucleotide polymorphism (SNP) loci versus only variable SNP loci, respectively.

Abbreviations: *H*_E_, expected heterozygosity; *H*_O_, observed heterozygosity; SNP, single nucleotide polymorphism.

^a^ In most cases, 12 individuals were sampled per population prior to quality filtering, except for *C. trifascialis*, where *N* = 4 were sampled from Mirbat, Al Hallaniyats, and Masirah Island.

^b^ Species distribution is based on a regional database curated over 30 years by R. Myers (see Appendix S2 from DiBattista, Roberts, et al., 2016) but was modified to reflect where species are functionally present versus rare records as waifs.

### 2.2 Ethics statement

This research was undertaken in accordance with the policies and procedures of the King Abdullah University of Science and Technology (KAUST). Permits for sampling in Saudi Arabian waters were obtained from the Saudi Arabian coastguard. No specific permissions were required, as the study did not involve endangered or protected species. We were unable to obtain ethics approval or a waiver because no ethics board or committee for working with animals existed within KAUST at the time of collection.

### 2.3 RAD sequencing

DNA was extracted with NucleoSpin Tissue kits (Macherey‐Nagel Düren). RAD‐seq libraries were prepared following Peterson, Weber, Kay, Fisher, and Hoekstra (2012) using 500 ng of DNA per specimen. Library preparation and Illumina sequencing are detailed in DiBattista, Saenz‐Agudelo, et al. (2017).

Sequences were de‐multiplexed and filtered for quality using the *process_radtags* pipeline in STACKS *vers.* 1.44 (Catchen, Amores, Hohenlohe, Cresko, & Postlethwait, 2011). Raw reads were trimmed from 101 bp to a common length of 81 bp in FASTQ format. Individual reads with Phred scores ≤ 20 (in a 5 bp sliding window) or with ambiguous barcodes were discarded. All loci were assembled separately individuals using the *denovo_map* pipeline in STACKS. Although an annotated butterflyfish genome is available (*Chaetodon austriacus*; DiBattista, Saenz‐Agudelo, et al., 2017), the other species considered in this study are too divergent to enable the recovery of sufficient numbers of SNPs for this comparison, and so we rely on de novo assembly for all species in this case.

For the main analyses presented here, we used a parameter combination tested and optimized as part of DiBattista, Saenz‐Agudelo, et al. (2017) in these same reef fish species: minimum read depth to create a stack (‐m) = 3; number of mismatches allowed between stacks prior to merging (‐M) = 4; maximum number of mismatches when aligning secondary reads to primary stacks (‐N) = 2; maximum number of mismatches allowed between loci when creating a catalog (‐n) = 2. We performed additional data filtering using the “population” component of STACKS retaining only those loci that met the following criteria: (a) minor allele frequency > 0.05, (b) present in at least n−1 population (populations: ‐p), and (c) genotyped in at least 80% of individuals per population (populations: ‐r). We used the “*write_single_snp*” option and produced a.vcf file with the resulting loci to better conform to the assumption of independent loci. The resulting.vcf file was reformatted to other program input files using PGDSPIDER *vers.* 2.0.5.1 (Lischer & Excoffier, 2012). We repeated this process to produce a data set that was comprised of SNPs shared between two of the most closely related species (*C. austriacus* and *C. melapterus*) in order to benchmark patterns of intra‐ versus interspecific genetic variation. Pairwise *F_ST_* values were estimated in STACKS with the “populations” module using the “‐‐fstats” flag option.

### 2.4 Genetic structure analyses

We determined the magnitude of population structure for each of the 10 species by addressing the following two questions: (a) Was there evidence for restricted gene flow based on *F*_ST_ estimates and clustering analyses? and (b) if so, did this restriction conform to one of the following three models: isolation by barrier (IBB; i.e., vicariance), isolation by distance (IBD), or isolation by environment (IBE; i.e., model testing)? IBE refers to scenarios where strong differences in environmental conditions between locations reduce effective gene flow.

Genetic diversity metrics (number of alleles, observed and expected heterozygosity) were estimated using STACKS. Bayesian clustering analyses were performed using STRUCTURE *vers.* 2.3.4 (Pritchard, Stephens, & Donnelly, 2000) without population priors. We used the admixture model with correlated allele frequencies (Falush, Stephens, & Pritchard, 2003). A burn‐in of 200,000 MCMC iterations was used, followed by 300,000 iterations for each run. *K* was set from 1 to the maximum number of sampling sites per species (range: 4–10), and 5 replicate analyses were run for each value of *K*. The number of clusters was inferred by comparing the ln P[D] among different *K* using the ad hoc statistic Δ*K* (Evanno, Regnaut, & Goudet, 2005; also see Table S1 and Appendix S1).

To complement the results from STRUCTURE and graphically summarize the genetic variation among samples within each species, we conducted a principal component analysis (PCA) of the genotype covariance matrix to summarize the genotypic variation across samples. We did this using the “glPca” function of the R package ADEGENET (Jombart & Ahmed, 2011). We also included an analysis, as outlined above, for a combined data set of the closely related *C. austriacus* and *C. melapterus*. Previous work has suggested that the divergence between these two species is recent (Waldrop et al., 2016), and together they are distributed across the range of sampling sites for the widespread species included in this study.

### 2.5 Determinants of genetic differentiation in the Red Sea to Arabian Sea

For species that showed evidence of population genetic structure, we proceeded to evaluate which three models (IBB, IBD, or IBE) best explained pairwise *F*_ST_ as outlined in Saenz‐Agudelo et al. (2015). Under IBD and IBE, we predict that the degree of population differentiation (measured as pairwise *F*_ST_) increases with increasing geographic or environmental distance. As explained in Wang (2013), these models are not mutually exclusive, and so if environmental distance and geographic distance matrices are not correlated, it is possible to compare the relative contribution of each variable via multiple matrix regression with randomisation (MMRR). Under IBB, we predict that genetic variation changes in a discrete manner. Again, IBB is not mutually exclusive from IBD and IBE, but all this information can be visualized via MMRR (e.g., Saenz‐Agudelo et al., 2015).

**Figure 2:**
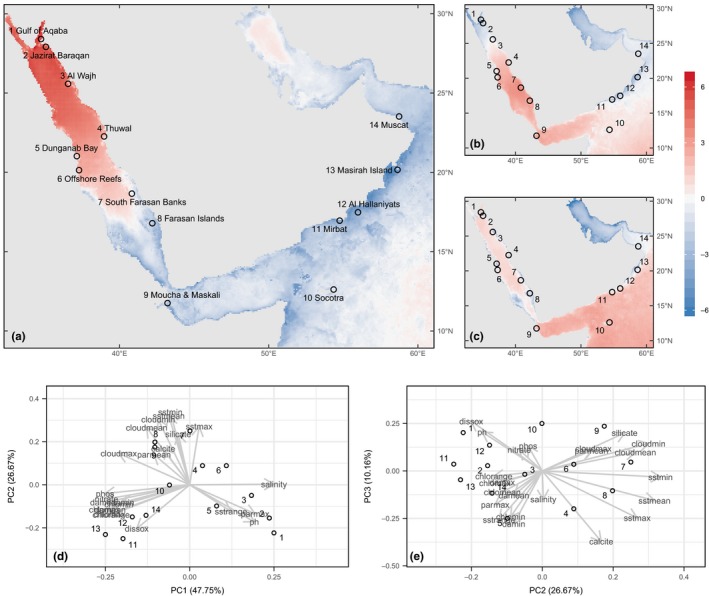
Heat map of environmental data in the Red Sea to Arabian Gulf represented by principal component analysis (PCA) outputs (a) PC1, (b) PC2, and (c) PC3. (d,e) Biplot of the sites and the loading of the environmental drivers underlying the PCA. Collection sites are indicated by numbers

We retrieved gridded environmental information from the Bio‐Oracle database (Tyberghein et al., 2012) to characterize environmental differences among our sampling locations in the Red Sea to Arabian Sea. Specifically, we used aggregated data (predominantly between 2002 and 2009) that described the mean value and variability of environmental variables in a 5‐arc min. grid (~9 km). These variables were related to climate (sea surface temperature, cloud cover, photosynthetically available radiation), chemistry (salinity, pH, dissolved oxygen), nutrients (silicate, nitrate, phosphate, calcite), and productivity (chlorophyll A, diffuse attenuation). To reduce the dimensionality of the data, we performed a PCA based on the correlation of all environmental variables measured at our 14 sampling locations (Figure 2). All variables were log‐transformed and subsequently normalized to have a mean of zero and unit variance.

For each species, we calculated the pairwise *F*_ST_ and the environmental pairwise distance (the distance in PCA space; env) between locations. Because geographic distance between locations might also influence the genetic differentiation within a species, we included pairwise geographic distance (geo) between samples in the analyses. This distance corresponded to the length of the shortest, direct within water path between two given locations. Geographic distances were estimated using the least‐cost distance function (“costDistance”) in the R package gdistance (van Etten, 2017).

Additionally, we explored the influence of three putative barriers to gene flow: (a) at 17°N in the Red Sea, which has been suggested as a potential boundary between ecoregions (see Giles et al., 2015; Nanninga et al., 2014; Roberts et al., 2016), (b) the Strait of Bab Al Mandab, and (c) the monsoonal upwelling system in the Arabian Sea (see Nanninga et al., 2014; Saenz‐Agudelo et al., 2015). All barriers were considered independently and in combination, modeled as a factor with 0 to 3 levels (reflecting the number of barriers between a pair of sites). Sites within the same level were on the same side of the barrier and sites within different levels were on different sides of any given barrier. Seven barrier variables in total were thus included.

We built 67 linear models comprising all biologically plausible combinations of variables (geo, env, and seven barrier variables). Quantitative variables were scaled to a mean of 0 and a variance of 1. For each model, the sample size corrected Akaike information criterion (AICc) was computed as AICc = AIC + 2*K* (*K* + 1)/(*n*−*K*−1), where AIC = −2log‐likelihood + 2*K* (*K* = number of parameters in model; *n* = number of observations). Models were then ranked according to increasing AICc (Anderson, 2008). For each species, the best model was then chosen, and statistical significance of each parameter was estimated via MMRR (Wang, 2013).

### 2.6 Testing for the presence of outlier loci

We ran OutFLANK to test for SNP loci showing departures from neutral expectations, which, in some cases, can indicate whether genetic divergence is linked to adaptive processes (Whitlock & Lotterhos, 2015). Indeed, if different species have homologous loci that depart from neutral expectations, this might suggest that a common adaptive process is driving divergence across species. We ran this analysis for both species that displayed genetic structure (*Ct. striatus* and *C. trifascialis*), as well as for the *C. austriacus* and *C. melapterus* combined data set. OutFLANK (as implemented in R) was used to infer the distribution of *F*_ST_ values and identify putative loci under selection (Whitlock & Lotterhos, 2015). To estimate the null distribution of *F*_ST_ values, we trimmed 5% of the loci from the lower and upper ends of the *F*_ST_ range and included only those loci with heterozygosity higher than 0.1. We used a false discovery rate threshold of 0.05 to calculate *q*‐values when testing for the neutrality of each SNP locus.

### 2.7 Reconstructing scenarios for genetic differentiation in the Red Sea to Arabian Sea

We used a modified version of the diffusion approximation method implemented in ∂a∂I (Gutenkunst, Hernandez, Williamson, & Bustamante, 2009) to explore the joint site‐frequency spectrum (JSFS) of population pairs for species with population partitions identified in the genetic structure analyses. We used the approach outlined by Tine et al. (2014) that includes modifications regarding annealing optimization prior to the Broyden–Fletcher–Goldfarb–Shanno step, which has been shown to improve global parameter convergence. Other modifications include the incorporation of varying migration rates across the genome as described by Tine et al. (2014) and Rougemont et al. (2017). This analysis was performed only for *Ct. striatus*, *C. trifascialis*, as well as the *C. austriacus* and *C. melapterus* combined data set. For these analyses, we grouped samples from the Red Sea and samples from the Indian Ocean together in order to increase the number of individuals used for JSFS estimation. Since PCA analyses of *Ct. striatus* suggested genetic differentiation between Socotra and Oman samples, we additionally ran all models independently using only Socotra samples or only Oman samples as distinct representatives of the Indian Ocean. All scripts and model descriptions are available from (https://github.com/QuentinRougemont/DemographicInference).

Here, we limited our analyses to seven models of divergence including strict isolation (SI), isolation with migration (IM), ancient migration (AM), and secondary contact (SC). For each of IM, AM, and SC, we explored two options: (a) homogenous migration and (b) heterogeneous migration along the genome (2M; as described in Rougemont et al., 2017). To maximize convergence of parameter estimates, each model was run 20 times; the run providing the lowest AIC score was kept and used to compare against other models as well as to estimate model parameters.

## 3 RESULTS

A total of 798,635,942 reads of 101 bp were obtained for 678 sampled individuals from the 10 study species (Table 1); 132 individuals were discarded due to a low number of raw reads recovered (≤250,000) or because the number of missing loci exceeded 50%. The average number of usable reads per sample ranged from 705,244 (for *Chaetodon pictus*) to 1,429,766 (for *Chaetodon larvatus*). Overall, 1,271 to 13,539 polymorphic SNP loci met the quality filtering criteria for each species. Summary statistics including observed heterozygosity, expected heterozygosity, and *F*_IS_ are presented in Table 1.

### 3.1 Genetic structure analyses

**Figure 3:**
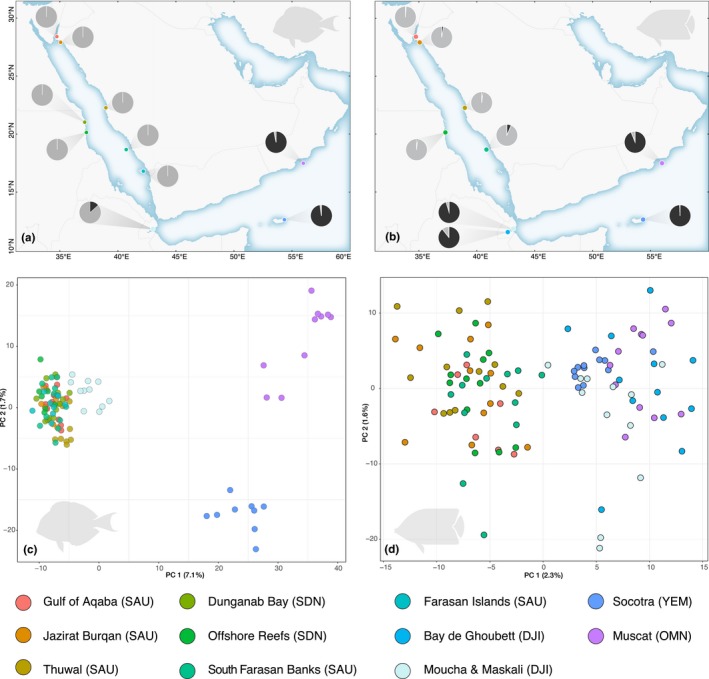
(a,b) Summary of the single nucleotide polymorphism (SNP) admixture estimates from STRUCTURE at each sampling site. The shading in each pie indicate the mean level of admixture per sampling site for *K* = 2. (c,d) Principal component analysis (PCA) scatter plots for RAD‐seq data. Only data sets from the two widespread species *Ctenochaetus striatus* (a,c) and *Chaetodon trifascialis* (b,d) are presented here. For the PCA plots, circles represent individual genotypes and axes show the first two components and the percentage of variance explained in brackets. Three letter abbreviations in parentheses represent the country of sampling

**Figure 4:**
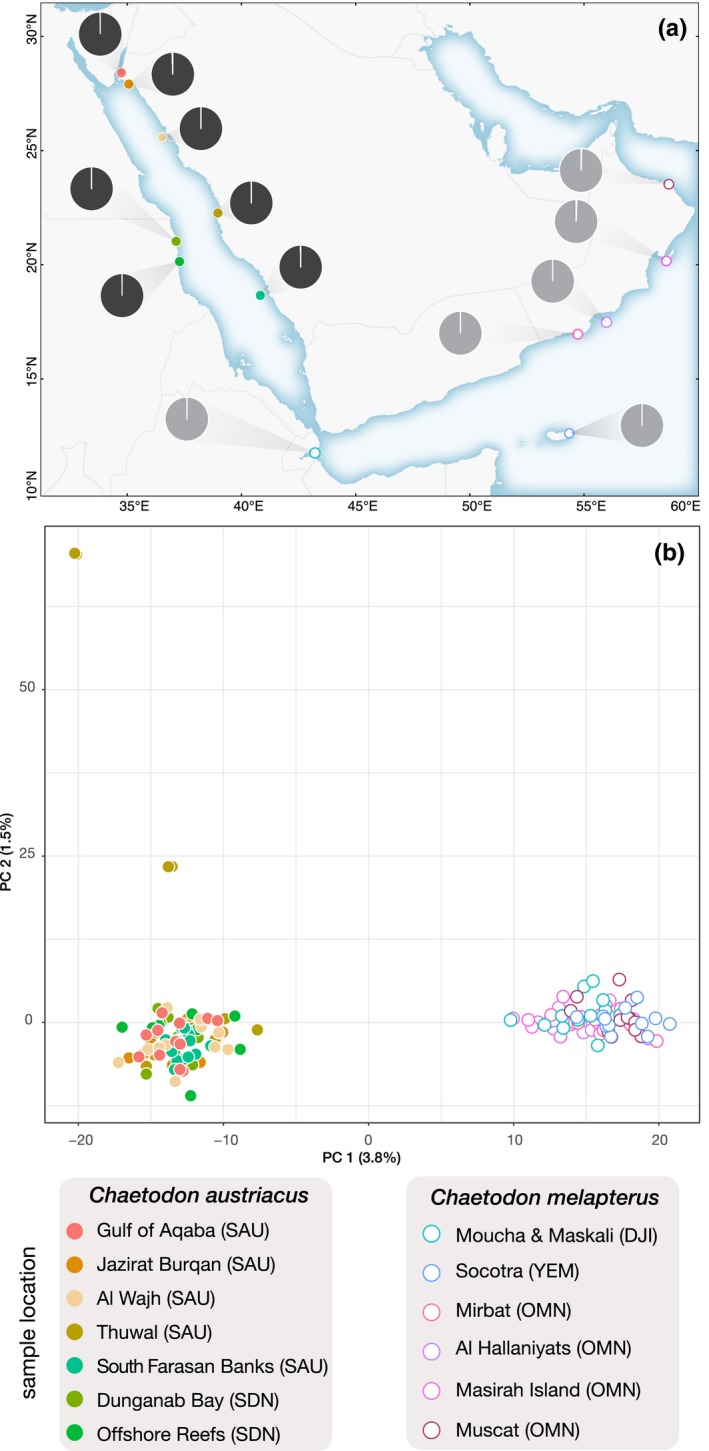
(a) Summary of the single nucleotide polymorphism (SNP) admixture estimates from STRUCTURE at each sampling site. The shading in each pie indicate the mean level of admixture per sampling site for *K* = 2. (b) Principal component analysis (PCA) scatter plots for RAD‐seq data. Only the data set that was comprised of SNPs shared between two closely related species (*Chaetodon austriacus* and *Chaetodon melapterus*) is presented here. For the PCA plots, circles represent individual genotypes and axes show the first two components and the percentage of variance explained in brackets. Three letter abbreviations in parentheses represent the country of sampling

STRUCTURE analyses indicated mean probabilities as being highest at *K* = 1 or ambiguous for all the range‐restricted species, but *K* = 2 (*Ct. striatus*) and *K* = 3 (*C. trifascialis*) for the two widespread species (Table S1). Moreover, PCA was consistent with a scenario of panmixia for all range‐restricted species but not the two widespread species (Figure 3; Appendix S1). Both widespread species demonstrated a putative barrier to dispersal between the Red Sea and the Arabian Sea, with the western end of the Gulf of Aden at Djibouti acting as a transition zone. The data set that included the two closely related species, *C. austriacus* and *C. melapterus*, also indicated that the most likely *K* was 2 (Table S1); two distinct genetic clusters were apparent in the PCA with a similar transition zone of admixture (Figure 4). For reference, STRUCTURE plots for all species at all possible *K* and all PCA plots are provided in Appendix S1

### 3.2 Determinants of genetic differentiation in the Red Sea to Arabian Sea

The first three principal dimensions explained 85% percent of the environmental variability and were used in subsequent analyses (Figure 2). The first component, which explained 48% of the variance, was positively correlated with salinity and nutrient (phosphate, nitrate, chlorophyll) variables. The second component, which explained 27% of the variance, was positively correlated with variables related to sea surface temperature. The third component, which explained 10% of the variance, was positively correlated with dissolved oxygen and pH, and negatively correlated with calcite.

**Figure 5:**
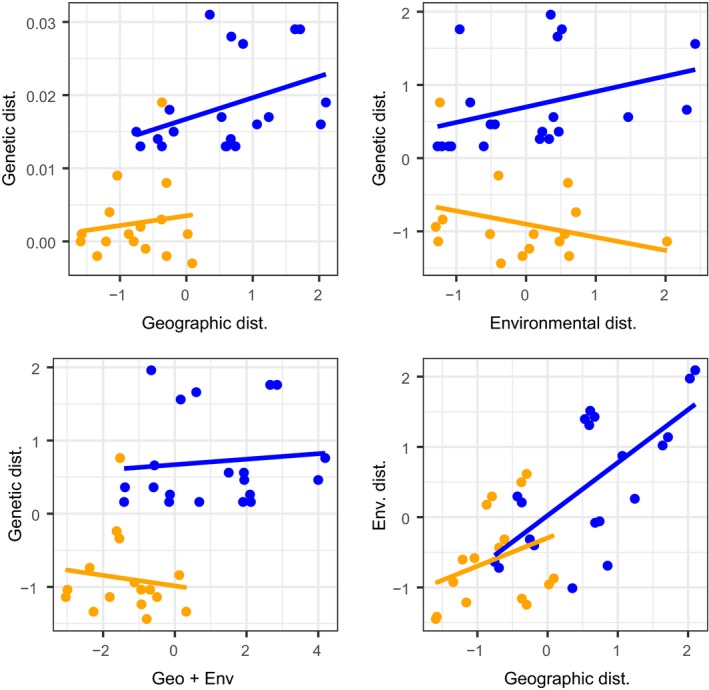
Correlation between pairwise genetic distance (*F*_ST_), geographical distance, and environmental distance for *Chaetodon trifascialis* around the Arabian Peninsula. The top two panels show correlations between genetic and geographic (left) and environmental (right) distances. The bottom two panels show correlations between genetic and combined geographic and environmental distances (left), and the correlation between geographic and environmental distance (right). Blue dots and regression lines correspond to pairwise comparisons among sites on different sides of the Strait of Bab Al Mandab barrier (b2), orange dots and regression lines correspond to pairwise comparisons among sites on the same side of b2

For *C. trifascialis*, a widespread butterflyfish species with apparent genetic structure, comparisons of the 67 models that tested different combinations of the effects of IBB, IBD, and IBE indicated that the four models that best explained genetic differentiation based on pairwise *F*_ST_ (for values see Appendix S2) were those that included the Strait of Bab Al Mandab (b2) as a barrier to gene flow, as well as both geographic and environmental distances (Table S2). Based on these results, the model with the highest probability did not include interactions between variables (model probability, *p* = .240), although metrics of statistical confidence are lowered by the large number of models included for comparison. This best model suggested that: (a) on average, pairwise *F*_ST_ values were lower among sites on the same side of Bab Al Mandab compared to pairwise *F*_ST_ values among sites across Bab Al Mandab (same side of b2: 0.025 ± 0.0011 *SE*, *p* = .010; different side of b2: 0.029 ± 0.0015 *SE*, *p* = .010), 2) pairwise *F*_ST_ values were positively but marginally correlated with geographic distance (slope: 1.541 × 10^–9^ ± 7.932 × 10^–10^ *SE*, *p* = .060) and this slope was the same for comparisons on the same side or across Bab Al Mandab (Figure 5).

**Figure 6:**
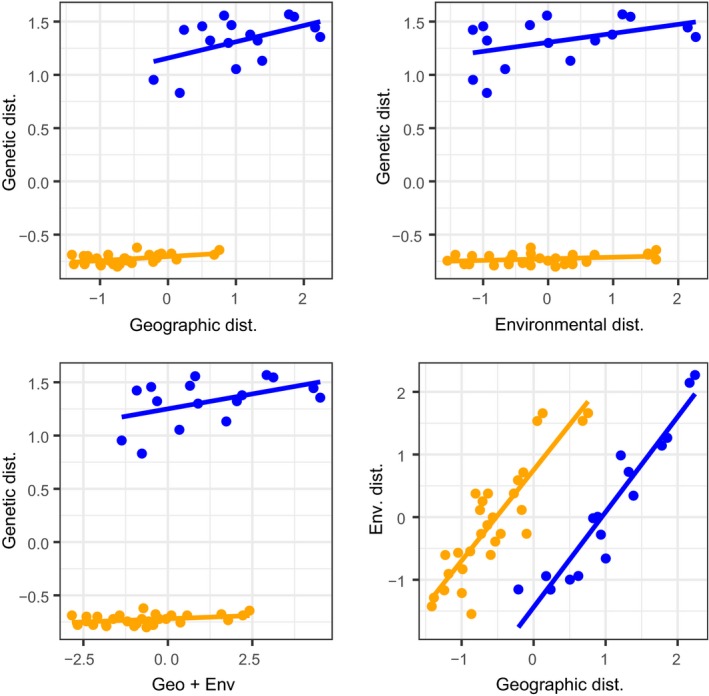
Correlation between pairwise genetic distance (*F*_ST_), geographical distance, and environmental distance for *Ctenochaetus striatus* around the Arabian Peninsula. The top two panels show correlations between genetic and geographic (left) and environmental (right) distances. The bottom two panels show correlations between genetic and combined geographic and environmental distances (left), and the correlation between geographic and environmental distance (right). Blue dots and regression lines correspond to pairwise comparisons among sites on different sides of the monsoonal upwelling system barrier in the Arabian Sea (b3), orange dots and regression lines correspond to pairwise comparisons among sites on the same side of b3

For *Ct. striatus*, the other species demonstrating genetic structure, the four models that best explained genetic differentiation based on pairwise *F*_ST_ were the ones that included the monsoonal upwelling system in the Arabian Sea (b3) as a barrier to gene flow, as well as geographic distance (best two models) or environmental distance (fourth model; Table S3). The best model included an interaction between b3 and geographic distance indicating a difference in slopes between comparisons within and between different sides of the barrier (model *p* = .268). The best model suggested that: (a) on average, pairwise *F*_ST_ values were lower among sites on the same side of b3 compared to pairwise *F*_ST_ values among sites across b3 (same side of b3: 0.032 ± 0.0014 *SE*, *p* < .001; different side of b3: 0.093 ± 0.0028 *SE*, *p* < .001) and (b) pairwise *F*_ST_ values were positively but marginally correlated with geographic distance, but only for comparisons across b3 (same side of b3: 0.00008 ± 0.0018 *SE*, *p* = .965; different side of b3: 0.0048 ± 0.0053 *SE*, *p* = .077; Figure 6). The second‐best model included the same variables but not the interaction term, suggesting the same IBD slope both within and between different sides of b3. That said, this model was 1.5 times less favored than the best model (model *p* = .179). We chose not to run the *C. austriacus* and *C. melapterus* combined data set here because this analysis was entirely focused on within‐species differentiation and not between species differentiation.

### 3.3 Testing for the presence of outlier loci

For *Ct. striatus*, OutFLANK indicated the presence of 73 outlier loci. For *C. trifascialis*, OutFLANK did not detect a single locus under selection. The results from the *C. austriacus* and *C. melapterus* combined data set indicated the presence of 43 outlier loci. A comparison of the consensus sequences of all outlier loci found in the *Ct. striatus* data set versus those from the *C. austriacus* and *C. melapterus* combined data set revealed no shared loci between them.

### 3.4 Reconstructing scenarios for genetic differentiation in the Red Sea to Arabian Sea

**Figure 7:**
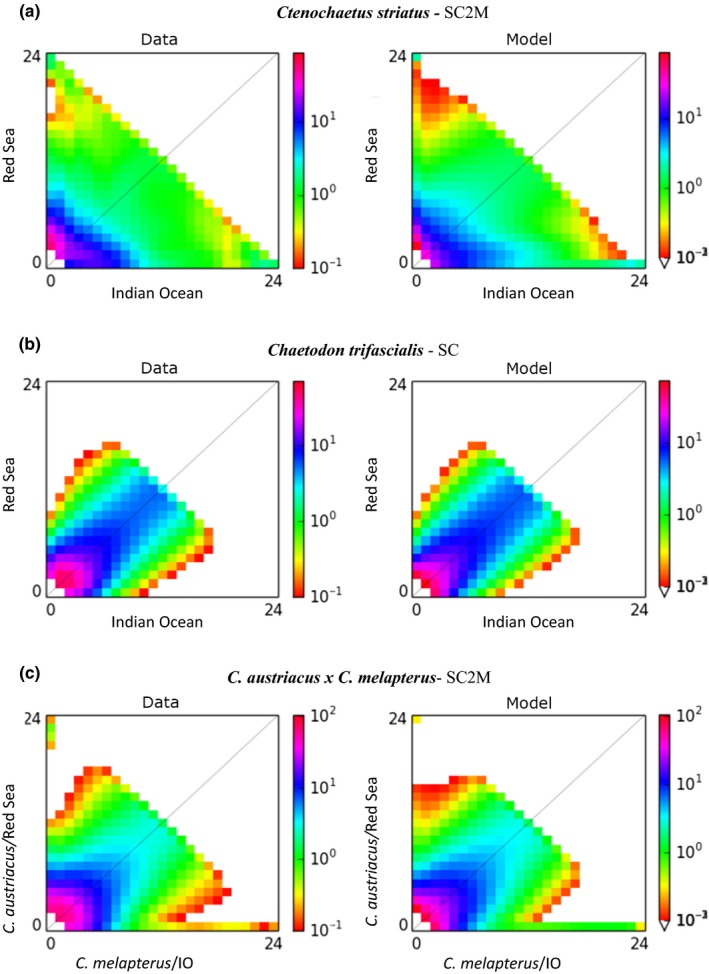
Results of the diffusion approximation models for the (a) *Ctenochaetus striatus*, (b) *Chaetodon trifascialis*, and (c) *Chaetodon austriacus* and *Chaetodon melapterus* data sets. For each data set, the observed “data” and the best fitting “model” are displayed. Shading indicates probability matrix as indicated by the embedded legend. Plots of all alternative models tested, for each data set, are provided as Figures S1–S3

To examine the role of vicariant processes on genetic differentiation, we analyzed the *Ct. striatus* and *C. trifascialis* data sets. We included the *C. austriacus* and *C. melapterus* combined data set here because reconstructing the demographic history within species can share insights about the processes driving genetic divergence between species. These two butterflyfish are recently diverged sister species whose range spans the study region. For all three data sets, the secondary contact (SC) models were consistently better supported by the data (Table 2). For the *Ct. striatus* data set as well as the combined *C. austriacus* and *C. melapterus* data set, this model included heterogeneous migration along the genome (SC2M), whereas for the *C. trifascialis* data set, the SC model had the lowest AIC. Similar results were found for the *Ct. striatus* model that considered Red Sea and Oman samples (where SC2M was the best model), whereas the isolation with migration model (IM2M) was the best model when Socotra samples instead of Oman samples were compared to the Red Sea. We note, however, that the second‐best model for the Socotra to Red Sea comparisons was SC (Table S4). Given that mutation rates are not known for these loci, the values estimated from the JSFS must be interpreted with caution. For all three data sets, effective population size was higher in the Red Sea versus the Indian Ocean, migration rates were higher from the Indian Ocean toward the Red Sea, and the ratio of secondary contact (*T*_sc_) to divergence time (*T*_s_) indicated short periods of introgression for *Ct. striatus* and *C. trifascialis* (3.3% and 4.6% of the total divergence time, respectively), as well as for *C. austriacus* and *C. melapterus* (5.4%). Overall, these results indicate that the evolutionary processes that influence divergence between endemic species (*C. austriacus* and *C. melapterus*) might be similar to those processes influencing divergence within widespread species (i.e., *Ct. striatus* and *C. trifascialis*)*.* The details for each of the six best models tested are shown in Table 2 (also see Figure 7, and Figures S1–S3, and Table S4).

**Table 2:** Comparison of seven alternative demographic models obtained from ∂a∂I for *Ctenochaetus striatus*, *Chaetodon trifascialis*, as well as *Chaetodon austriacus* and *Chaetodon melapterus* data sets using a folded joint frequency spectrum (JSFS)

Model	AIC	log lik	theta	N Red Sea	N Indian Ocean	m12	m21	me12	me21	*T*_s_	*T*_sc_ or *T*_am_	*P*
*Ctenochaetus striatus*												
AM^a^	994.581	−491.291	34.118	14.860	0.239	0.000	6.138			9.828	0.000	
AM2M	934.547	−458.274	34.000	13.794	0.439	0.000	6.033	0.011	0.778	9.961	0.000	0.819
IM	992.578	−491.289	33.636	15.076	0.242	0.000	6.042			9.984		
IM2M	932.132	−458.066	34.319	13.757	0.382	0.000	7.006	0.014	0.837	9.862		0.821
SC	920.964	−454.482	36.853	9.083	0.098	1.119	17.564			9.972	0.074	
**SC2M**	**874.118**	**−428.059**	**38.390**	**8.087**	**0.218**	**0.917**	**9.516**	**0.000**	**1.199**	**4.215**	**0.143**	**0.901**
SI	1,147.322	−570.661	298.115	0.030	0.010					0.002		
*Chaetodon trifascialis*												
AM	724.579	−356.289	357.163	17.442	0.010	0.000	2.991			0.000	0.000	
AM2M	730.536	−356.268	357.235	16.036	0.025	47.853	0.037	0.000	0.000	0.001	0.000	0.785
IM	722.575	−356.287	357.162	18.122	0.010	0.000	0.000			0.000		
IM2M	725.819	−354.910	361.850	0.249	5.479	0.000	68.565	0.000	0.000	0.013		0.891
**SC**	**709.632**	**−348.816**	**86.016**	**2.015**	**1.384**	**3.309**	**19.594**			**9.815**	**0.453**	
SC2M	724.290	−353.145	72.397	3.653	1.856	44.695	0.000	2.744	9.635	8.784	0.584	0.192
SI	718.579	−356.290	357.180	10.464	0.010					0.000		
*C. austriacus* and *C. melapterus*												
AM	897.663	−442.832	50.301	1.331	0.293	0.000	19.766			9.799	0.000	
AM2M	837.919	−409.959	105.878	5.753	0.638	2.673	14.813	0.000	0.398	4.440	0.000	0.948
IM	893.045	−441.523	53.745	12.669	0.294	0.606	19.656			9.344		
IM2M	822.972	−403.486	54.638	12.863	0.191	1.282	44.642	0.000	1.862	9.474		0.948
SC	859.133	−423.567	106.055	2.118	0.413	1.478	14.776			9.877	0.260	
**SC2M**	**814.993**	**−398.497**	**72.889**	**6.564**	**0.884**	**0.000**	**0.958**	**1.444**	**9.975**	**6.132**	**0.332**	**0.053**
SI	1,002.267	−498.134	418.976	0.029	0.010					0.001		

Note

Results of the best run for each model are provided. AIC: Akaike information criterion; log lik: maximum likelihood; theta: 4 *Nrefµ*; N Red Sea and N Indian Ocean: effective population sizes of each population, respectively; m12 and m21: migration rates from the Red Sea to the Indian Ocean and vice versa, respectively; me12 and me21: effective migration rates in the most differentiated regions of the genome (i.e., genomic islands) from the Red Sea to the Indian Ocean and vice versa, respectively; *T*_s_: time of split of the ancestral population into two daughter populations; *T*_sc_: duration of secondary contact episodes (only in SC and SC2M models); *T*_am_: duration of ancestral migration episodes (only in AM and AM2M models); *P*: proportion of the genome exchanged under neutrality. The model with the lowest AIC is indicated in bold.

^a^ Model abbreviations: SI, strict isolation; IM, isolation with migration; AM, ancient migration; SC, secondary contact. For each of IM, AM, and SC, we explored two options: (1) homogenous migration and (2) heterogeneous migration along the genome (2M).

## 4 DISCUSSION

The study region from the northern Red Sea to the northern coast of Oman is characterized by a change from high temperature and extensive coral cover to low temperatures and poor coral development typical of marginal, high‐latitude reefs (Barber et al., 1995; Savidge et al., 1990; Sheppard et al., 1992; Wilkinson, 2008). Across this steep environmental gradient, we found genetic structure in the two widespread species but not the eight regional endemics. Moreover, the genetic structure that we identified in these widespread species was not linked to contemporary environmental conditions but instead mirrored the patterns of separation between two closely related butterflyfish species. Based on this finding and complimentary analyses that reconstructed scenarios of isolation, migration, and secondary contact, the apparent genetic structure appears to be associated with historical processes. Although isolation by adaptation has received both theoretical and empirical support in the terrestrial realm (Orsini et al., 2013), our findings did not support this scenario within this marine environment.

### 4.1 Lack of association between SNPs and contemporary environmental gradients

There was no evidence that the genetic structure found in two study species (*Ct. striatus* and *C. trifascialis*) was linked to contemporary environmental gradients. Interestingly, in both widespread species, the best selected models included the presence of a geographic barrier, which explained most of the variation in pairwise genetic distance. None of the best selected models included environmental distance, and in the lower ranked models that did include it, the slope of the relationship between environmental distance and genetic distance was not significant. This null finding is surprising because two lines of evidence support differential selective regimes across the Red Sea to Arabian Sea. Firstly, age‐growth surveys reveal interpopulation differences in life‐history traits for several coastal fish species (Priest et al., 2016; Robertson, Ackerman, Choat, Posada, & Pitt, 2005; Taylor et al., 2018), including *Ct. striatus* (J. H. Choat, unpublished data). Secondly, there is a considerable difference in the distribution and abundance of *Ct. striatus* and *C. trifascialis* across the study region (Roberts et al., 2016; J. H. McIlwain, unpublished data). The most significant changes in life history and abundance occur across the upwelling region in Oman, with major shifts in temperature, productivity, and water clarity that have persisted since the late Miocene (Zhuang, Pagani, & Zhang, 2017).

It is important to note that we did identify the presence of outlier loci for one (*Ct. striatus*) but not the other (*C. trifascialis*) widespread species*.* The presence of outlier loci along with a lack of IBE correlation for *Ct. striatus* suggests that our simple linear IBE model does not capture the complexity of the processes driving genetic structure in this species. The absence of outlier loci and IBE for *C. trifascialis* instead suggests that demographic rather than selective processes may be driving genetic structure here. Taken together, the observed patterns of genetic structure in these two widespread species indicate that this region is a complex evolutionary arena where both environmental and historical processes may play important roles.

While the influence of the Arabian Sea upwelling zone was not detected in population genetic partitions, this oceanographic feature nonetheless has a profound biogeographic impact. Six of the eight endemic species considered in this study do not occur east of the upwelling region. This concordant range edge for endemic species indicates that these primarily Red Sea inhabitants cannot tolerate upwelling conditions or simply cannot disperse beyond this potential barrier. Thus, the major changes in contemporary environmental conditions may be important for defining the range edge of endemics and genetic structure in widespread species.

### 4.2 Genetic structure of endemic and widespread species

Contemporary environmental gradients within the small range of the endemic species, including barriers b1 and b2 (Figure 1), may not be strong enough to induce genetic structuring in reef fishes. In contrast, both widespread species exhibited genetic differentiation in this study. These widespread species are distributed across the Indo‐West Pacific from the Red Sea to the Hawaiian Archipelago, and *Ct. striatus* had the strongest population genetic partitions. It is likely that these species, like many Indo‐West Pacific reef fishes, arose in the central Indo‐West Pacific and expanded across a vast expanse of ocean to colonize peripheral coral reefs (Briggs, 2005; also see Lawton, Messmer, Pratchett, & Bay, 2011). In contrast, the range‐restricted species persist and evolve under local conditions. Indeed, range‐restricted *C. melapterus* and *C. pictus* (mean range size: 0.21 × 106 km^2^) are thought to be sister species to the wide‐ranging *C. trifasciatus* (different from our study species *C. trifascialis*) and *C. vagabundus* (mean range size: 52.18 × 106 km^2^), respectively, with their speciation driven by peripheral budding in range edge locations (Bowen et al., 2016; Budd & Pandolfi, 2010). Local adaptation would also explain why endemic reef fishes, including the species studied here, tend to achieve much higher abundances than their widespread congeners (Hobbs, Jones, & Munday, 2011; Kane, Kosaki, & Wagner, 2014).

None of the 10 species surveyed displayed genetic structure within the Red Sea. These results contradict previous studies that described the presence of a genetic discontinuity south of 17°N in the Red Sea for an anemonefish (*Amphiprion bicinctus*; Nanninga et al., 2014; Saenz‐Agudelo et al., 2015) and a sponge (*Stylissa carteri*; Giles et al., 2015). One possible explanation for this discrepancy is that both *A. bicinctus* and *S. carteri* are brooders, and therefore have a short pelagic larval duration (PLD). In contrast, all 10 species surveyed in our study are broadcast spawners and so their larvae have relatively long PLDs (Chaetodontidae: PLD ~ 23–56 days; Acanthuridae: PLD ~ 31–91 days; Brothers & Thresher, 1985; Doherty, Planes, & Mather, 1995; Fowler, 1989; Wilson & McCormick, 1999). The link between genetic structure and limited dispersal (Bay, Crozier, & Caley, 2006) means that the 10 study species have dispersal abilities that preclude population genetic structure across the Red Sea region.

In contrast, across a similar geographic distance, many endemic fishes (including butterflyfish and surgeonfish) in the Hawaiian Archipelago demonstrated genetic structure (Toonen et al., 2011). Although the Hawaiian archipelago is also a peripheral region rich in endemic species, it differs in several ways to the Red Sea region. Firstly, it is composed of a series of volcanic islands, compared to the continuous continental coastline of the Red Sea region; the expanses of deep water that separate these islands may promote genetic structure in reef fish populations. Secondly, due to the geomorphology of the Hawaiian Islands, historical events (e.g., sea level change) are less likely to create barriers to gene flow compared to the Red Sea region (e.g., Strait of Bab Al Mandab). Thus, congeneric species occupying different peripheral regions can exhibit contrasting patterns of genetic structure due to differences in geomorphology and historical effects.

### 4.3 Scenarios for population subdivision

One of the most intriguing results in this study was that demographic modeling of both widespread species suggested secondary contact was the most likely scenario of divergence. This model was also favored between the two closely related species, *C. austriacus* and *C. melapterus*, whose combined distributional pattern mirrors the genetic break observed for *C. trifascialis*. The Red Sea and Gulf of Aden have a long history of intermittent isolation at the Strait of Bab Al Mandab, which may partially explain the support for the secondary contact model, at least for *C. trifascialis*. Interestingly, the best model for *C. trifascialis* did not include asymmetric rates of genomic differentiation, which points to neutral rather than selective process shaping differentiation; this is also consistent with our outlier analysis. In contrast, the best models for *Ct. striatus* as well as the combined *C. austriacus* and *C. melapterus* data set indicate heterogeneous genomic divergence, supporting a more complex scenario that includes natural selection, but again it is consistent with our outlier analysis. Thus, traits associated with intrinsic dispersal capacity appear to be of minor importance in determining the geographic distributions of the study species. Other factors, including the geographic configuration of coastal and reef systems, as well as the prevailing oceanographic structure, are likely of primary importance in this context.

## 5 CONCLUSION

Overall, this study found that genetic structure was present in widespread species and absent in endemic species. This genetic structure based on a suite of SNP markers was associated with historical processes and not contemporary environmental conditions or larval dispersal abilities. This novel insight highlights the value of genomic approaches for studying divergence and speciation in nonmodel organisms. This is particularly useful in the marine environment, where research efforts and developments have traditionally lagged behind that of terrestrial and freshwater systems. The marine environment contains some of the most diverse systems in the world (e.g., coral reef ecosystems) and genomic approaches can fast‐track our understanding of the origins and maintenance of this diversity.

## CONFLICT OF INTERESTS

The authors declare no competing interests.

## AUTHOR CONTRIBUTIONS

**Joseph D. DiBattista:** Conceptualization (equal); Data curation (equal); Formal analysis (equal); Investigation (equal); Methodology (equal); Project administration (equal); Resources (equal); Supervision (equal); Validation (equal); Visualization (equal); Writing‐original draft (equal); Writing‐review & editing (equal). **Pablo Saenz‐Agudelo:** Data curation (equal); Formal analysis (equal); Validation (equal); Visualization (equal); Writing‐original draft (equal); Writing‐review & editing (equal). **Marek J. Piatek:** Data curation (equal); Formal analysis (equal); Software (equal); Validation (equal); Visualization (equal); Writing‐original draft (equal); Writing‐review & editing (equal). **E. Fernando Cagua:** Formal analysis (equal); Validation (equal); Visualization (equal); Writing‐original draft (equal); Writing‐review & editing (equal). **Brian W. Bowen:** Investigation (equal); Resources (equal); Writing‐original draft (equal); Writing‐review & editing (equal). **John Howard Choat:** Investigation (equal); Resources (equal); Writing‐original draft (equal); Writing‐review & editing (equal). **Luiz A. Rocha:** Investigation (equal); Resources (equal); Writing‐original draft (equal); Writing‐review & editing (equal). **Michelle R. Gaither:** Investigation (equal); Resources (equal); Writing‐original draft (equal); Writing‐review & editing (equal). **Jean‐Paul A. Hobbs:** Investigation (equal); Resources (equal); Writing‐original draft (equal); Writing‐review & editing (equal). **Tane H. Sinclair‐Taylor:** Investigation (equal); Resources (equal); Visualization (equal); Writing‐original draft (equal); Writing‐review & editing (equal). **Jennifer H. McIlwain:** Investigation (equal); Resources (equal); Writing‐original draft (equal); Writing‐review & editing (equal). **Mark A. Priest:** Investigation (equal); Resources (equal); Writing‐original draft (equal); Writing‐review & editing (equal). **Camrin D. Braun:** Investigation (equal); Resources (equal); Writing‐original draft (equal); Writing‐review & editing (equal). **Nigel E. Hussey:** Investigation (equal); Resources (equal); Writing‐original draft (equal); Writing‐review & editing (equal). **Steven T. Kessel:** Investigation (equal); Resources (equal); Writing‐original draft (equal); Writing‐review & editing (equal). **Michael L. Berumen:** Conceptualization (equal); Funding acquisition (equal); Investigation (equal); Project administration (equal); Resources (equal); Supervision (equal); Writing‐original draft (equal); Writing‐review & editing (equal).

## Supporting information

Figs S1‐S3

Table S1

Table S2

Table S3

Table S4

Appendix S1

Appendix S2

## Data Availability

Raw SNP data and all appendices are available from the Dryad Digital Repository: https://doi.org/10.5061/dryad.rn8pk0p68.
