# Supplementary material for: Population genomic response to geographic gradients by widespread and endemic fishes of the Arabian Peninsula
Source: Ecol Evol. 2020 Apr 12;10(10):4314–30. doi: 10.1002/ece3.6199 (PMC7246217; doi:10.1002/ece3.6199)

**Figure S1.** Results of the best iteration (out of 20 iterations) for each of the seven diffusion approximation models that were evaluated for *Ctenochaetus striatus*. For each model the observed data and the best fitting model are displayed in the top panels. The residuals for fitting the expected spectrum to the data (observed spectrum) are shown in the bottom panels.


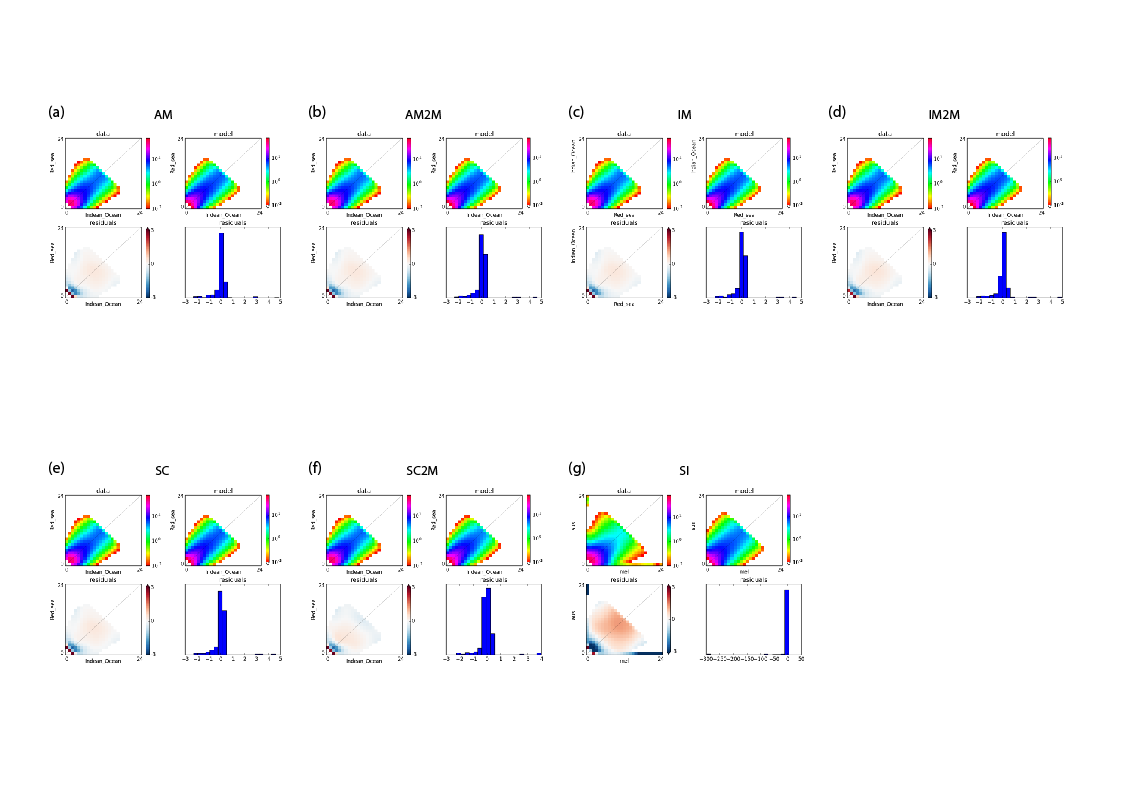


**Figure S2.** Results of the best iteration (out of 20 iterations) for each of the seven diffusion approximation models that were evaluated for *Chaetodon trifascialis*. For each model the observed data and the best fitting model are displayed in the top panels. The residuals for fitting the expected spectrum to the data (observed spectrum) are shown in the bottom panels.


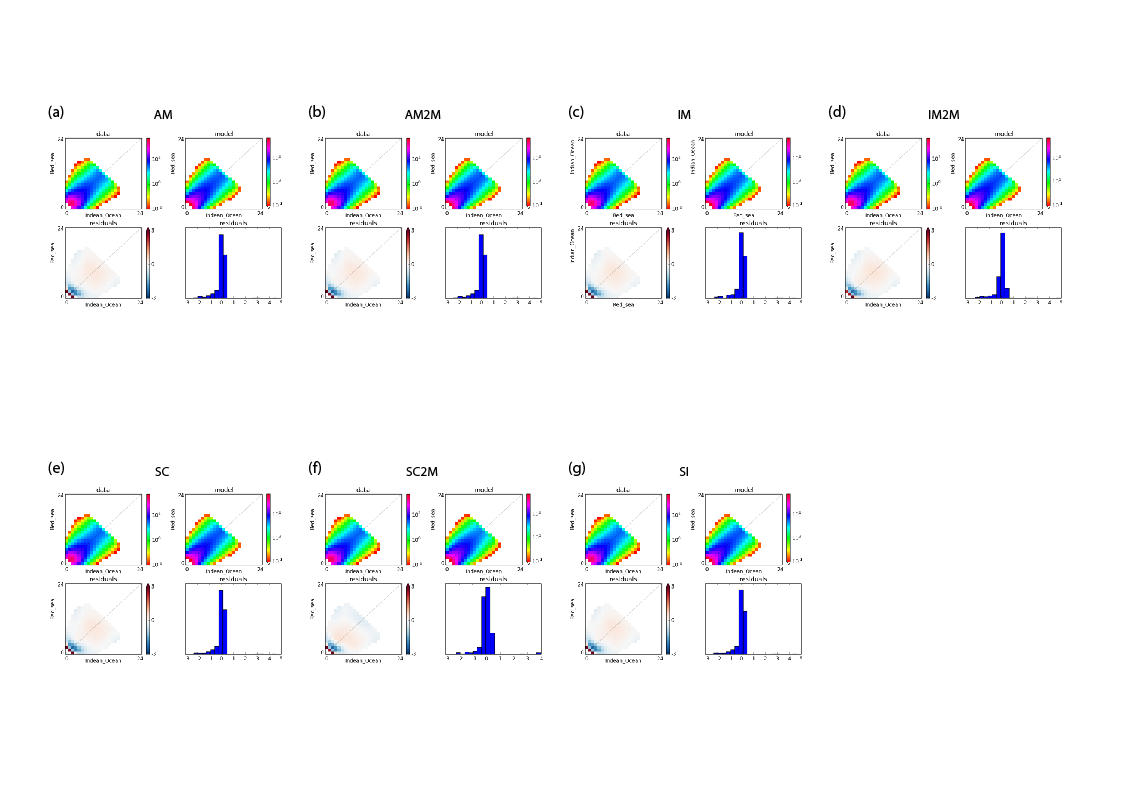


**Figure S3.** Results of the best iteration (out of 20 iterations) for each of the seven diffusion approximation models that were evaluated for the *Chaetodon austriacus* and *Chaetodon melapterus* data set. For each model the observed data and the best fitting model are displayed in the top panels. The residuals for fitting the expected spectrum to the data (observed spectrum) are shown in the bottom panels.


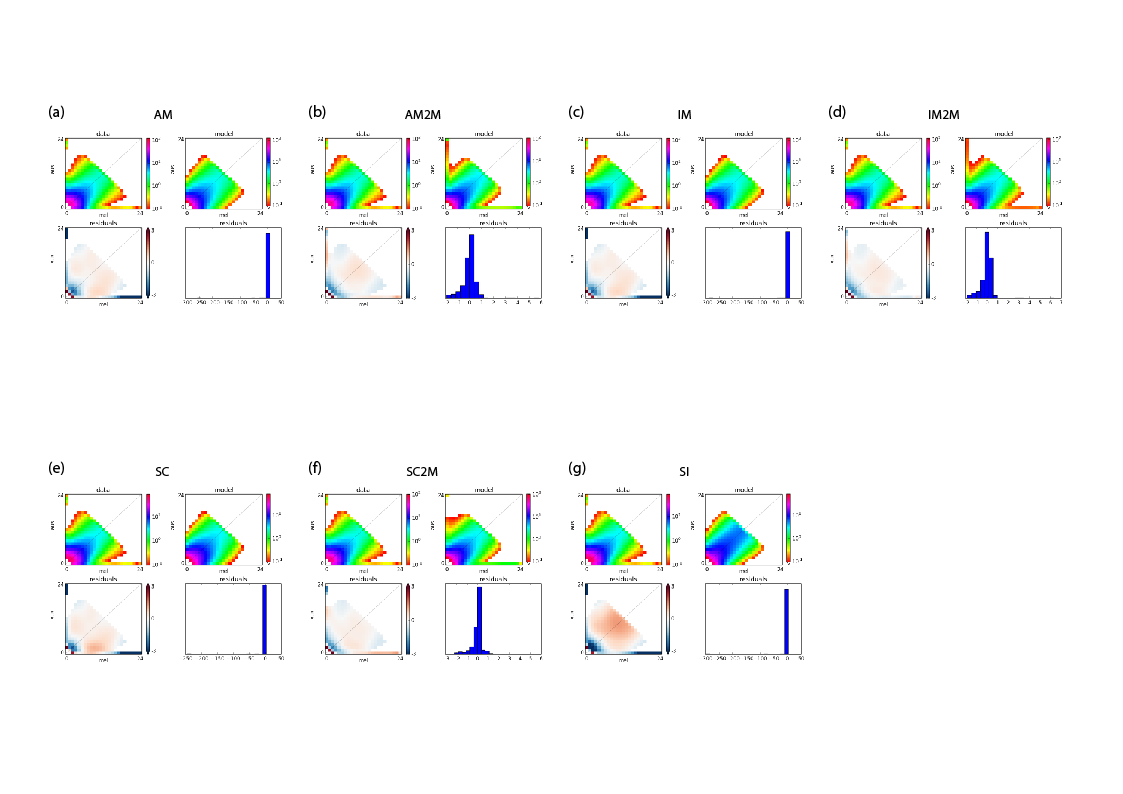

Supplement: Supplementary file 1 — Figs S1‐S3 [file ECE3-10-4314-s001.docx]
