# Supplementary material for: Population genomic response to geographic gradients by widespread and endemic fishes of the Arabian Peninsula
Source: Ecol Evol. 2020 Apr 12;10(10):4314–30. doi: 10.1002/ece3.6199 (PMC7246217; doi:10.1002/ece3.6199)
Supplement: Supplementary file 2 — Table S1 [file ECE3-10-4314-s002.docx]

**Table S1.** Mean Ln probability of data (Ln P[D]) + standard deviation (a) and Evanno's Delta K (b) for range-restricted species, widespread species, and a data set comprised of sister species (*Chaetodon austriacus* and *Chaetodon melapterus*) of reef fish sampled in the Red Sea to Arabian Sea. We additionally provide estimates for the two widespread species at Red Sea sites only.

(a)

|  | *K* = 1 | *K* = 2 | *K* = 3 | *K* = 4 | *K* = 5 | *K* = 6 | *K* = 7 | *K* = 8 | *K* = 9 | *K* = 10 |
| --- | --- | --- | --- | --- | --- | --- | --- | --- | --- | --- |
| Range-restricted species |  |  |  |  |  |  |  |  |  |  |
| *Chaetodon austriacus*  (exquisite butterflyfish) | -638688 + 18 | -638702 + 428 | -640315 + 250 | -643132 + 3510 | -647486 + 5816 | -664304 + 26978 | -670305 + 14343 |  |  |  |
| *Chaetodon fasciatus*  (Red Sea racoon butterflyfish) | -173178 + 3 | -172849 + 9 | -175103 + 1773 | -172691 + 112 | -172784 + 57 | -173414 + 305 | -173766 + 724 | -174277 + 417 |  |  |
| *Chaetodon larvatus*  (hooded butterflyfish) | -540573 + 14 | -545669 + 6626 | -582722 + 23362 | -552815 + 11408 | -552933 + 3643 |  |  |  |  |  |
| *Chaetodon melapterus*  (Arabian butterflyfish) | -212615 + 6 | -211508 + 10 | -210989 + 27 | -210983 + 215 | -218389 + 11679 | -218987 *+* 10331 |  |  |  |  |
| *Chaetodon mesoleucos*  (white-face butterflyfish) | -354603 + 23 | -360649 + 6174 | -362738 + 2241 | -364509 + 2643 |  |  |  |  |  |  |
| *Chaetodon paucifasciatus*  (Eritrean butterflyfish) | -674380 + 25 | -671858 + 1394 | -671432 + 222 | -671232 + 371 | -671318 + 1032 | -682366 + 22659 |  |  |  |  |
| *Chaetodon pictus*  (horseshoe butterflyfish) | -160879 + 5 | -159727 + 17 | -160038 + 57 | -159920 + 108 | -159976 + 170 |  |  |  |  |  |
| *Chaetodon semilarvatus*  (bluecheek butterflyfish) | -153861 + 4 | -153007 + 1 | -153269 + 33 | -153494 + 118 | -153948 + 254 | -154547 + 497 | -154587 + 235 | -155937 + 2554 |  |  |
| Widespread species |  |  |  |  |  |  |  |  |  |  |
| *Chaetodon trifascialis*  (chevron butterflyfish) | -96754 + 1 | -96215 + 8 | -95818 + 4 | -96054 + 91 | -96486 + 649 | -96458 + 39 | -96718 + 167 | -97070 + 583 | -97627 + 612 |  |
| *Chaetodon trifascialis*  (chevron butterflyfish)  Red Sea sites only | -53860 + 1 | -53795 + 4 | -54596 + 471 | -54552 + 837 | -54018 + 78 |  |  |  |  |  |
| *Ctenochaetus striatus*  (striated surgeonfish) | -101413 + 3 | -95325 + 10 | -95348 + 7 | -95015 + 23 | -95316 + 239 | -95507 + 168 | -95795 + 154 | -95717 + 131 | -96072 + 211 | -96621 + 942 |
| *Ctenochaetus striatus*  (striated surgeonfish)  Red Sea sites only | -68510 + 3 | -68372 + 18 | -68202 + 11 | -68255 + 50 | -68311 + 148 | -68539 + 166 | -68531 + 291 |  |  |  |
| Sister species comparison |  |  |  |  |  |  |  |  |  |  |
| *C. austriacus & C. melapterus* | -276412 + 2 | -269144 + 2 | -277950 + 20848 | -268782 + 307 | -292564 + 51055 | -274491 + 10949 | -269366 + 305 | -270731 + 2800 | -270622 + 847 | -275822 + 5053 |

(b)

|  | Delta K = 2 | Delta K = 3 | Delta K = 4 | Delta K = 5 | Delta K = 6 | Delta K = 7 | Delta K = 8 | Delta K = 9 | Delta K = 10 |
| --- | --- | --- | --- | --- | --- | --- | --- | --- | --- |
| Range-restricted species |  |  |  |  |  |  |  |  |  |
| *Chaetodon austriacus*  (exquisite butterflyfish) | 3.73 | 4.82 | 0.44 | 2.14 | 0.40 | 1.45 |  |  |  |
| *Chaetodon fasciatus*  (Red Sea racoon butterflyfish) | 295.24 | 2.63 | 22.37 | 9.39 | 0.91 | 0.22 | 0.21 |  |  |
| *Chaetodon larvatus*  (hooded butterflyfish) | 4.82 | 2.87 | 2.63 | 16.08 |  |  |  |  |  |
| *Chaetodon melapterus*  (Arabian butterflyfish) | 60.02 | 19.22 | 34.40 | 0.58 | 0.57 |  |  |  |  |
| *Chaetodon mesoleucos*  (white-face butterflyfish) | 0.64 | 0.14 | 1.17 |  |  |  |  |  |  |
| *Chaetodon paucifasciatus*  (Eritrean butterflyfish) | 1.50 | 1.01 | 0.77 | 10.62 | 0.55 |  |  |  |  |
| *Chaetodon pictus*  (horseshoe butterflyfish) | 87.94 | 7.48 | 1.60 | 5.23 |  |  |  |  |  |
| *Chaetodon semilarvatus*  (bluecheek butterflyfish) | 832.56 | 1.11 | 1.94 | 0.57 | 1.12 | 5.56 | 0.75 |  |  |
| Widespread species |  |  |  |  |  |  |  |  |  |
| *Chaetodon trifascialis*  (chevron butterflyfish) | 17.46 | 147.37 | 2.15 | 0.71 | 7.36 | 0.55 | 0.35 | 1.19 |  |
| *Chaetodon trifascialis*  (chevron butterflyfish)  Red Sea sites only | 222.75 | 1.80 | 0.59 | 10.17 |  |  |  |  |  |
| *Ctenochaetus striatus*  (striated surgeonfish) | 622.13 | 49.09 | 27.11 | 0.46 | 0.57 | 2.37 | 3.29 | 0.92 | 0.58 |
| *Ctenochaetus striatus*  (striated surgeonfish)  Red Sea sites only | 1.80 | 20.61 | 0.05 | 1.16 | 1.41 | 0.50 |  |  |  |
| Sister species comparison |  |  |  |  |  |  |  |  |  |
| *C. austriacus & C. melapterus* | 7780.31 | 0.86 | 107.23 | 0.82 | 1.18 | 21.48 | 0.54 | 6.27 | 1.12 |
