# Supplementary material for: Population genomic response to geographic gradients by widespread and endemic fishes of the Arabian Peninsula
Source: Ecol Evol. 2020 Apr 12;10(10):4314–30. doi: 10.1002/ece3.6199 (PMC7246217; doi:10.1002/ece3.6199)
Supplement: Supplementary file 3 — Table S2 [file ECE3-10-4314-s003.docx]

**Table S2.** Linear model ranking for the effects of geographic distance, environmental distance, or *a priori* barriers to dispersal for *Chaetodon trifascialis.* Abbreviations are as follows: K, number of parameters in the model; AIC, Akaike Information Criterion; AICc, sample‐size corrected Akaike Information Criterion; RSS, Residual Sum of Squares; r^2^, regression coefficient; Adj.r2, Adjusted regression coefficient; Delta AICc, difference between model´s AICc and best model´s AICc. Model likelihood, exp(-0.5 x Delta AICc), is the model likelihood relative to the best model; Model probability is the Model likelihood divided by the sum of all Model likelihoods; Evidence ratio is the maximum Model likelihood divided by the Model likelihood. Since Model likelihood estimation is based on Delta AICc, it is possible to have models with high r^2^ values but low Model probability values.

| Model | Formula | *K* | AIC | AICc | RSS | r^2^ | Adj.r^2^ | Delta  AICc | Model likelihood | Model probability | Evidence  ratio |
| --- | --- | --- | --- | --- | --- | --- | --- | --- | --- | --- | --- |
| m3 | b2 + geo | 4 | 83.3196 | 84.6099 | 17.079 | 0.512 | 0.4825 | 0 | 1 | 0.2402 | 1 |
| m8 | b2 * geo | 5 | 83.7862 | 85.7862 | 16.3669 | 0.5324 | 0.4885 | 1.1763 | 0.5554 | 0.1334 | 1.8006 |
| m4 | b2 | 3 | 85.2189 | 85.9689 | 19.0329 | 0.4562 | 0.4402 | 1.359 | 0.5069 | 0.1217 | 1.9729 |
| m2 | b2 + env | 4 | 85.3425 | 86.6328 | 18.0662 | 0.4838 | 0.4525 | 2.0229 | 0.3637 | 0.0874 | 2.7496 |
| m1 | b2 + env + geo | 5 | 85.2286 | 87.2286 | 17.036 | 0.5133 | 0.4676 | 2.6187 | 0.27 | 0.0648 | 3.7038 |
| m62 | b1_2_3 | 5 | 85.5387 | 87.5387 | 17.1833 | 0.509 | 0.463 | 2.9288 | 0.2312 | 0.0555 | 4.3249 |
| m12 | b2 * geo + env | 6 | 85.7736 | 88.6701 | 16.3611 | 0.5325 | 0.4722 | 4.0602 | 0.1313 | 0.0315 | 7.6149 |
| m5 | geo | 3 | 88.5127 | 89.2627 | 20.8564 | 0.4041 | 0.3866 | 4.6528 | 0.0976 | 0.0235 | 10.2411 |
| m9 | b2 * env | 5 | 87.304 | 89.304 | 18.0469 | 0.4844 | 0.436 | 4.6941 | 0.0956 | 0.023 | 10.4549 |
| m25 | b1_2 + geo | 5 | 87.3141 | 89.3141 | 18.052 | 0.4842 | 0.4359 | 4.7042 | 0.0952 | 0.0229 | 10.5078 |
| m34 | b3 + geo | 4 | 88.6179 | 89.9082 | 19.7871 | 0.4347 | 0.4004 | 5.2983 | 0.0707 | 0.017 | 14.1422 |
| m61 | b1_2_3 + geo | 6 | 87.0445 | 89.9411 | 16.9491 | 0.5157 | 0.4533 | 5.3312 | 0.0696 | 0.0167 | 14.3766 |
| m60 | b1_2_3 + env | 6 | 87.1538 | 90.0504 | 17.0006 | 0.5143 | 0.4516 | 5.4405 | 0.0659 | 0.0158 | 15.1841 |
| m11 | b2 * env + geo | 6 | 87.2071 | 90.1037 | 17.0258 | 0.5135 | 0.4508 | 5.4938 | 0.0641 | 0.0154 | 15.594 |
| m51 | b2_3 + env | 5 | 88.2824 | 90.2824 | 18.5441 | 0.4702 | 0.4205 | 5.6726 | 0.0586 | 0.0141 | 17.0521 |
| m26 | b1_2 | 4 | 89.1236 | 90.4139 | 20.067 | 0.4267 | 0.3919 | 5.804 | 0.0549 | 0.0132 | 18.2108 |
| m16 | b1 + geo | 4 | 89.5865 | 90.8768 | 20.3266 | 0.4192 | 0.384 | 6.2669 | 0.0436 | 0.0105 | 22.953 |
| m32 | b3 + env + geo | 5 | 88.8816 | 90.8816 | 18.8554 | 0.4613 | 0.4108 | 6.2717 | 0.0435 | 0.0104 | 23.0081 |
| m10 | b2 * env * geo | 7 | 87.174 | 91.174 | 16.0909 | 0.5403 | 0.4636 | 6.5641 | 0.0376 | 0.009 | 26.6302 |
| m6 | env + geo | 4 | 90.2685 | 91.5589 | 20.7154 | 0.4081 | 0.3723 | 6.949 | 0.031 | 0.0074 | 32.281 |
| m18 | b1 * geo | 5 | 89.8115 | 91.8115 | 19.3487 | 0.4472 | 0.3954 | 7.2016 | 0.0273 | 0.0066 | 36.6268 |
| m23 | b1_2 +env + geo | 6 | 89.1993 | 92.0959 | 17.9945 | 0.4859 | 0.4195 | 7.486 | 0.0237 | 0.0057 | 42.2237 |
| m27 | b1_2 * geo | 7 | 88.2731 | 92.2731 | 16.5897 | 0.526 | 0.447 | 7.6632 | 0.0217 | 0.0052 | 46.1355 |
| m50 | b2_3 + env + geo | 6 | 89.5241 | 92.4207 | 18.1576 | 0.4812 | 0.4143 | 7.8108 | 0.0201 | 0.0048 | 49.6689 |
| m36 | b3 * geo | 5 | 90.6052 | 92.6052 | 19.7801 | 0.4349 | 0.3819 | 7.9953 | 0.0184 | 0.0044 | 54.4689 |
| m53 | b2_3 | 4 | 91.3757 | 92.6661 | 21.3625 | 0.3896 | 0.3527 | 8.0562 | 0.0178 | 0.0043 | 56.1534 |
| m24 | b1_2 + env | 5 | 90.7722 | 92.7722 | 19.872 | 0.4322 | 0.379 | 8.1623 | 0.0169 | 0.0041 | 59.2124 |
| m55 | b2_3 * env | 7 | 88.9476 | 92.9476 | 16.9035 | 0.517 | 0.4366 | 8.3377 | 0.0155 | 0.0037 | 64.6413 |
| m59 | b1_2_3 + env + geo | 7 | 88.9787 | 92.9787 | 16.9181 | 0.5166 | 0.4361 | 8.3688 | 0.0152 | 0.0037 | 65.6557 |
| m52 | b2_3 + geo | 5 | 91.2211 | 93.2211 | 20.1214 | 0.4251 | 0.3712 | 8.6113 | 0.0135 | 0.0032 | 74.1157 |
| m14 | b1 + env + geo | 5 | 91.5789 | 93.5789 | 20.3224 | 0.4194 | 0.3649 | 8.969 | 0.0113 | 0.0027 | 88.6341 |
| m39 | b3 * env + geo | 6 | 90.7676 | 93.6641 | 18.7957 | 0.463 | 0.3937 | 9.0542 | 0.0108 | 0.0026 | 92.4911 |
| m40 | b3 * geo + env | 6 | 90.8805 | 93.777 | 18.8548 | 0.4613 | 0.3918 | 9.1672 | 0.0102 | 0.0025 | 97.8638 |
| m43 | b1_3 + geo | 5 | 92.0995 | 94.0995 | 20.6184 | 0.4109 | 0.3557 | 9.4896 | 0.0087 | 0.0021 | 114.9858 |
| m22 | b1 * geo + env | 6 | 91.6992 | 94.5957 | 19.2885 | 0.4489 | 0.3778 | 9.9858 | 0.0068 | 0.0016 | 147.365 |
| m31 | b1_2 * geo + env | 8 | 89.9233 | 95.2567 | 16.4293 | 0.5306 | 0.4335 | 10.6468 | 0.0049 | 0.0012 | 205.0758 |
| m38 | b3 + env + geo + b3 * env + b3 * geo | 7 | 91.7082 | 95.7082 | 18.2507 | 0.4786 | 0.3916 | 11.0983 | 0.0039 | 9.00E-04 | 257.0202 |
| m33 | b3 + env | 4 | 94.7124 | 96.0027 | 23.4371 | 0.3304 | 0.2898 | 11.3928 | 0.0034 | 8.00E-04 | 297.7934 |
| m42 | b1_3 + env | 5 | 94.0962 | 96.0962 | 21.7942 | 0.3773 | 0.3189 | 11.4863 | 0.0032 | 8.00E-04 | 312.0394 |
| m57 | b2_3 * env + geo | 8 | 90.8323 | 96.1656 | 16.8494 | 0.5186 | 0.419 | 11.5557 | 0.0031 | 7.00E-04 | 323.0637 |
| m21 | b1 * env + geo | 6 | 93.3829 | 96.2795 | 20.212 | 0.4225 | 0.348 | 11.6696 | 0.0029 | 7.00E-04 | 341.9945 |
| m41 | b1_3 +env + geo | 6 | 93.812 | 96.7085 | 20.4544 | 0.4156 | 0.3402 | 12.0986 | 0.0024 | 6.00E-04 | 423.8197 |
| m7 | env | 3 | 96.4598 | 97.2098 | 26.0083 | 0.2569 | 0.2351 | 12.5999 | 0.0018 | 4.00E-04 | 544.5481 |
| m44 | b1_3 | 4 | 95.9908 | 97.2812 | 24.2843 | 0.3062 | 0.2641 | 12.6713 | 0.0018 | 4.00E-04 | 564.3252 |
| m20 | b1 + env + geo + b1 * env + b1 * geo | 7 | 93.6881 | 97.6881 | 19.2825 | 0.4491 | 0.3572 | 13.0782 | 0.0014 | 3.00E-04 | 691.6662 |
| m30 | b1_2 * env + geo | 8 | 92.4191 | 97.7524 | 17.6087 | 0.4969 | 0.3928 | 13.1425 | 0.0014 | 3.00E-04 | 714.2742 |
| m28 | b1_2 * env | 7 | 93.8962 | 97.8962 | 19.3943 | 0.4459 | 0.3535 | 13.2863 | 0.0013 | 3.00E-04 | 767.5197 |
| m58 | b2_3 * geo + env | 8 | 93.2271 | 98.5604 | 18.0084 | 0.4855 | 0.379 | 13.9505 | 9.00E-04 | 2.00E-04 | 1069.848 |
| m15 | b1 + env | 4 | 97.2806 | 98.5709 | 25.1702 | 0.2809 | 0.2373 | 13.9611 | 9.00E-04 | 2.00E-04 | 1075.4836 |
| m37 | b3 * env | 5 | 96.6117 | 98.6117 | 23.3716 | 0.3322 | 0.2696 | 14.0018 | 9.00E-04 | 2.00E-04 | 1097.6005 |
| m54 | b2_3 * geo | 7 | 94.9739 | 98.9739 | 19.9837 | 0.429 | 0.3339 | 14.364 | 8.00E-04 | 2.00E-04 | 1315.5113 |
| m63 | b1_2_3 * geo | 9 | 92.2168 | 99.1399 | 16.5638 | 0.5267 | 0.4084 | 14.53 | 7.00E-04 | 2.00E-04 | 1429.3721 |
| m45 | b1_3 * geo | 7 | 95.203 | 99.203 | 20.1113 | 0.4254 | 0.3296 | 14.5931 | 7.00E-04 | 2.00E-04 | 1475.1751 |
| m64 | b1_2_3 * env | 9 | 92.948 | 99.8711 | 16.9037 | 0.517 | 0.3963 | 15.2612 | 5.00E-04 | 1.00E-04 | 2060.305 |
| m17 | b1 | 3 | 99.2503 | 100.0003 | 28.1044 | 0.197 | 0.1734 | 15.3904 | 5.00E-04 | 1.00E-04 | 2197.7353 |
| m19 | b1 * env | 5 | 98.303 | 100.303 | 24.4958 | 0.3001 | 0.2345 | 15.6931 | 4.00E-04 | 1.00E-04 | 2556.8728 |
| m48 | b1_3 * env + geo | 8 | 95.0377 | 100.371 | 18.9373 | 0.4589 | 0.347 | 15.7611 | 4.00E-04 | 1.00E-04 | 2645.3594 |
| m46 | b1_3 * env | 7 | 96.4486 | 100.4486 | 20.8193 | 0.4052 | 0.306 | 15.8387 | 4.00E-04 | 1.00E-04 | 2749.9431 |
| m29 | b1_2 + env + geo + b1_2 * env + b1_2 * geo | 10 | 92.2361 | 101.0361 | 15.6771 | 0.5521 | 0.4194 | 16.4262 | 3.00E-04 | 1.00E-04 | 3688.997 |
| m49 | b1_3 * geo + env | 8 | 96.5902 | 101.9236 | 19.7718 | 0.4351 | 0.3182 | 17.3137 | 2.00E-04 | 0 | 5749.2731 |
| m56 | b2_3 + env + geo + b2_3 * env + b2_3 * geo | 10 | 94.1709 | 102.9709 | 16.5427 | 0.5274 | 0.3873 | 18.3611 | 1.00E-04 | 0 | 9706.2778 |
| m67 | b1_2_3 * geo + env | 10 | 94.2158 | 103.0158 | 16.5633 | 0.5268 | 0.3865 | 18.4059 | 1.00E-04 | 0 | 9926.1721 |
| m66 | b1_2_3 * env + geo | 10 | 94.7074 | 103.5074 | 16.7911 | 0.5203 | 0.3781 | 18.8975 | 1.00E-04 | 0 | 12692.37 |
| m35 | b3 | 3 | 103.5643 | 104.3143 | 31.6824 | 0.0948 | 0.0682 | 19.7044 | 1.00E-04 | 0 | 18999.789 |
| m13 | intercept | 2 | 105.1494 | 105.5131 | 35 | 0 | 0 | 20.9032 | 0 | 0 | 34599.1067 |
| m47 | b1_3 + env + geo + b1_3 * env + b1_3 * geo | 10 | 98.6047 | 107.4047 | 18.7109 | 0.4654 | 0.307 | 22.7948 | 0 | 0 | 89089.1912 |
| m65 | b1_2_3 + env + geo + b1_2_3 * env + b1_2_3 * geo | 13 | 98.9308 | 115.4763 | 15.9826 | 0.5434 | 0.3341 | 30.8664 | 0 | 0 | 5041442.218 |
