# Supplementary material for: Population genomic response to geographic gradients by widespread and endemic fishes of the Arabian Peninsula
Source: Ecol Evol. 2020 Apr 12;10(10):4314–30. doi: 10.1002/ece3.6199 (PMC7246217; doi:10.1002/ece3.6199)
Supplement: Supplementary file 4 — Table S3 [file ECE3-10-4314-s004.docx]

**Table S3.** Linear model ranking for the effects of geographic distance, environmental distance, or *a priori* barriers to dispersal for *Ctenochaetus striatus*. Abbreviations are as follows: K, number of parameters in the model; AIC, Akaike Information Criterion; AICc, sample‐size corrected Akaike Information Criterion; RSS, Residual Sum of Squares; r^2^, regression coefficient; Adj.r2, Adjusted regression coefficient; Delta AICc, difference between model´s AICc and best model´s AICc. Model likelihood, exp(-0.5 x Delta AICc), is the model likelihood relative to the best model; Model probability is the Model likelihood divided by the sum of all Model likelihoods; Evidence ratio is the maximum Model likelihood divided by the Model likelihood. Since Model likelihood estimation is based on Delta AICc, it is possible to have models with high r^2^ values but low Model probability values.

| Model | Formula | *K* | AIC | AICc | RSS | r^2^ | Adj.r^2^ | Delta  AICc | Model likelihood | Model probability | Evidence  ratio |
| --- | --- | --- | --- | --- | --- | --- | --- | --- | --- | --- | --- |
| m36 | b3 * geo | 5 | -27.3555 | -25.817 | 1.1487 | 0.9739 | 0.972 | 0 | 1 | 0.2683 | 1 |
| m34 | b3 + geo | 4 | -26.0139 | -25.0139 | 1.2373 | 0.9719 | 0.9705 | 0.8031 | 0.6693 | 0.1796 | 1.4942 |
| m35 | b3 | 3 | -25.0037 | -24.4183 | 1.3229 | 0.9699 | 0.9692 | 1.3987 | 0.4969 | 0.1333 | 2.0124 |
| m37 | b3 * env | 5 | -25.5685 | -24.03 | 1.1953 | 0.9728 | 0.9708 | 1.787 | 0.4092 | 0.1098 | 2.4436 |
| m33 | b3 + env | 4 | -24.9493 | -23.9493 | 1.2669 | 0.9712 | 0.9698 | 1.8678 | 0.393 | 0.1055 | 2.5444 |
| m40 | b3 * geo + env | 6 | -25.627 | -23.4165 | 1.1418 | 0.974 | 0.9715 | 2.4005 | 0.3011 | 0.0808 | 3.321 |
| m32 | b3 + env + geo | 5 | -24.1807 | -22.6423 | 1.2327 | 0.972 | 0.9699 | 3.1748 | 0.2045 | 0.0549 | 4.8909 |
| m39 | b3 * env + geo | 6 | -24.4585 | -22.248 | 1.1719 | 0.9734 | 0.9707 | 3.5691 | 0.1679 | 0.045 | 5.9568 |
| m38 | b3 + env + geo + b3 * env + b3 * geo | 7 | -23.9173 | -20.8903 | 1.1345 | 0.9742 | 0.9709 | 4.9267 | 0.0851 | 0.0228 | 11.7441 |
| m56 | b2_3 + env + geo + b2_3 * env + b2_3 * geo | 10 | 16.0962 | 22.5668 | 2.4158 | 0.9451 | 0.9329 | 48.3838 | 0 | 0 | 3.21E+10 |
| m50 | b2_3 + env + geo | 6 | 20.4899 | 22.7005 | 3.1818 | 0.9277 | 0.9205 | 48.5175 | 0 | 0 | 3.43E+10 |
| m57 | b2_3 * env + geo | 8 | 20.1848 | 24.1848 | 2.8915 | 0.9343 | 0.9239 | 50.0018 | 0 | 0 | 7.21E+10 |
| m47 | b1_3 + env + geo + b1_3 * env + b1_3 * geo | 10 | 17.8103 | 24.2808 | 2.5096 | 0.943 | 0.9303 | 50.0979 | 0 | 0 | 7.56E+10 |
| m65 | b1_2_3 + env + geo + b1_2_3 * env + b1_2_3 * geo | 13 | 13.4123 | 25.1542 | 1.9918 | 0.9547 | 0.9396 | 50.9712 | 0 | 0 | 1.17E+11 |
| m58 | b2_3 * geo + env | 8 | 24.311 | 28.311 | 3.1692 | 0.928 | 0.9166 | 54.128 | 0 | 0 | 5.67E+11 |
| m55 | b2_3 * env | 7 | 26.4009 | 29.428 | 3.4707 | 0.9211 | 0.911 | 55.245 | 0 | 0 | 9.92E+11 |
| m53 | b2_3 | 4 | 29.0572 | 30.0572 | 4.2069 | 0.9044 | 0.8998 | 55.8742 | 0 | 0 | 1.36E+12 |
| m51 | b2_3 + env | 5 | 28.9921 | 30.5306 | 4.0182 | 0.9087 | 0.902 | 56.3476 | 0 | 0 | 1.72E+12 |
| m52 | b2_3 + geo | 5 | 31.0556 | 32.5941 | 4.2067 | 0.9044 | 0.8974 | 58.4111 | 0 | 0 | 4.83E+12 |
| m48 | b1_3 * env + geo | 8 | 30.2356 | 34.2356 | 3.6151 | 0.9178 | 0.9049 | 60.0526 | 0 | 0 | 1.10E+13 |
| m10 | b2 * env * geo | 7 | 31.6612 | 34.6882 | 3.9011 | 0.9113 | 0.9 | 60.5052 | 0 | 0 | 1.38E+13 |
| m41 | b1_3 +env + geo | 6 | 33.5167 | 35.7272 | 4.2501 | 0.9034 | 0.8937 | 61.5442 | 0 | 0 | 2.31E+13 |
| m12 | b2 * geo + env | 6 | 33.8163 | 36.0269 | 4.2785 | 0.9028 | 0.893 | 61.8439 | 0 | 0 | 2.69E+13 |
| m1 | b2 + env + geo | 5 | 34.7368 | 36.2753 | 4.5653 | 0.8962 | 0.8887 | 62.0923 | 0 | 0 | 3.04E+13 |
| m54 | b2_3 * geo | 7 | 34.8671 | 37.8941 | 4.1892 | 0.9048 | 0.8926 | 63.7111 | 0 | 0 | 6.83E+13 |
| m11 | b2 * env + geo | 6 | 36.6381 | 38.8487 | 4.5553 | 0.8965 | 0.8861 | 64.6657 | 0 | 0 | 1.10E+14 |
| m66 | b1_2_3 * env + geo | 10 | 32.3847 | 38.8553 | 3.4695 | 0.9211 | 0.9036 | 64.6723 | 0 | 0 | 1.11E+14 |
| m6 | env + geo | 4 | 38.617 | 39.617 | 5.2026 | 0.8818 | 0.8761 | 65.4341 | 0 | 0 | 1.62E+14 |
| m23 | b1_2 +env + geo | 6 | 38.4226 | 40.6331 | 4.7396 | 0.8923 | 0.8815 | 66.4501 | 0 | 0 | 2.69E+14 |
| m49 | b1_3 * geo + env | 8 | 37.3446 | 41.3446 | 4.2338 | 0.9038 | 0.8886 | 67.1616 | 0 | 0 | 3.84E+14 |
| m14 | b1 + env + geo | 5 | 40.6083 | 42.1467 | 5.2016 | 0.8818 | 0.8731 | 67.9637 | 0 | 0 | 5.73E+14 |
| m31 | b1_2 * geo + env | 8 | 39.5045 | 43.5045 | 4.442 | 0.899 | 0.8831 | 69.3215 | 0 | 0 | 1.13E+15 |
| m30 | b1_2 * env + geo | 8 | 40.1374 | 44.1374 | 4.5049 | 0.8976 | 0.8814 | 69.9544 | 0 | 0 | 1.55E+15 |
| m59 | b1_2_3 + env + geo | 10 | 38.0395 | 44.5101 | 3.934 | 0.9106 | 0.8907 | 70.3271 | 0 | 0 | 1.87E+15 |
| m67 | b1_2_3 * geo + env | 10 | 38.0395 | 44.5101 | 3.934 | 0.9106 | 0.8907 | 70.3271 | 0 | 0 | 1.87E+15 |
| m21 | b1 * env + geo | 6 | 42.5945 | 44.805 | 5.2 | 0.8818 | 0.87 | 70.622 | 0 | 0 | 2.16E+15 |
| m22 | b1 * geo + env | 6 | 42.6069 | 44.8174 | 5.2015 | 0.8818 | 0.87 | 70.6345 | 0 | 0 | 2.18E+15 |
| m29 | b1_2 + env + geo + b1_2 * env + b1_2 * geo | 10 | 40.015 | 46.4856 | 4.1106 | 0.9066 | 0.8858 | 72.3027 | 0 | 0 | 5.02E+15 |
| m20 | b1 + env + geo + b1 * env + b1 * geo | 7 | 44.5877 | 47.6147 | 5.1992 | 0.8818 | 0.8667 | 73.4317 | 0 | 0 | 8.82E+15 |
| m46 | b1_3 * env | 7 | 56.0644 | 59.0914 | 6.7097 | 0.8475 | 0.828 | 84.9085 | 0 | 0 | 2.74E+18 |
| m42 | b1_3 + env | 5 | 65.669 | 67.2075 | 9.0782 | 0.7937 | 0.7786 | 93.0245 | 0 | 0 | 1.58E+20 |
| m64 | b1_2_3 * env | 9 | 63.8513 | 68.9941 | 7.2987 | 0.8341 | 0.8027 | 94.8111 | 0 | 0 | 3.87E+20 |
| m60 | b1_2_3 + env | 6 | 69.1103 | 71.3208 | 9.3737 | 0.787 | 0.7657 | 97.1379 | 0 | 0 | 1.24E+21 |
| m43 | b1_3 + geo | 5 | 71.2695 | 72.8079 | 10.2813 | 0.7663 | 0.7492 | 98.6249 | 0 | 0 | 2.61E+21 |
| m44 | b1_3 | 4 | 72.0581 | 73.0581 | 10.9386 | 0.7514 | 0.7396 | 98.8751 | 0 | 0 | 2.95E+21 |
| m45 | b1_3 * geo | 7 | 70.8484 | 73.8754 | 9.3193 | 0.7882 | 0.761 | 99.6924 | 0 | 0 | 4.45E+21 |
| m62 | b1_2_3 | 5 | 74.0705 | 75.609 | 10.9416 | 0.7513 | 0.7331 | 101.426 | 0 | 0 | 1.06E+22 |
| m61 | b1_2_3 + geo | 6 | 73.5548 | 75.7653 | 10.3467 | 0.7648 | 0.7413 | 101.5824 | 0 | 0 | 1.14E+22 |
| m63 | b1_2_3 * geo | 9 | 74.3972 | 79.54 | 9.2263 | 0.7903 | 0.7506 | 105.357 | 0 | 0 | 7.55E+22 |
| m16 | b1 + geo | 4 | 83.7973 | 84.7973 | 14.199 | 0.6773 | 0.6619 | 110.6143 | 0 | 0 | 1.05E+24 |
| m5 | geo | 3 | 84.6961 | 85.2814 | 15.1437 | 0.6558 | 0.6478 | 111.0984 | 0 | 0 | 1.33E+24 |
| m3 | b2 + geo | 4 | 85.8924 | 86.8924 | 14.8757 | 0.6619 | 0.6458 | 112.7095 | 0 | 0 | 2.98E+24 |
| m8 | b2 * geo | 5 | 85.3603 | 86.8988 | 14.0618 | 0.6804 | 0.657 | 112.7158 | 0 | 0 | 2.99E+24 |
| m18 | b1 * geo | 5 | 85.5437 | 87.0822 | 14.1192 | 0.6791 | 0.6556 | 112.8992 | 0 | 0 | 3.28E+24 |
| m25 | b1_2 + geo | 5 | 88.1975 | 89.7359 | 14.9769 | 0.6596 | 0.6347 | 115.5529 | 0 | 0 | 1.24E+25 |
| m27 | b1_2 * geo | 7 | 90.792 | 93.819 | 14.5163 | 0.6701 | 0.6278 | 119.6361 | 0 | 0 | 9.52E+25 |
| m4 | b2 | 3 | 107.8576 | 108.4429 | 25.3375 | 0.4241 | 0.4108 | 134.26 | 0 | 0 | 1.43E+29 |
| m2 | b2 + env | 4 | 109.832 | 110.832 | 25.3231 | 0.4245 | 0.3971 | 136.649 | 0 | 0 | 4.71E+29 |
| m9 | b2 * env | 5 | 110.8139 | 112.3524 | 24.7567 | 0.4373 | 0.3962 | 138.1694 | 0 | 0 | 1.01E+30 |
| m26 | b1_2 | 4 | 116.2604 | 117.2604 | 29.2118 | 0.3361 | 0.3045 | 143.0774 | 0 | 0 | 1.17E+31 |
| m24 | b1_2 + env | 5 | 117.2143 | 118.7528 | 28.5406 | 0.3514 | 0.3039 | 144.5698 | 0 | 0 | 2.47E+31 |
| m28 | b1_2 * env | 7 | 118.8828 | 121.9099 | 27.0995 | 0.3841 | 0.3051 | 147.7269 | 0 | 0 | 1.20E+32 |
| m17 | b1 | 3 | 126.4823 | 127.0677 | 38.3276 | 0.1289 | 0.1087 | 152.8847 | 0 | 0 | 1.58E+33 |
| m15 | b1 + env | 4 | 128.3512 | 129.3512 | 38.2161 | 0.1315 | 0.0901 | 155.1682 | 0 | 0 | 4.95E+33 |
| m19 | b1 * env | 5 | 128.7171 | 130.2556 | 36.8532 | 0.1624 | 0.1011 | 156.0726 | 0 | 0 | 7.78E+33 |
| m13 | intercept | 2 | 130.6932 | 130.9789 | 44 | 0 | 0 | 156.7959 | 0 | 0 | 1.12E+34 |
| m7 | env | 3 | 131.5046 | 132.09 | 42.853 | 0.0261 | 0.0034 | 157.907 | 0 | 0 | 1.95E+34 |
