# Supplementary material for: Population genomic response to geographic gradients by widespread and endemic fishes of the Arabian Peninsula
Source: Ecol Evol. 2020 Apr 12;10(10):4314–30. doi: 10.1002/ece3.6199 (PMC7246217; doi:10.1002/ece3.6199)
Supplement: Supplementary file 5 — Table S4 [file ECE3-10-4314-s005.docx]

**Table S4.** Comparison of seven alternative demographic models obtained from ∂a∂I for *Ctenochaetus striatus* data sets that include Oman or Socotra only as Indian Ocean sites using a folded joint frequency spectrum (JSFS). Results of the best run for each model are provided. AIC: Akaike information criterion; log lik: maximum likelihood; theta: 4 *Nrefµ*; N Red Sea and Indian Ocean: effective population sizes of each population, respectively; m12 and m21: migration rates from the Red Sea to the Indian Ocean and vice versa, respectively; me12 and me21: effective migration rates in the most differentiated regions of the genome (i.e. genomic islands) from the Red Sea to the Indian Ocean and vice versa, respectively; T_s_ : time of split of the ancestral population into two daughter populations; T_sc_: duration of secondary contact episodes (only in SC and SC2M models); T_am_: duration of ancestral migration episodes (only in AM and AM2M models); P: proportion of the genome exchanged under neutrality. The model with the lowest AIC is indicated in bold.

| **Model** | **AIC** | **log lik** | **theta** | ***N Red Sea*** | ***Indian Ocean*** | **m12** | **m21** | **me12** | **me21** | **T_s_** | **T_sc_ or T_am_** | **P** |
| --- | --- | --- | --- | --- | --- | --- | --- | --- | --- | --- | --- | --- |
| *Ctenochaetus striatus Red Sea vs Oman* | | | | | | | | | | | | |
| AM^a^ | 623.928 | -322.915 | 64.183 | 13.17 | 0.47 | 0.002 | 2.855 |  |  | 7.674 | 0.000 |  |
| AM2M | 669.872 | -328.015 | 318.774 | 0.599 | 1.408 | 8.836 | 0.000 | 0.395 | 0.000 | 0.772 | 0.000 | 0.789 |
| IM | 621.762 | -306.146 | 51.562 | 16.402 | 0.562 | 0.002 | 2.379 |  |  | 9.816 |  |  |
| IM2M | 591.805 | -290.837 | 149.854 | 16.585 | 0.872 | 32.032 | 0.000 | 0.000 | 1.171 | 9.291 |  | 0.732 |
| SC | 574.743 | -305.731 | 50.119 | 1.610 | 0.505 | 0.240 | 3.247 |  |  | 9.947 | 0.359 |  |
| **SC2M** | **573.425** | **-292.608** | **169.189** | **4.826** | **0.190** | **0.031** | **13.941** | **0.003** | **2.052** | **0.243** | **1.952** | **0.761** |
| SI | 797.564 | -396.820 | 436.528 | 0.156 | 0.075 |  |  |  |  | 0.016 |  |  |
|  | | | | | | | | | | | | |
| *Ctenochaetus striatus Red Sea vs Socotra* | | | | | | | | | | | | |
| AM | 552.489 | -299.305 | 110.878 | 5.261 |  | 0.197 |  | 0.028 | 6.433 |  | 2.471 |  |
| AM2M | 580.7136504 | -283.061 | 238.9178914 | 0.627 | 1.256 | 8.442 | 0.000 | 0.000 | 0.000 | 0.558 | 0.000 | 0.805 |
| IM | 548.744 | -269.916 | 40.866 | 14.153 | 0.542 | 0.002 | 2.343 |  |  | 8.624 |  |  |
| **IM2M** | **525.424** | **-261.324** | **99.4122** | **4.840** | **0.5385** | **0.026** | **0.000** | **0.306** | **2.875** | **2.903** |  | **0.053** |
| SC | 539.540 | -268.860 | 35.794 | 15.165 | 0.074 | 0.000 | 17.749 | 0.000 |  | 9.932 |  |  |
| SC2M | 527.797 | -259.284 | 129.264 | 3.466 | 0.464 | 0.020 | 0.000 | 0.300 | 3.763 | 0.000 | 1.987 | 0.054 |
| SI | 675.617 | -334.916 | 303.083 | 0.238 | 0.111 |  |  |  |  | 0.025 |  |  |

^a^Model abbreviations: strict isolation (SI), isolation with migration (IM), ancient migration (AM), and secondary contact (SC). For each of IM, AM, and SC we explored two options: 1) homogenous migration and 2) heterogeneous migration along the genome (2M).
