# Supplementary figures and images for: Population genomic response to geographic gradients by widespread and endemic fishes of the Arabian Peninsula
Source: Ecol Evol. 2020 Apr 12;10(10):4314–30. doi: 10.1002/ece3.6199 (PMC7246217; doi:10.1002/ece3.6199)

*C. austriacus* & *C. melapterus*

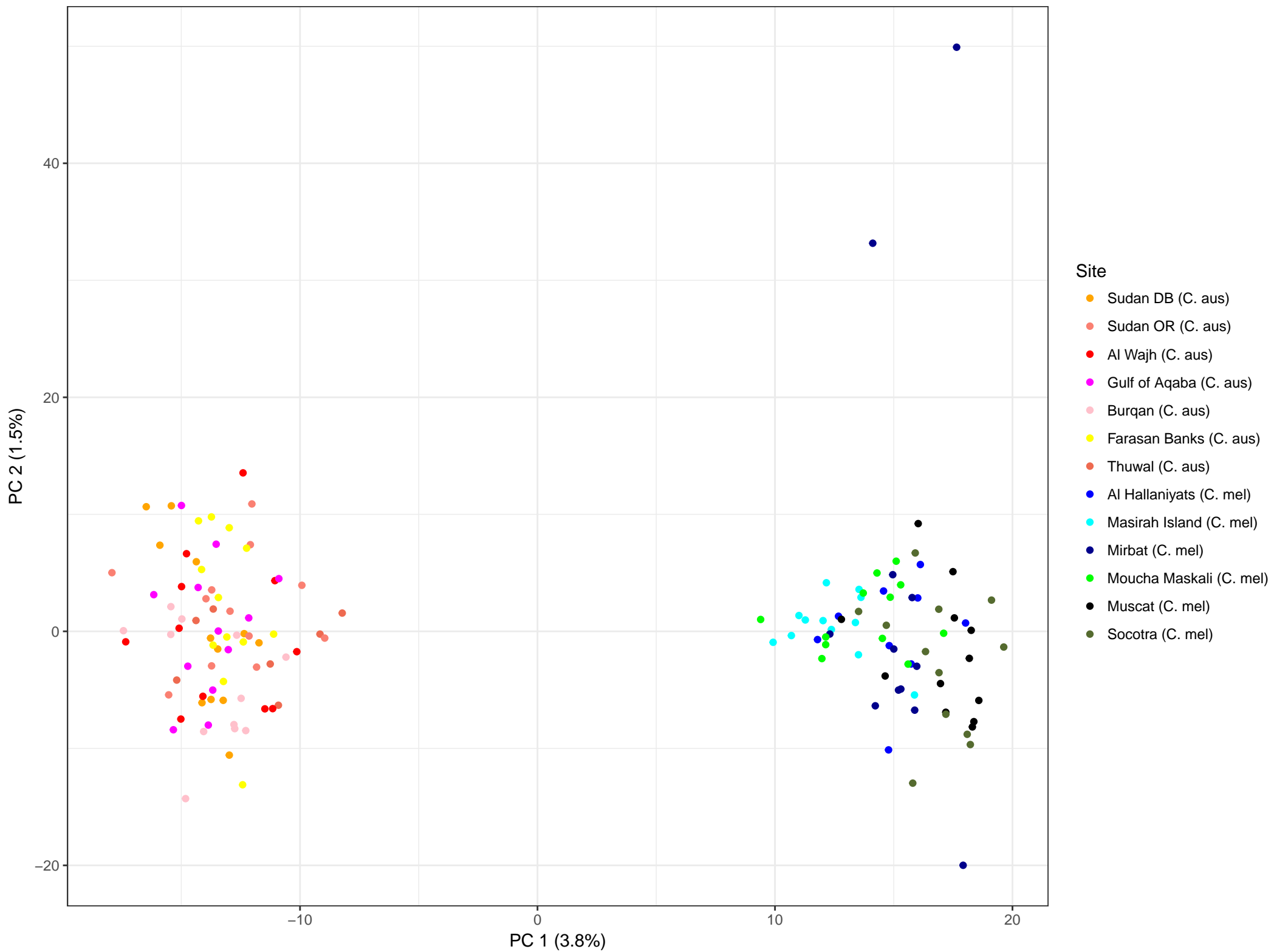

Supplement: Supplementary file 6 — Appendix S1 [file ECE3-10-4314-s006.zip › Appendix S1, STRUCTURE and PCA Plots, Dryad/PCA Plots/PCA, C. austriacus & C. melapterus.pdf]

*C. austriacus*

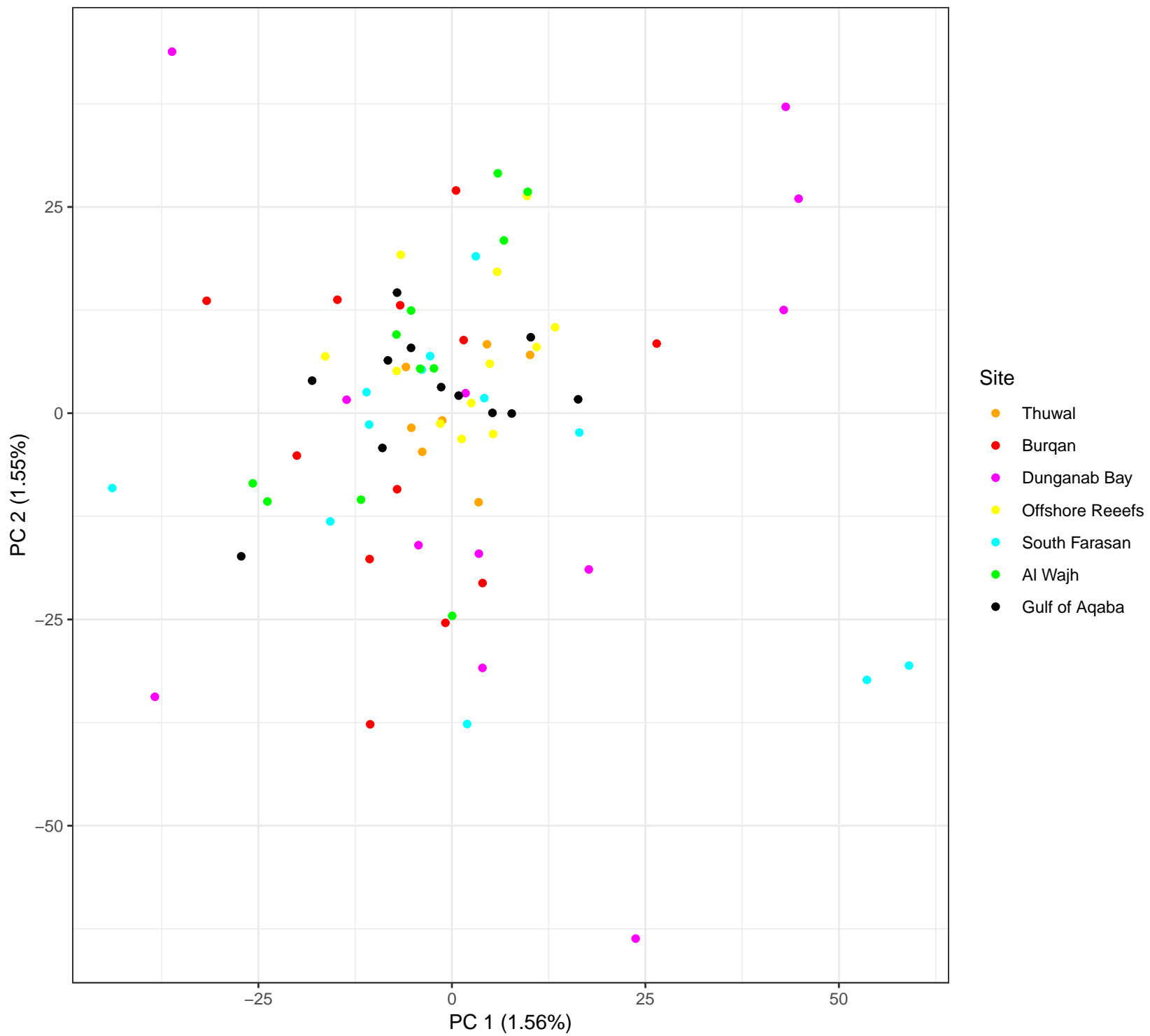

Supplement: Supplementary file 6 — Appendix S1 [file ECE3-10-4314-s006.zip › Appendix S1, STRUCTURE and PCA Plots, Dryad/PCA Plots/PCA, C. austriacus.pdf]

*C. fasciatus*

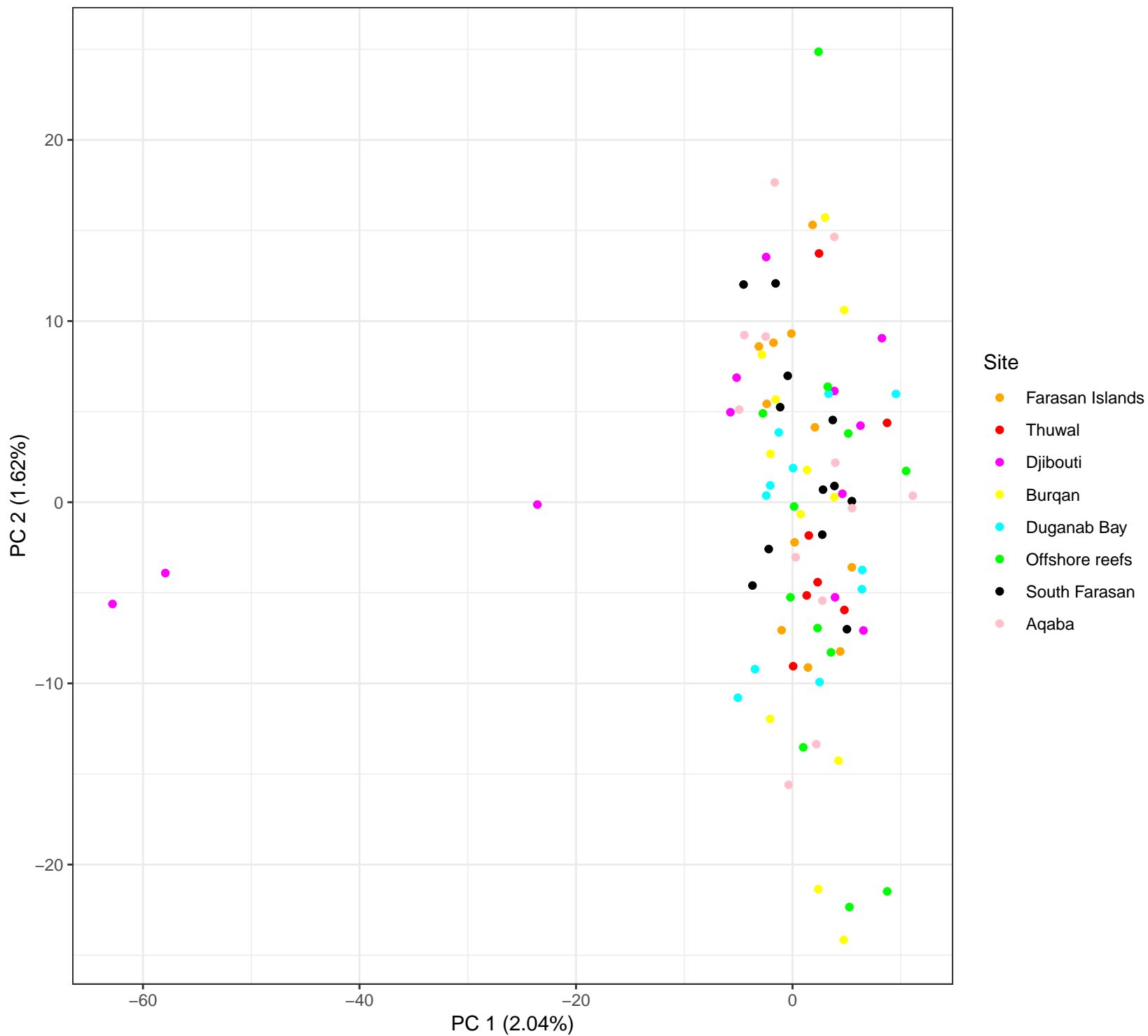

Supplement: Supplementary file 6 — Appendix S1 [file ECE3-10-4314-s006.zip › Appendix S1, STRUCTURE and PCA Plots, Dryad/PCA Plots/PCA, C. fasciatus.pdf]

*C. larvatus*

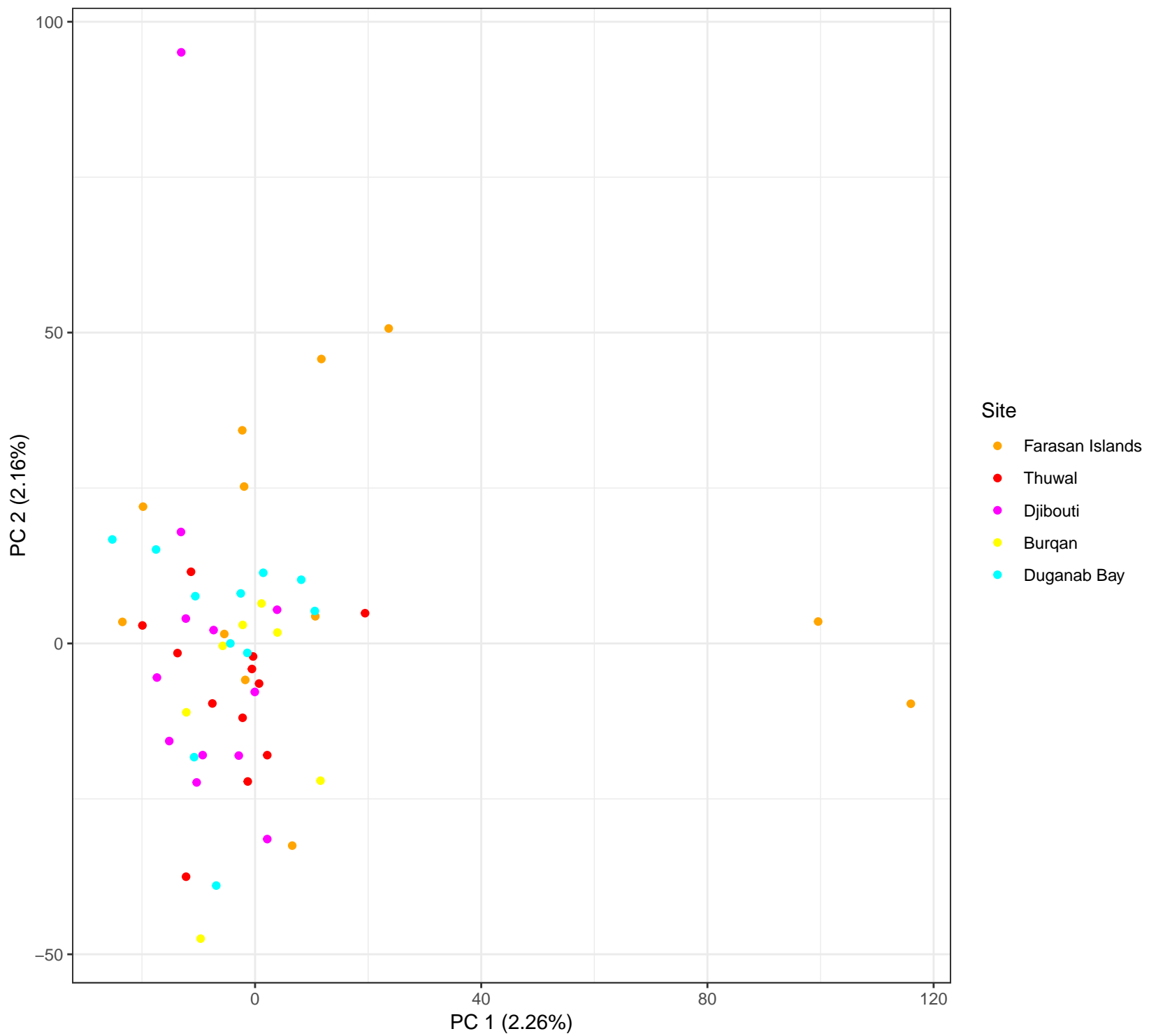

Supplement: Supplementary file 6 — Appendix S1 [file ECE3-10-4314-s006.zip › Appendix S1, STRUCTURE and PCA Plots, Dryad/PCA Plots/PCA, C. larvatus.pdf]

*C. melapтерus*

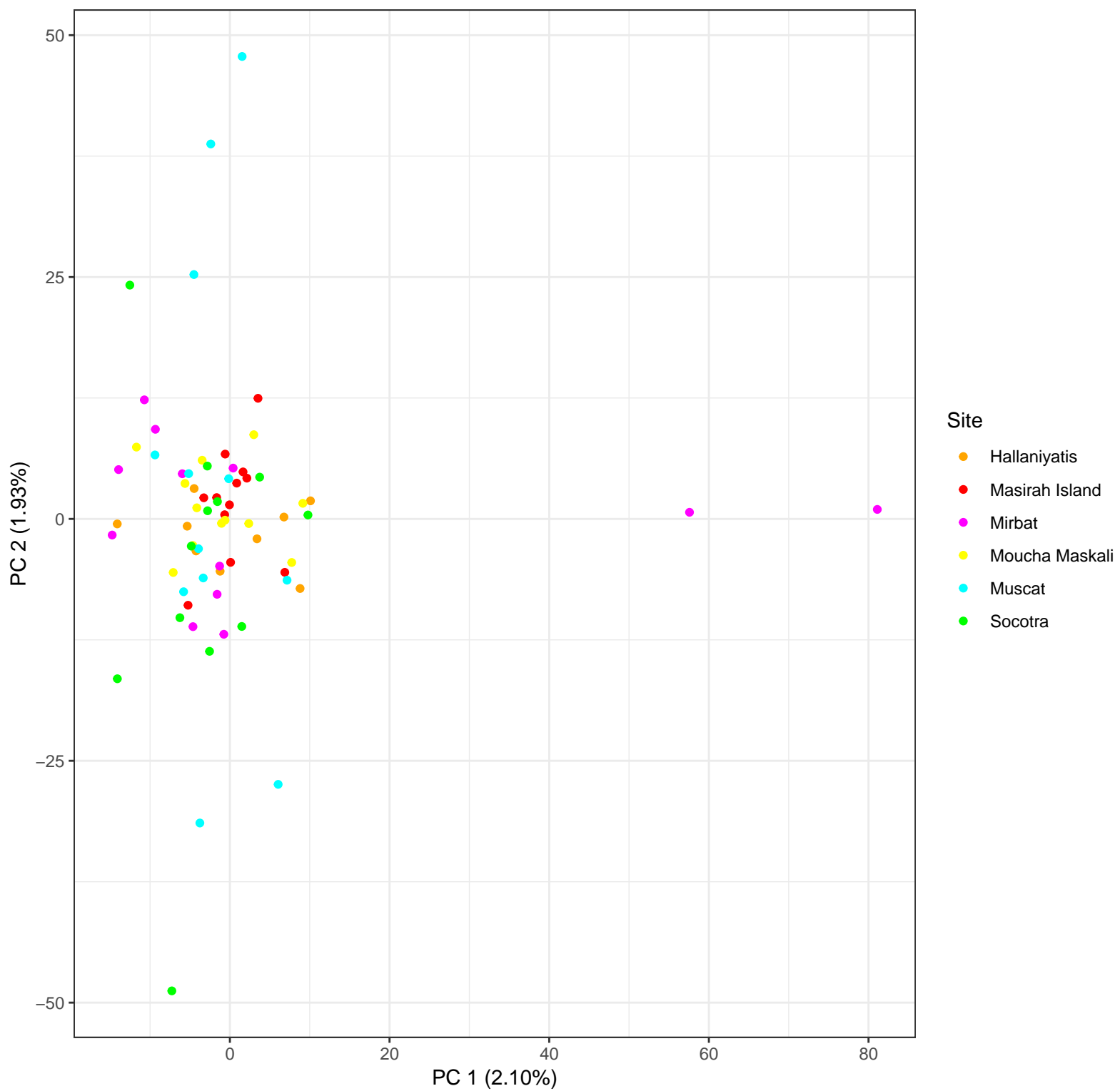

Supplement: Supplementary file 6 — Appendix S1 [file ECE3-10-4314-s006.zip › Appendix S1, STRUCTURE and PCA Plots, Dryad/PCA Plots/PCA, C. melapterus.pdf]

*C. mesoleucos*

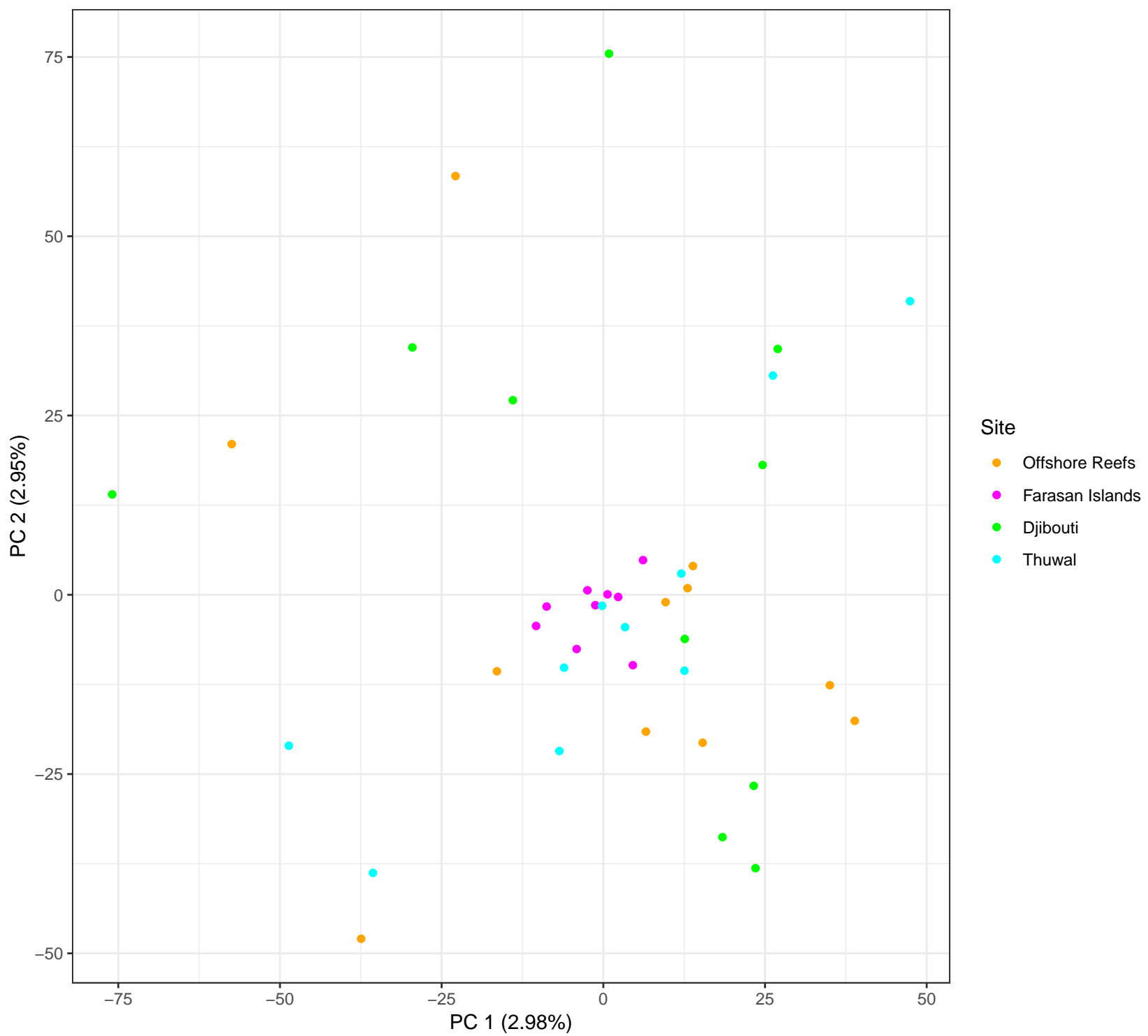

Supplement: Supplementary file 6 — Appendix S1 [file ECE3-10-4314-s006.zip › Appendix S1, STRUCTURE and PCA Plots, Dryad/PCA Plots/PCA, C. mesoleucos.pdf]

*C. paucifasciatus*

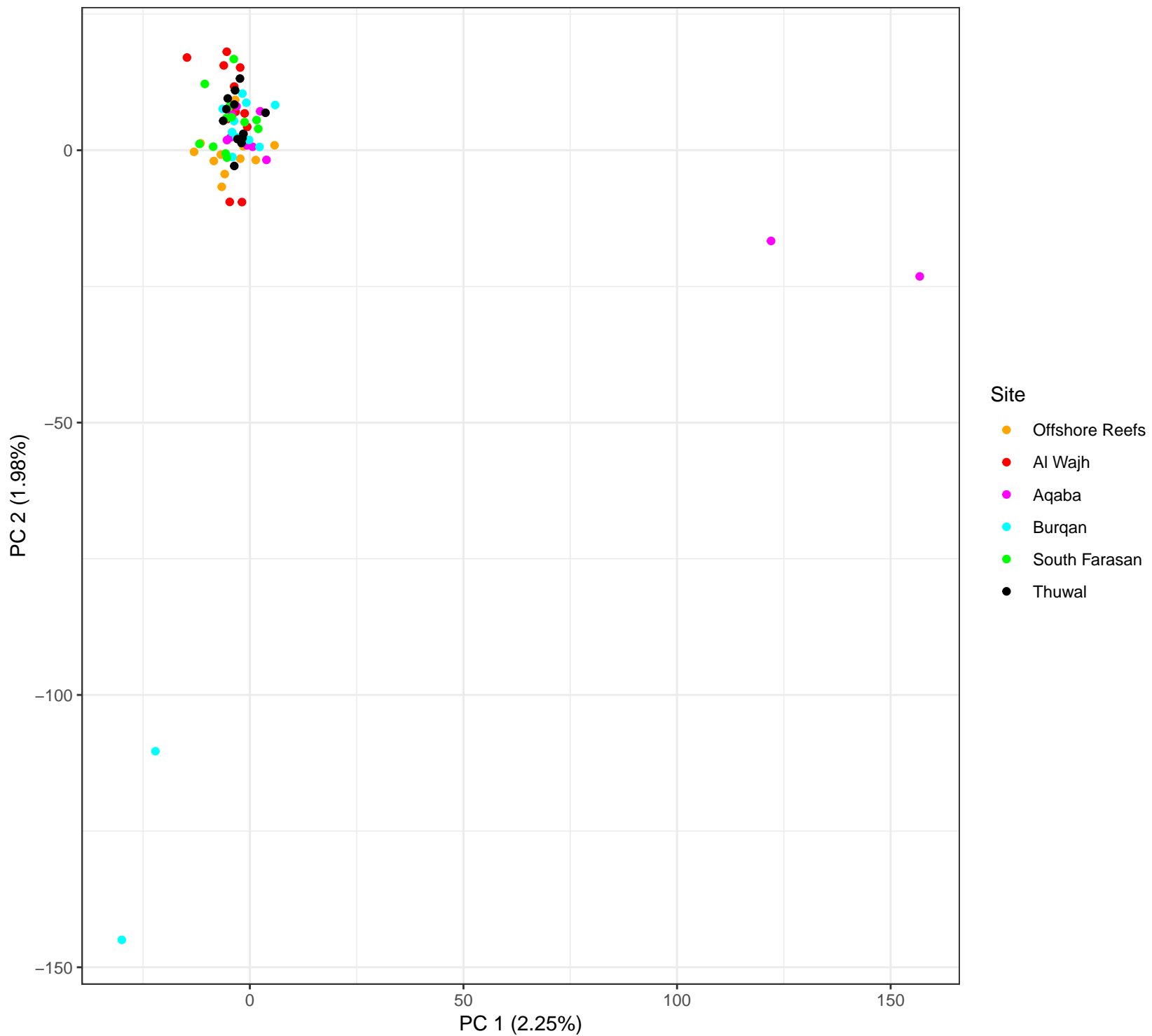

Supplement: Supplementary file 6 — Appendix S1 [file ECE3-10-4314-s006.zip › Appendix S1, STRUCTURE and PCA Plots, Dryad/PCA Plots/PCA, C. paucifasciatus.pdf]

*C. pictus*

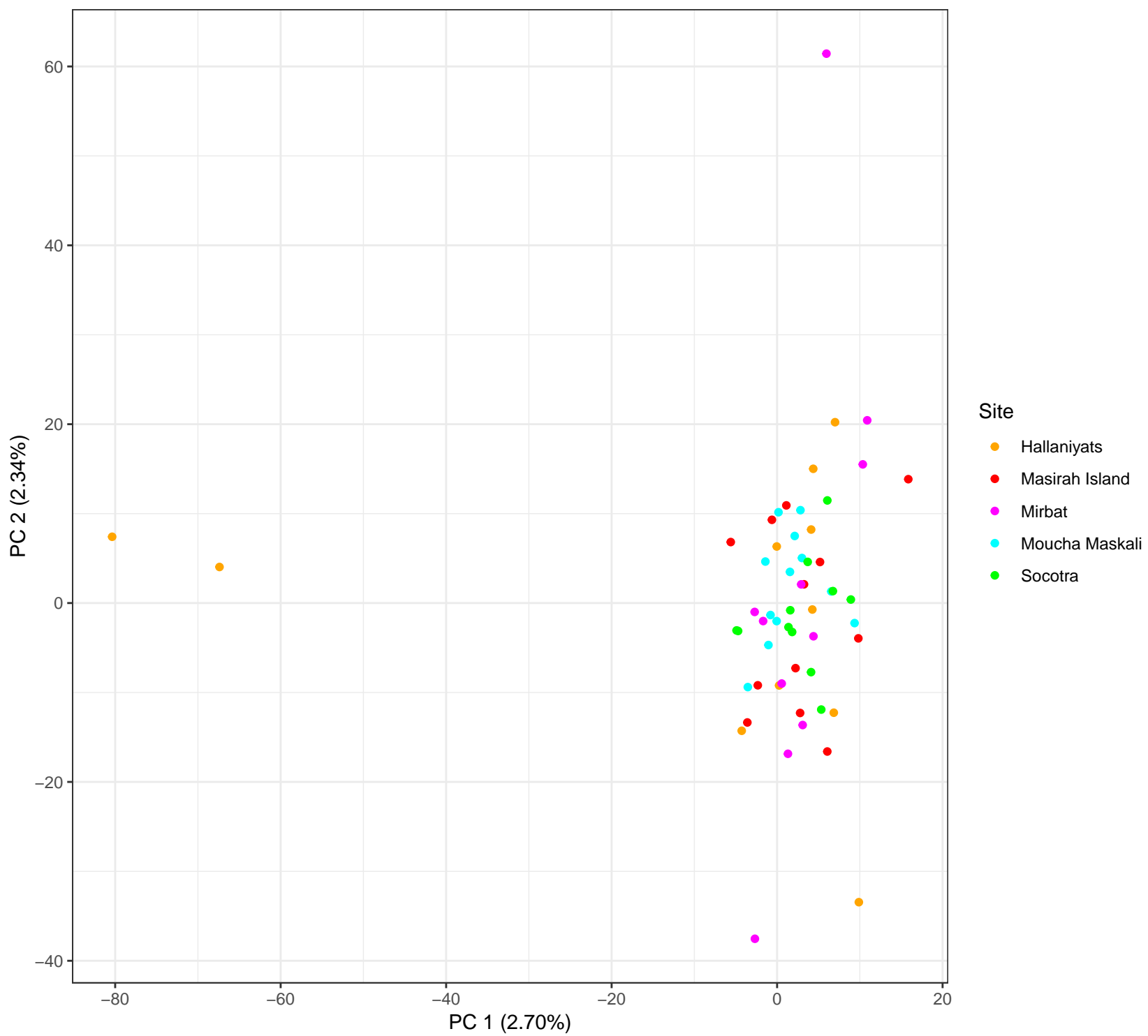

Supplement: Supplementary file 6 — Appendix S1 [file ECE3-10-4314-s006.zip › Appendix S1, STRUCTURE and PCA Plots, Dryad/PCA Plots/PCA, C. pictus.pdf]

*C. semilarvatus*

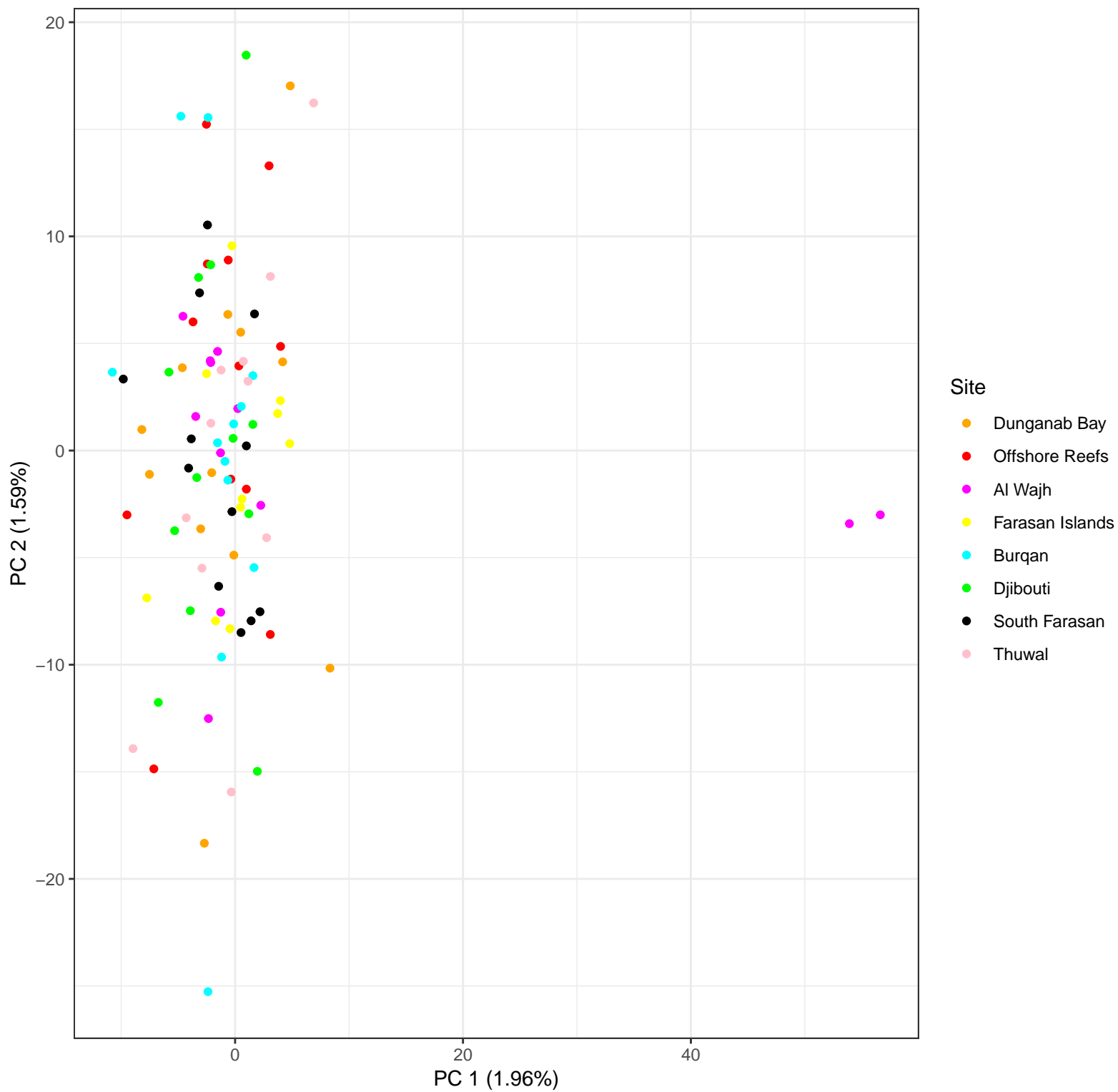

Supplement: Supplementary file 6 — Appendix S1 [file ECE3-10-4314-s006.zip › Appendix S1, STRUCTURE and PCA Plots, Dryad/PCA Plots/PCA, C. semilarvatus.pdf]

*C. trifascialis*

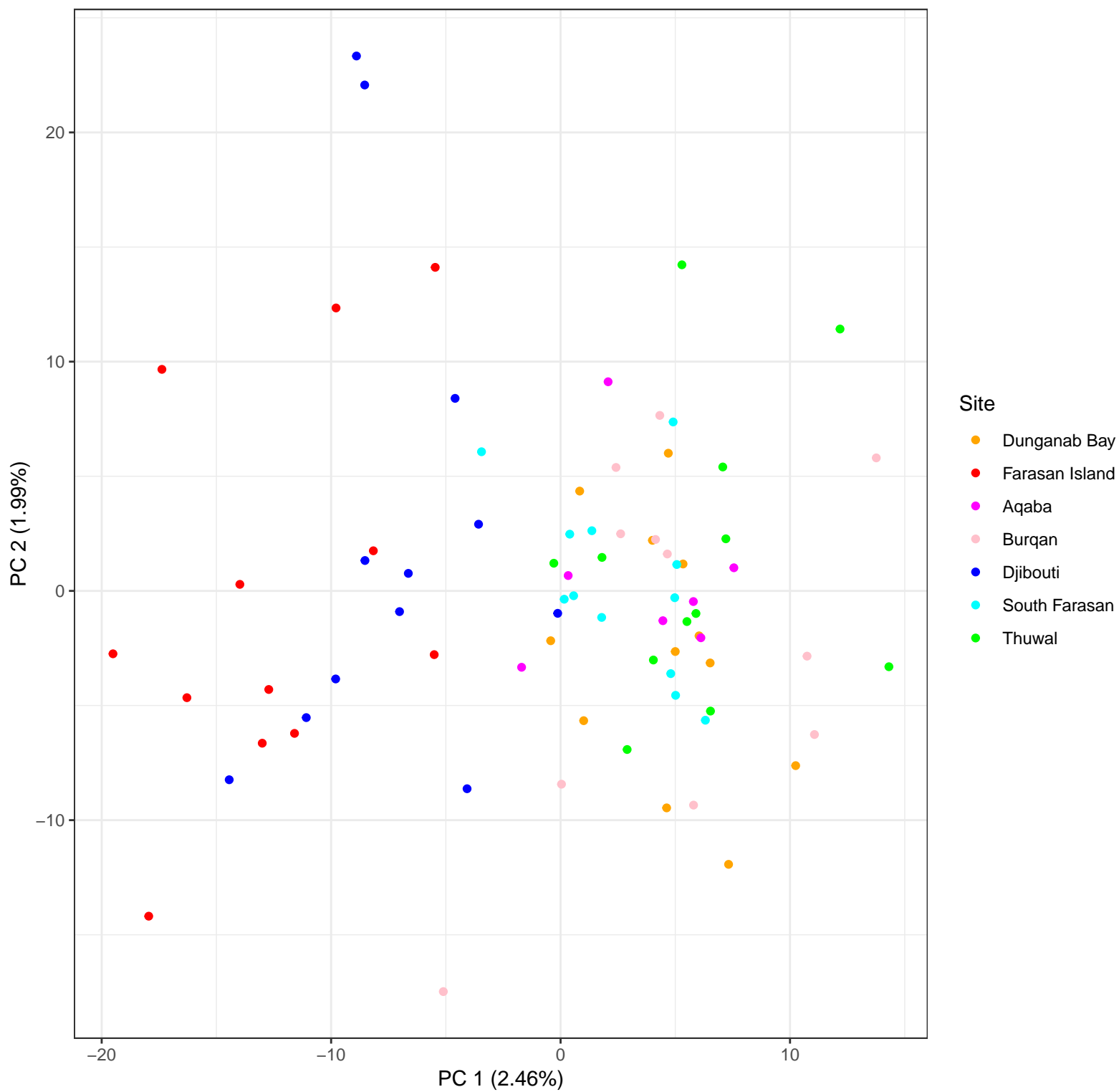

Supplement: Supplementary file 6 — Appendix S1 [file ECE3-10-4314-s006.zip › Appendix S1, STRUCTURE and PCA Plots, Dryad/PCA Plots/PCA, C. trifascialis, Red Sea sites only.pdf]

*C. trifascialis*

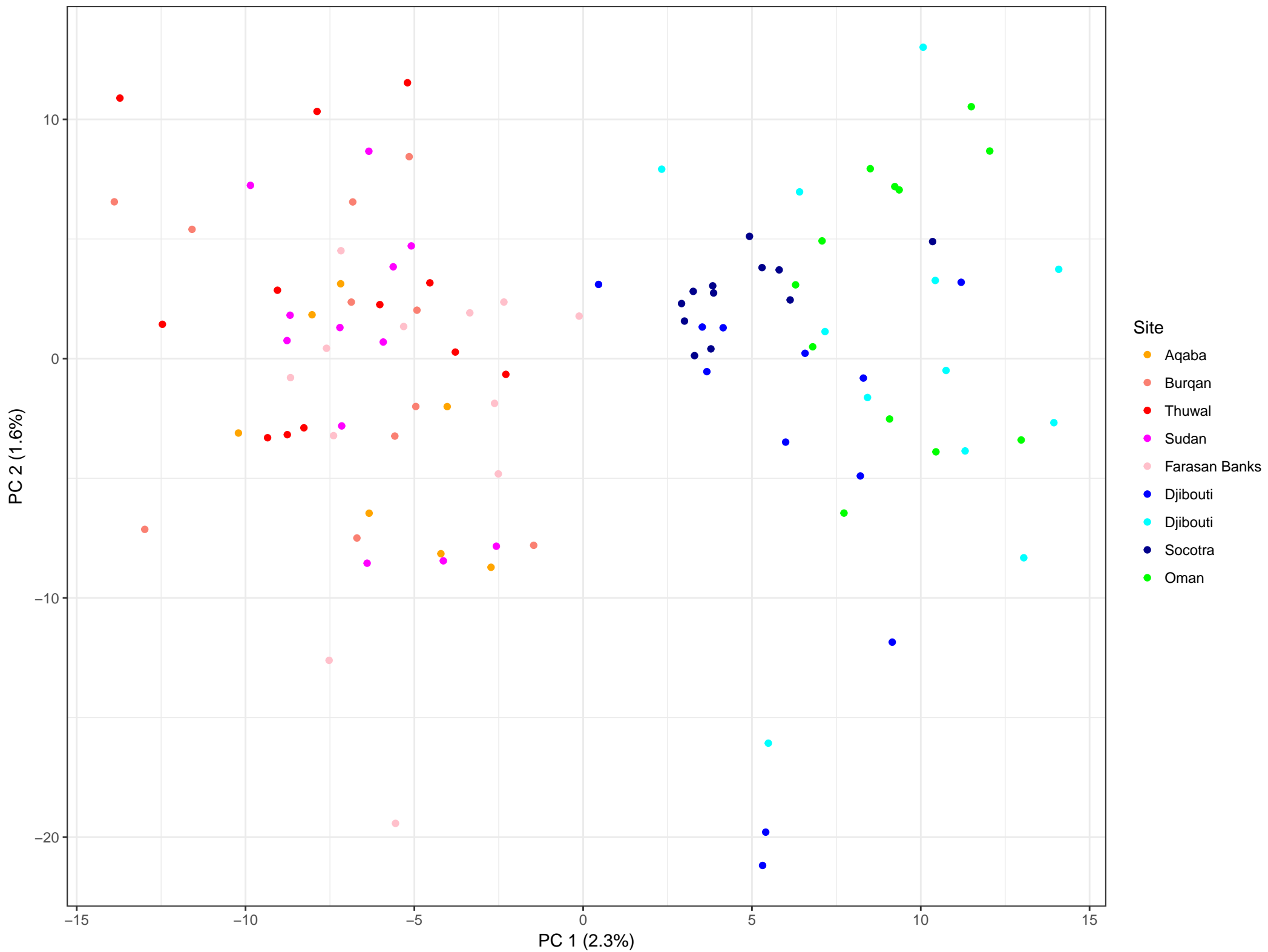

Supplement: Supplementary file 6 — Appendix S1 [file ECE3-10-4314-s006.zip › Appendix S1, STRUCTURE and PCA Plots, Dryad/PCA Plots/PCA, C. trifascialis.pdf]

Ct. striatus

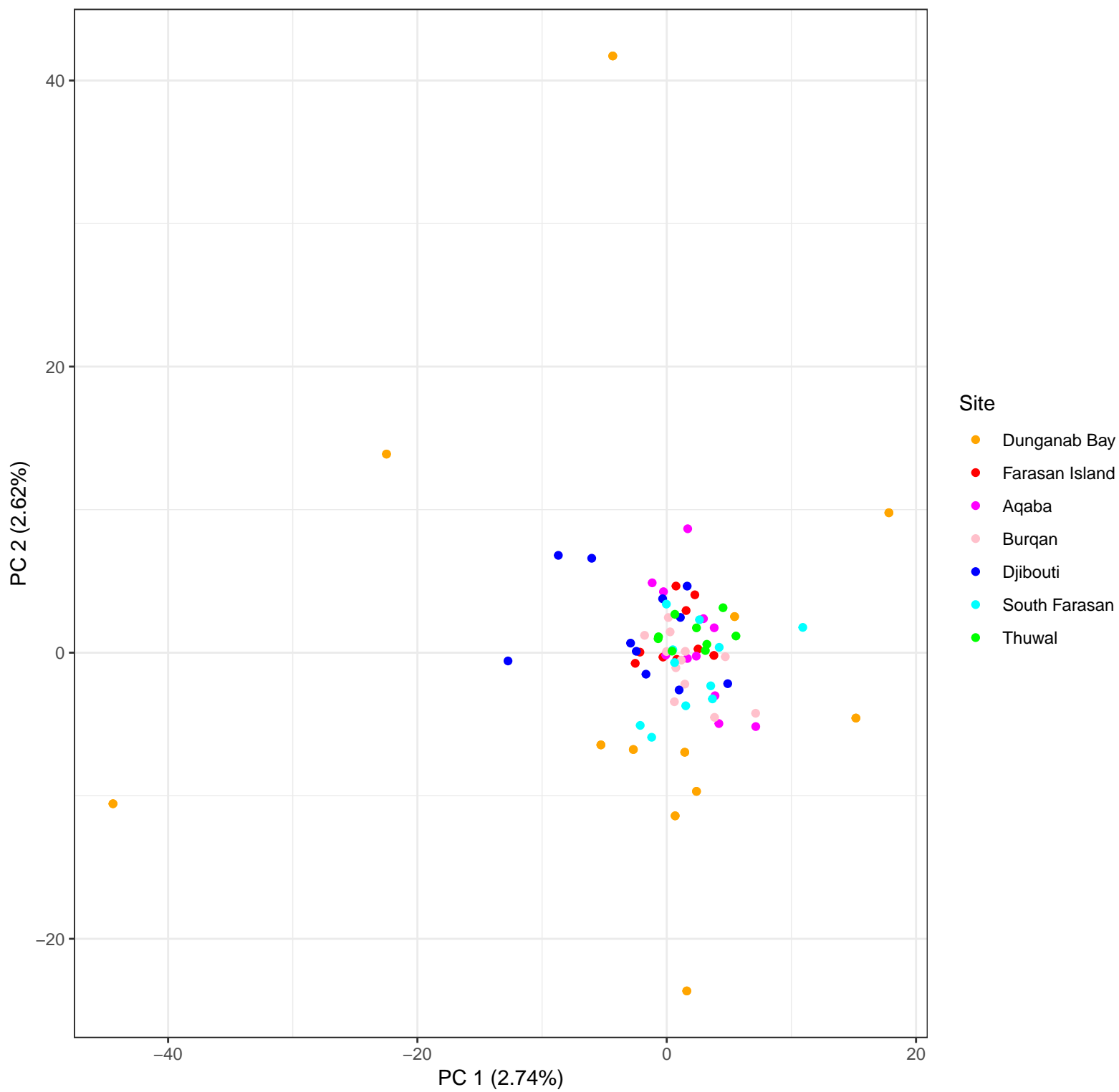

Supplement: Supplementary file 6 — Appendix S1 [file ECE3-10-4314-s006.zip › Appendix S1, STRUCTURE and PCA Plots, Dryad/PCA Plots/PCA, Ct. striatus, Red Sea sites only.pdf]

Ct. striatus

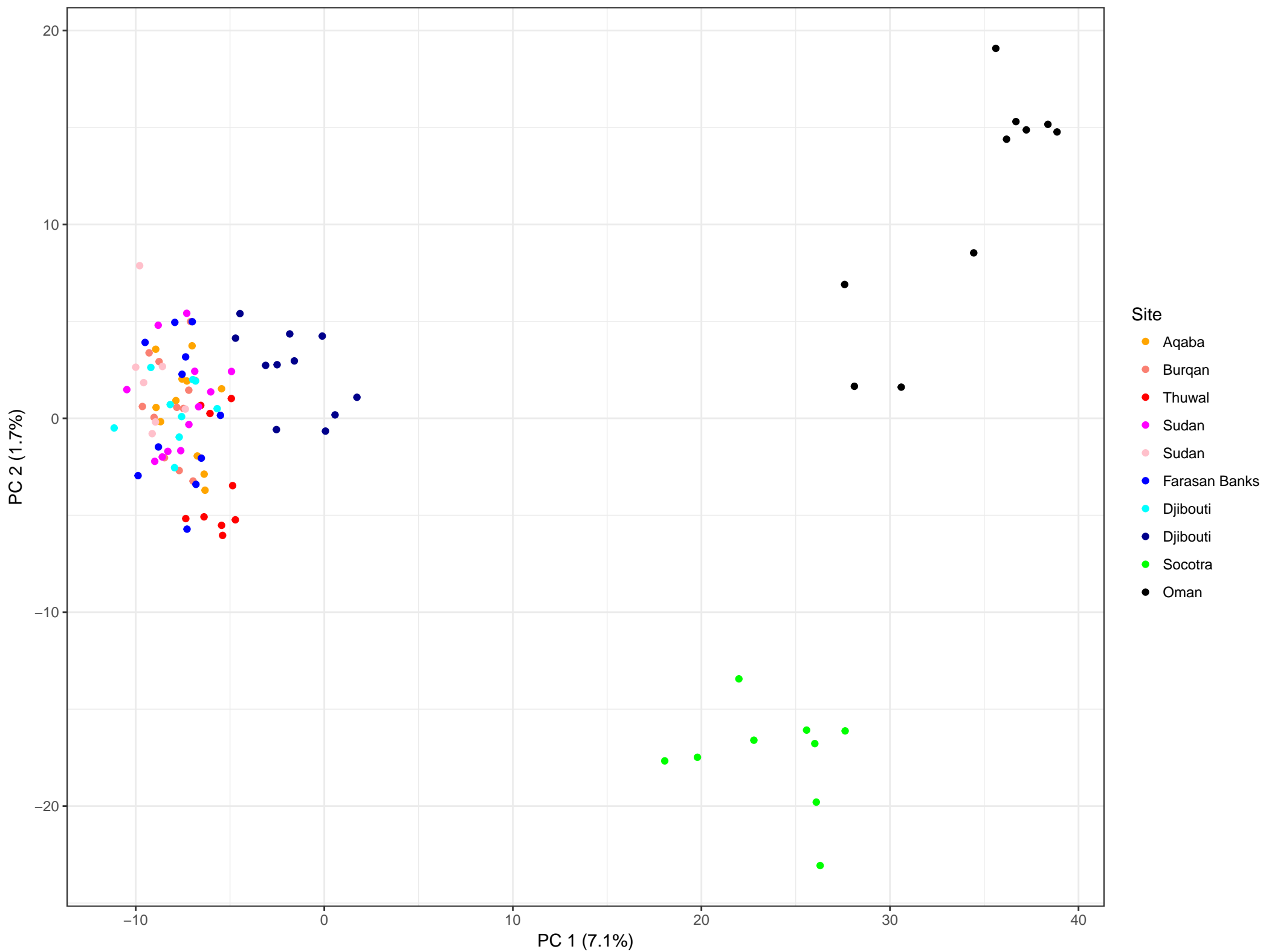

Supplement: Supplementary file 6 — Appendix S1 [file ECE3-10-4314-s006.zip › Appendix S1, STRUCTURE and PCA Plots, Dryad/PCA Plots/PCA, Ct. striatus.pdf]

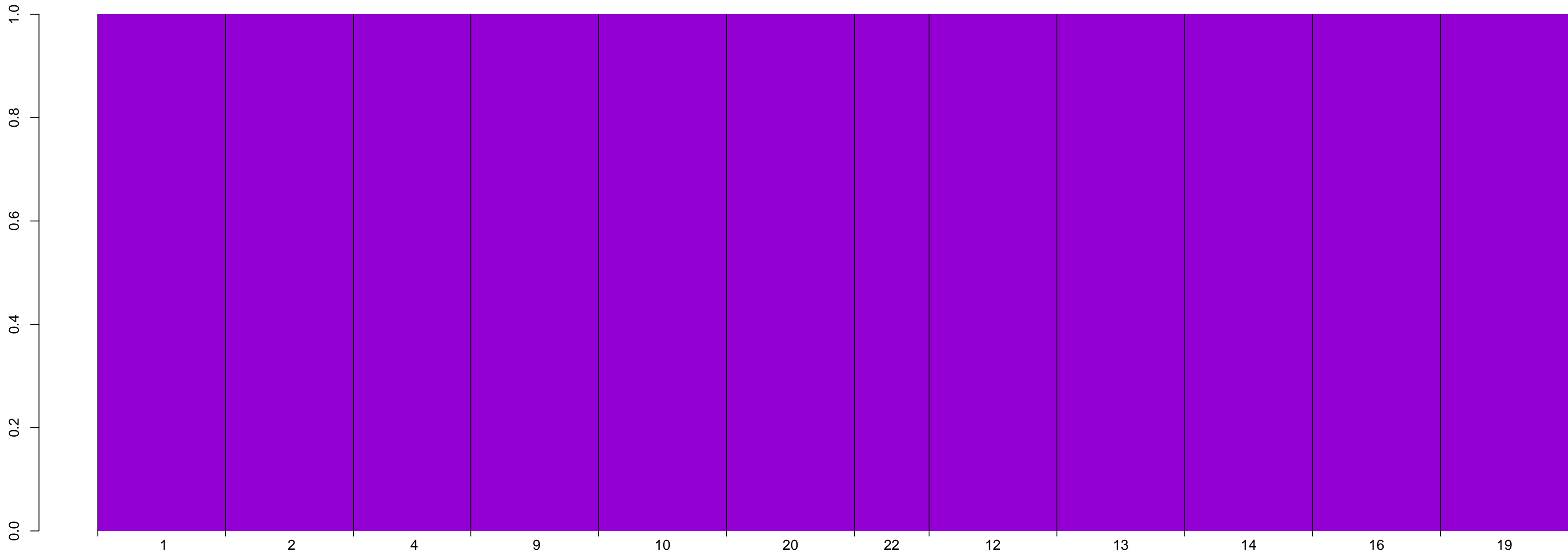

Supplement: Supplementary file 6 — Appendix S1 [file ECE3-10-4314-s006.zip › Appendix S1, STRUCTURE and PCA Plots, Dryad/STRUCTURE/C. austriacus & C. melapterus/job_T1.pdf]

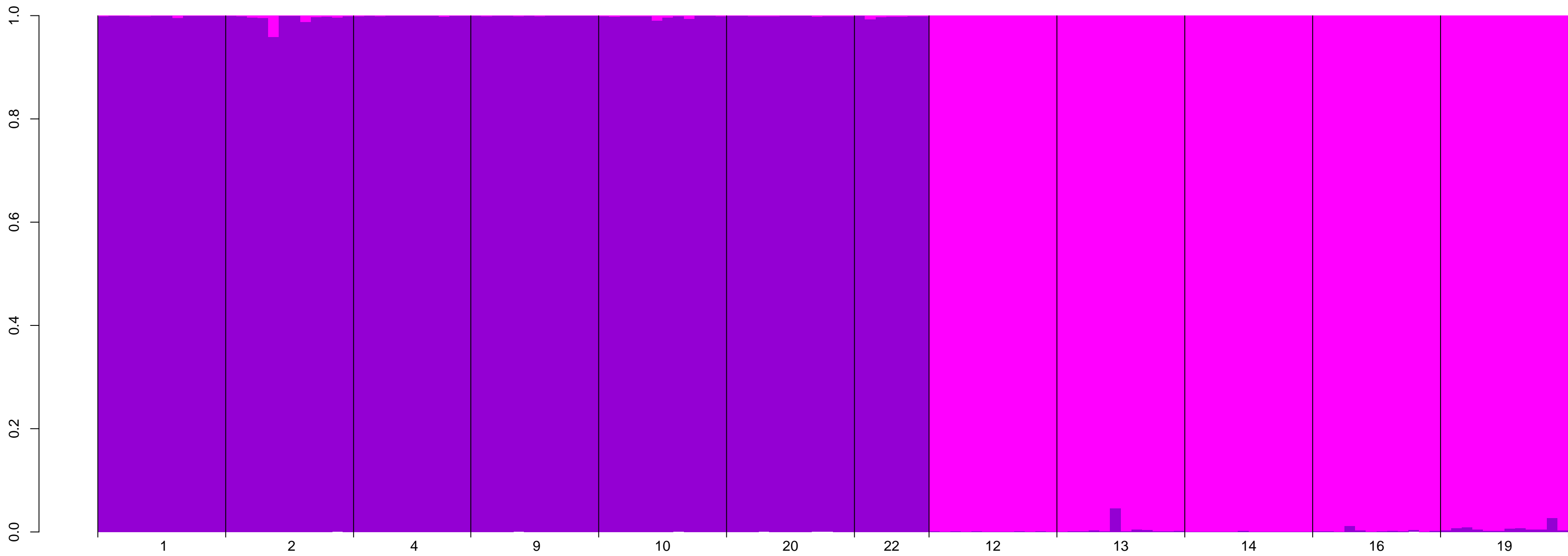

Supplement: Supplementary file 6 — Appendix S1 [file ECE3-10-4314-s006.zip › Appendix S1, STRUCTURE and PCA Plots, Dryad/STRUCTURE/C. austriacus & C. melapterus/job_T10.pdf]

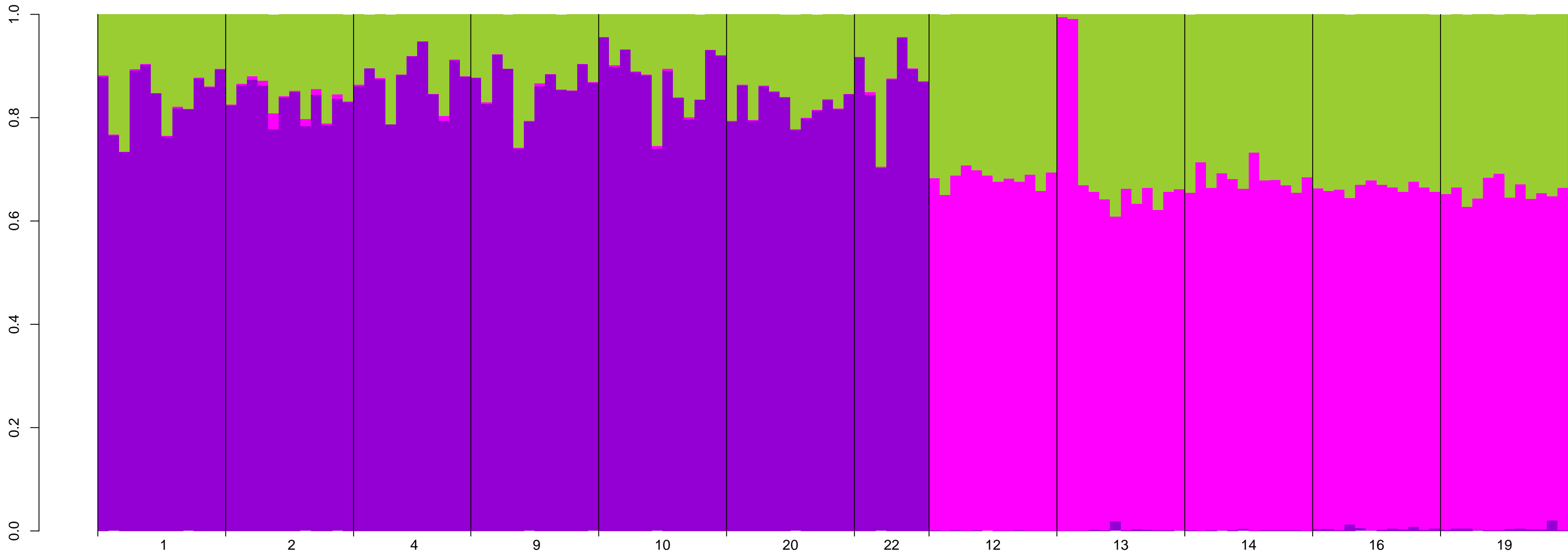

Supplement: Supplementary file 6 — Appendix S1 [file ECE3-10-4314-s006.zip › Appendix S1, STRUCTURE and PCA Plots, Dryad/STRUCTURE/C. austriacus & C. melapterus/job_T11.pdf]

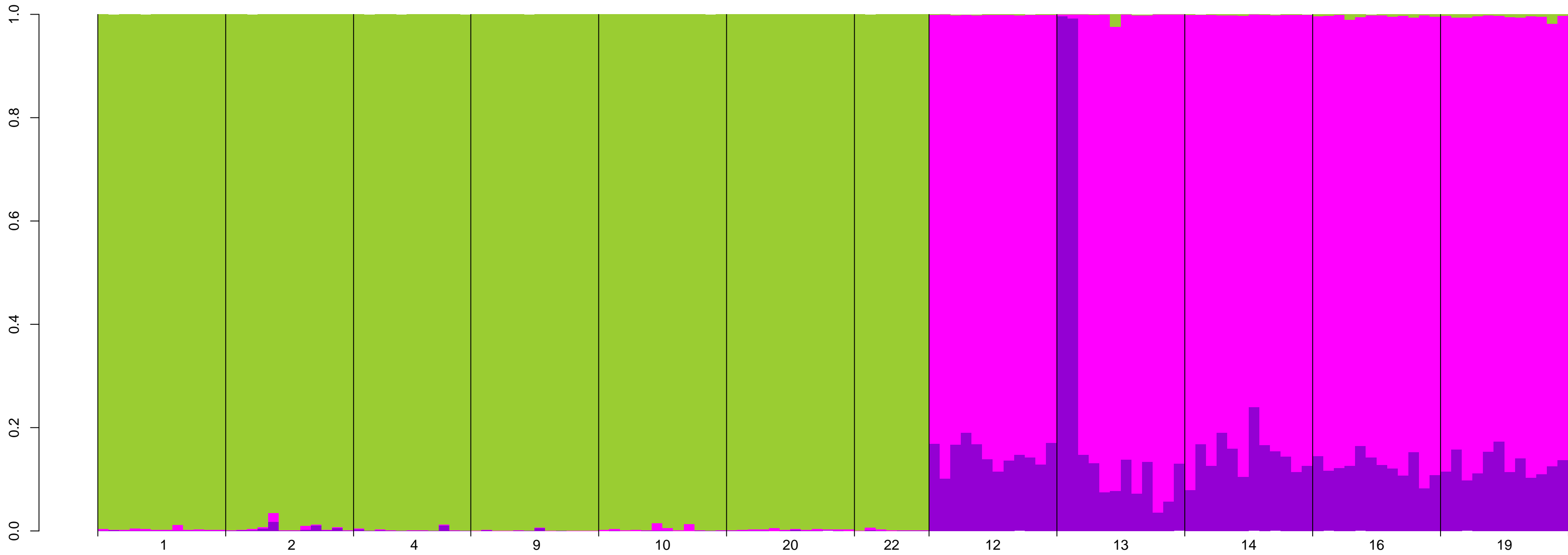

Supplement: Supplementary file 6 — Appendix S1 [file ECE3-10-4314-s006.zip › Appendix S1, STRUCTURE and PCA Plots, Dryad/STRUCTURE/C. austriacus & C. melapterus/job_T12.pdf]

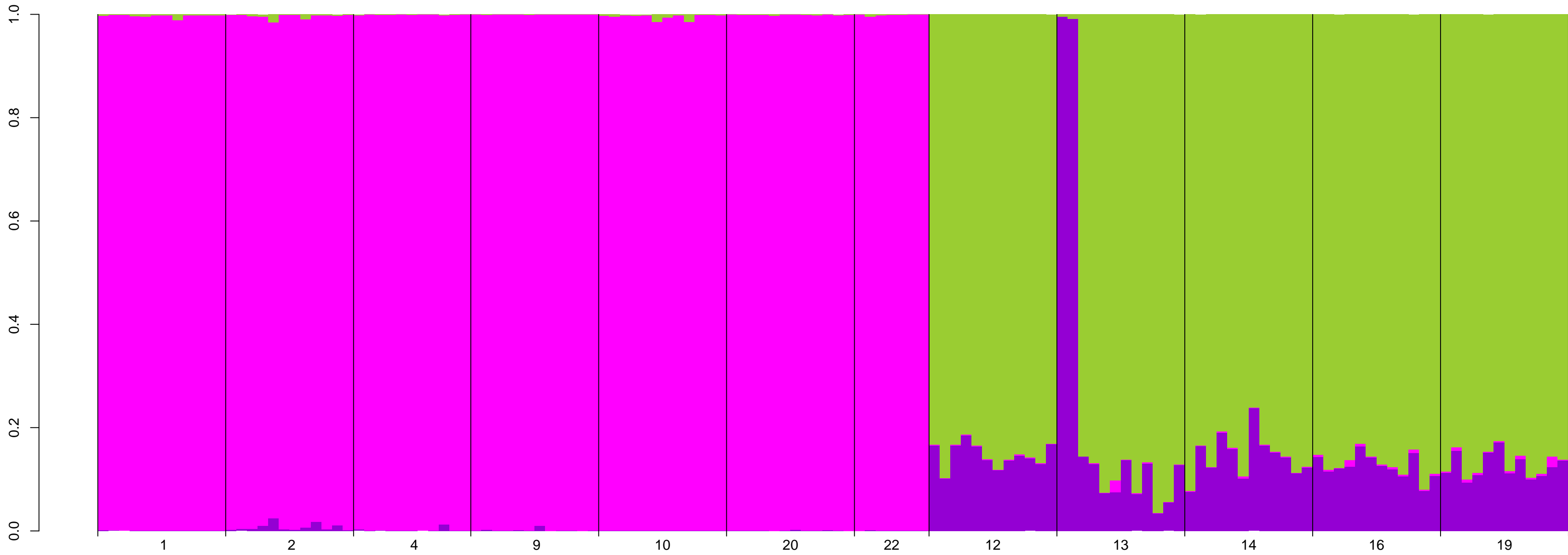

Supplement: Supplementary file 6 — Appendix S1 [file ECE3-10-4314-s006.zip › Appendix S1, STRUCTURE and PCA Plots, Dryad/STRUCTURE/C. austriacus & C. melapterus/job_T13.pdf]

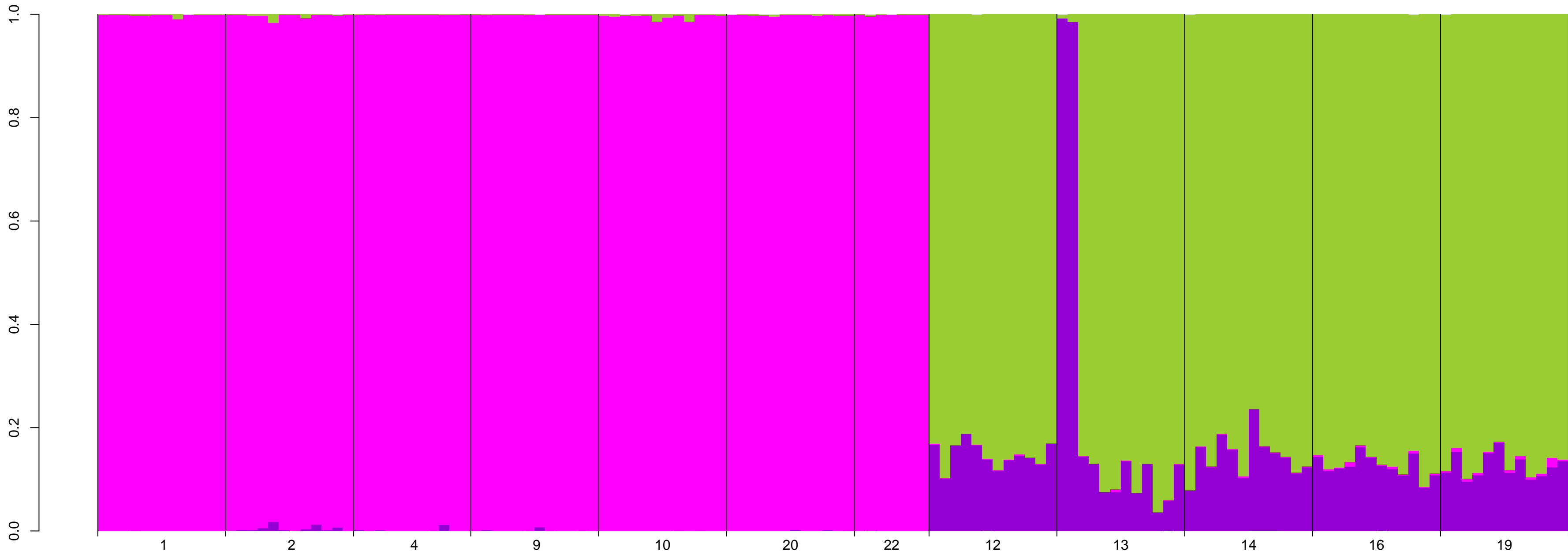

Supplement: Supplementary file 6 — Appendix S1 [file ECE3-10-4314-s006.zip › Appendix S1, STRUCTURE and PCA Plots, Dryad/STRUCTURE/C. austriacus & C. melapterus/job_T14.pdf]

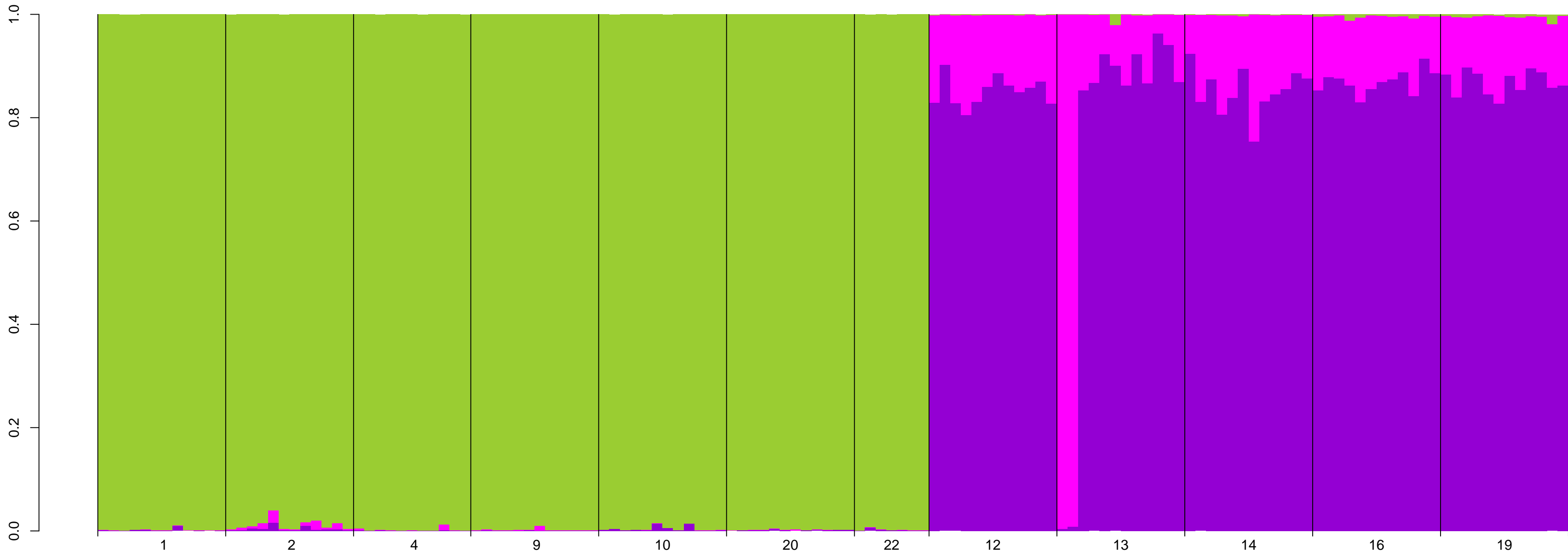

Supplement: Supplementary file 6 — Appendix S1 [file ECE3-10-4314-s006.zip › Appendix S1, STRUCTURE and PCA Plots, Dryad/STRUCTURE/C. austriacus & C. melapterus/job_T15.pdf]

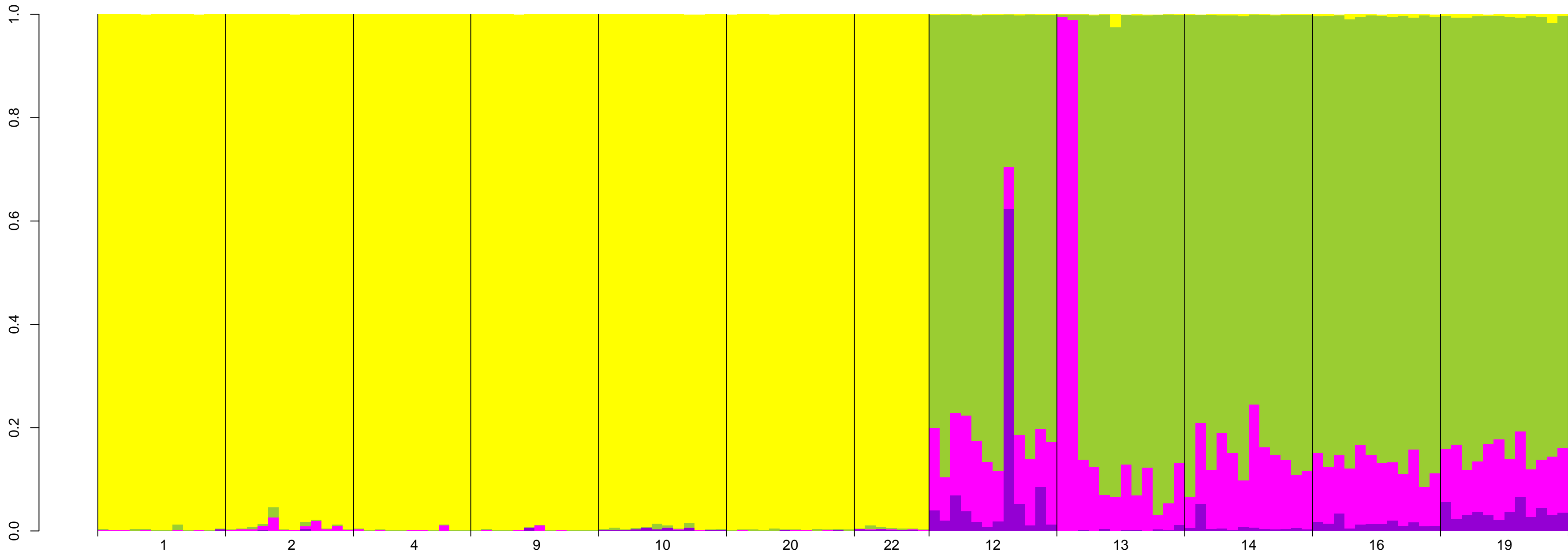

Supplement: Supplementary file 6 — Appendix S1 [file ECE3-10-4314-s006.zip › Appendix S1, STRUCTURE and PCA Plots, Dryad/STRUCTURE/C. austriacus & C. melapterus/job_T16.pdf]

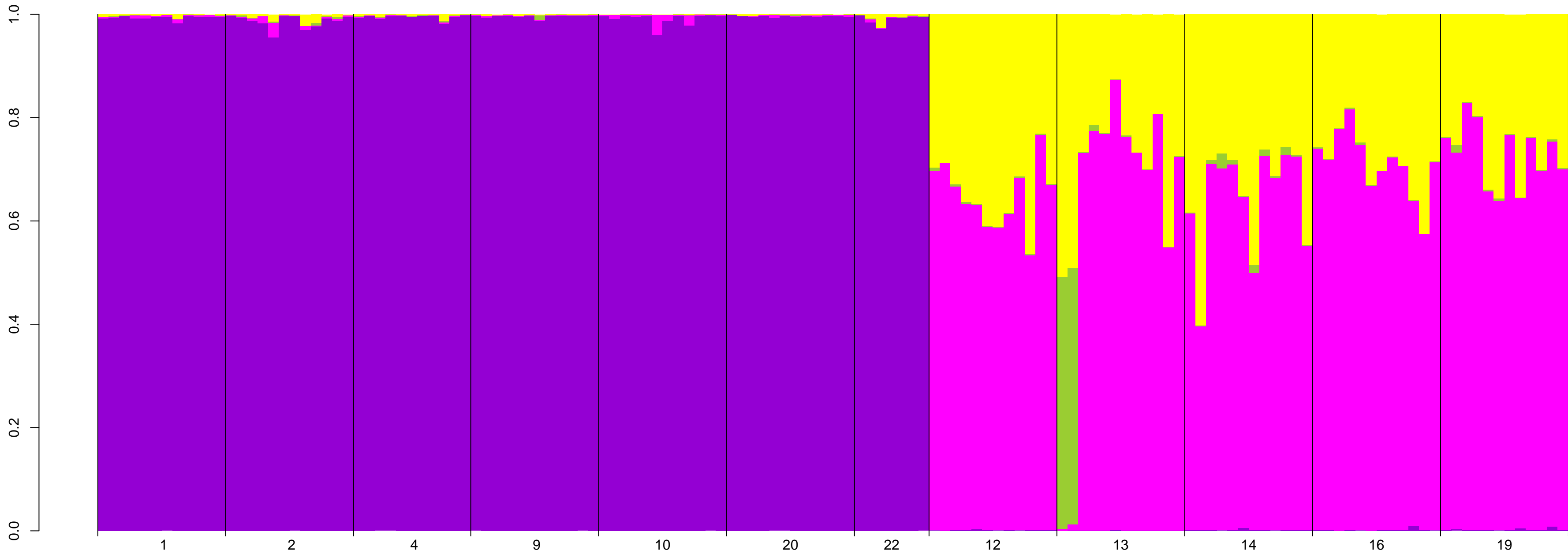

Supplement: Supplementary file 6 — Appendix S1 [file ECE3-10-4314-s006.zip › Appendix S1, STRUCTURE and PCA Plots, Dryad/STRUCTURE/C. austriacus & C. melapterus/job_T17.pdf]

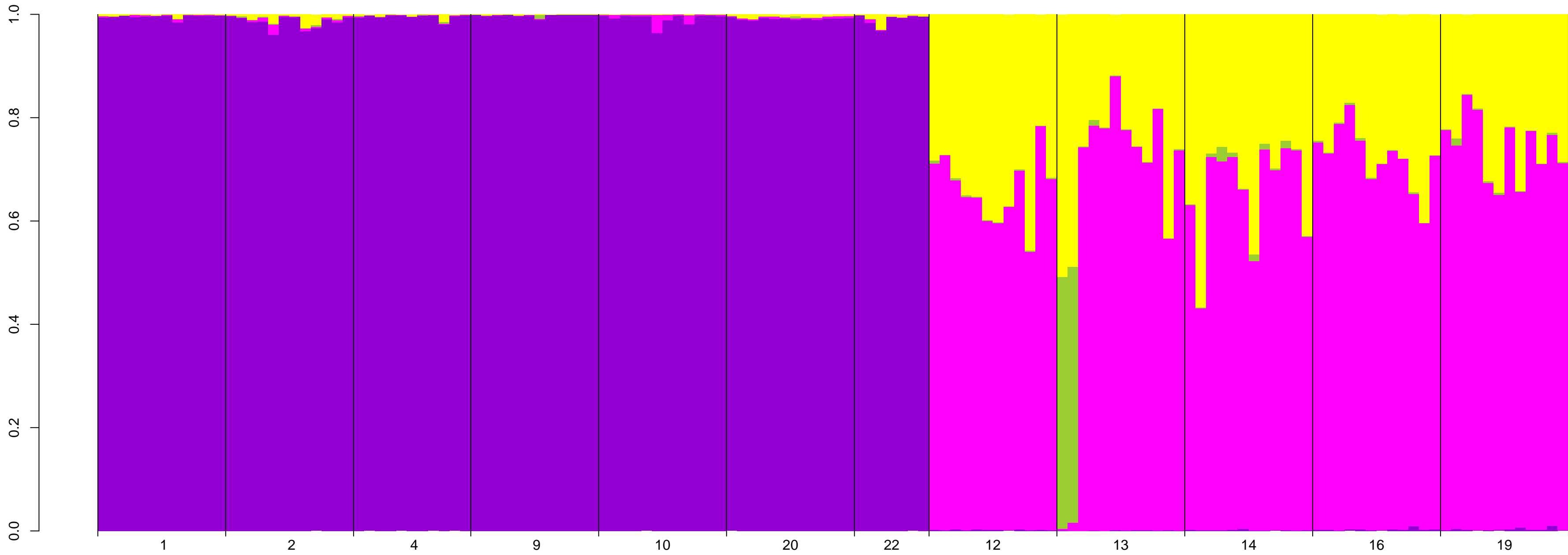

Supplement: Supplementary file 6 — Appendix S1 [file ECE3-10-4314-s006.zip › Appendix S1, STRUCTURE and PCA Plots, Dryad/STRUCTURE/C. austriacus & C. melapterus/job_T18.pdf]

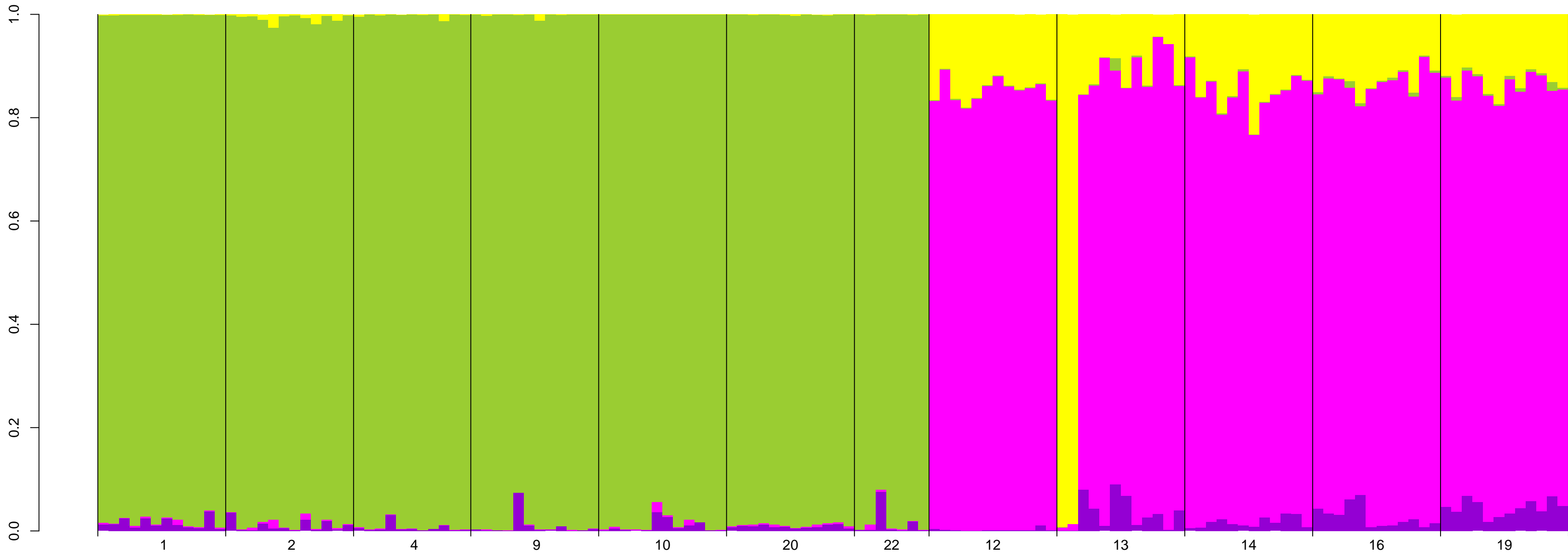

Supplement: Supplementary file 6 — Appendix S1 [file ECE3-10-4314-s006.zip › Appendix S1, STRUCTURE and PCA Plots, Dryad/STRUCTURE/C. austriacus & C. melapterus/job_T19.pdf]

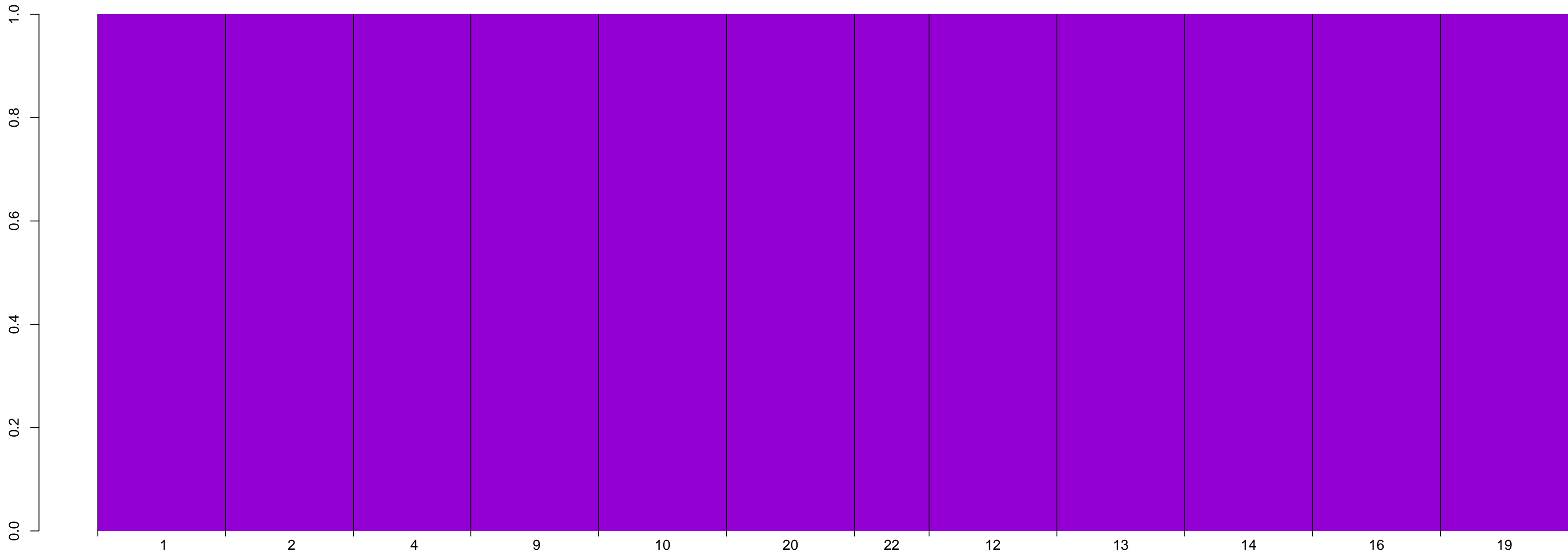

Supplement: Supplementary file 6 — Appendix S1 [file ECE3-10-4314-s006.zip › Appendix S1, STRUCTURE and PCA Plots, Dryad/STRUCTURE/C. austriacus & C. melapterus/job_T2.pdf]

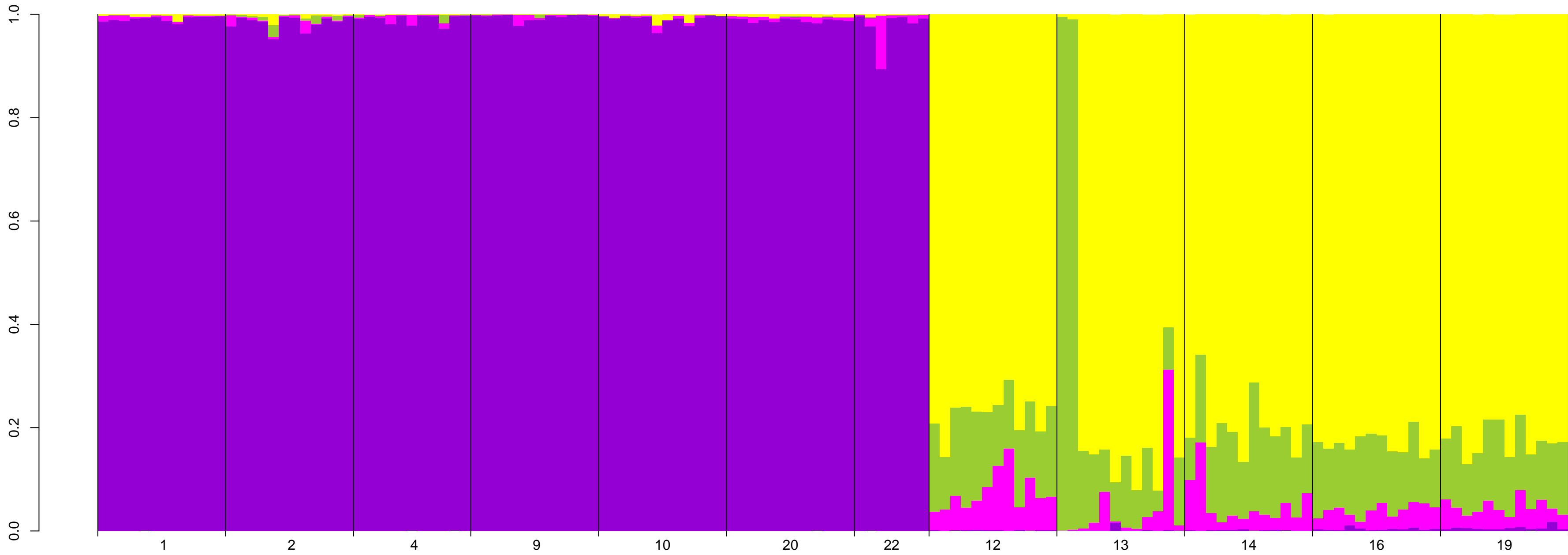

Supplement: Supplementary file 6 — Appendix S1 [file ECE3-10-4314-s006.zip › Appendix S1, STRUCTURE and PCA Plots, Dryad/STRUCTURE/C. austriacus & C. melapterus/job_T20.pdf]

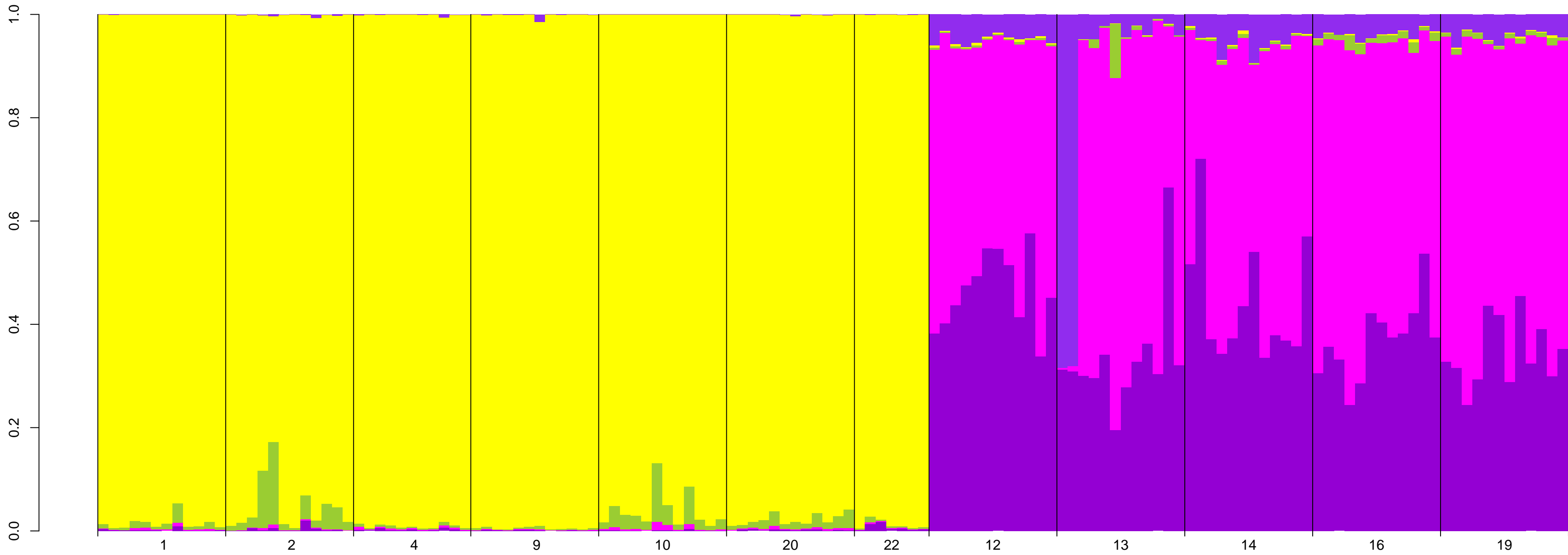

Supplement: Supplementary file 6 — Appendix S1 [file ECE3-10-4314-s006.zip › Appendix S1, STRUCTURE and PCA Plots, Dryad/STRUCTURE/C. austriacus & C. melapterus/job_T21.pdf]

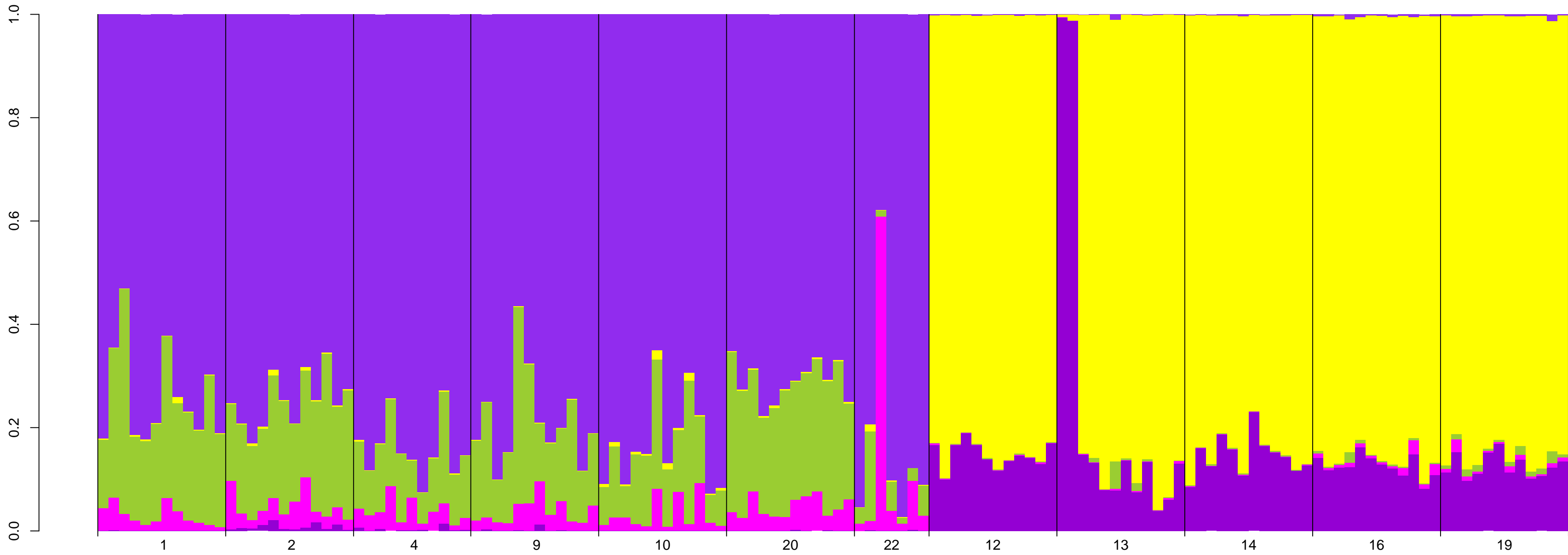

Supplement: Supplementary file 6 — Appendix S1 [file ECE3-10-4314-s006.zip › Appendix S1, STRUCTURE and PCA Plots, Dryad/STRUCTURE/C. austriacus & C. melapterus/job_T22.pdf]

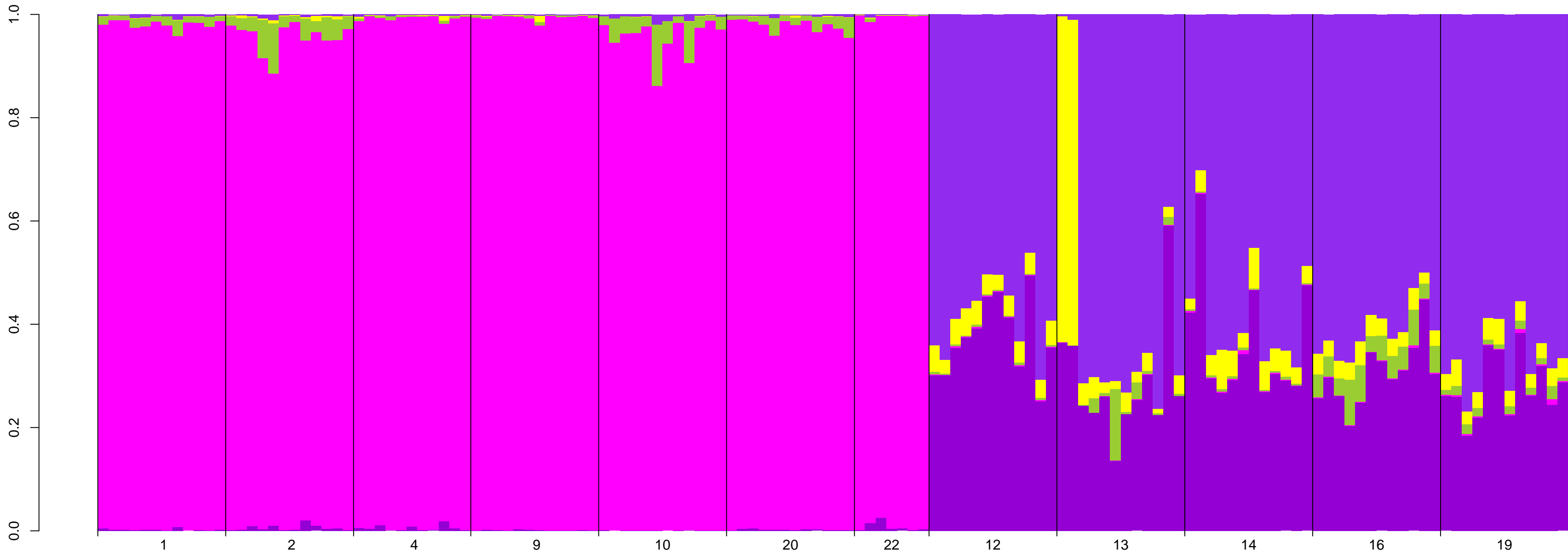

Supplement: Supplementary file 6 — Appendix S1 [file ECE3-10-4314-s006.zip › Appendix S1, STRUCTURE and PCA Plots, Dryad/STRUCTURE/C. austriacus & C. melapterus/job_T23.pdf]

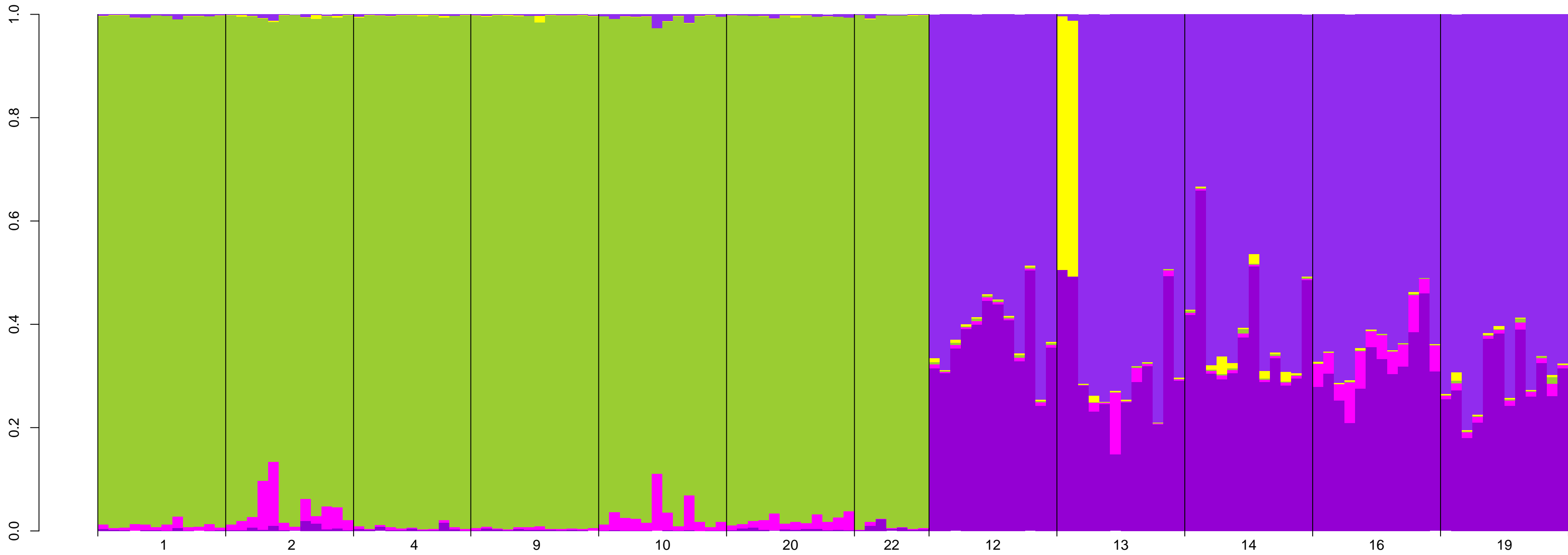

Supplement: Supplementary file 6 — Appendix S1 [file ECE3-10-4314-s006.zip › Appendix S1, STRUCTURE and PCA Plots, Dryad/STRUCTURE/C. austriacus & C. melapterus/job_T24.pdf]

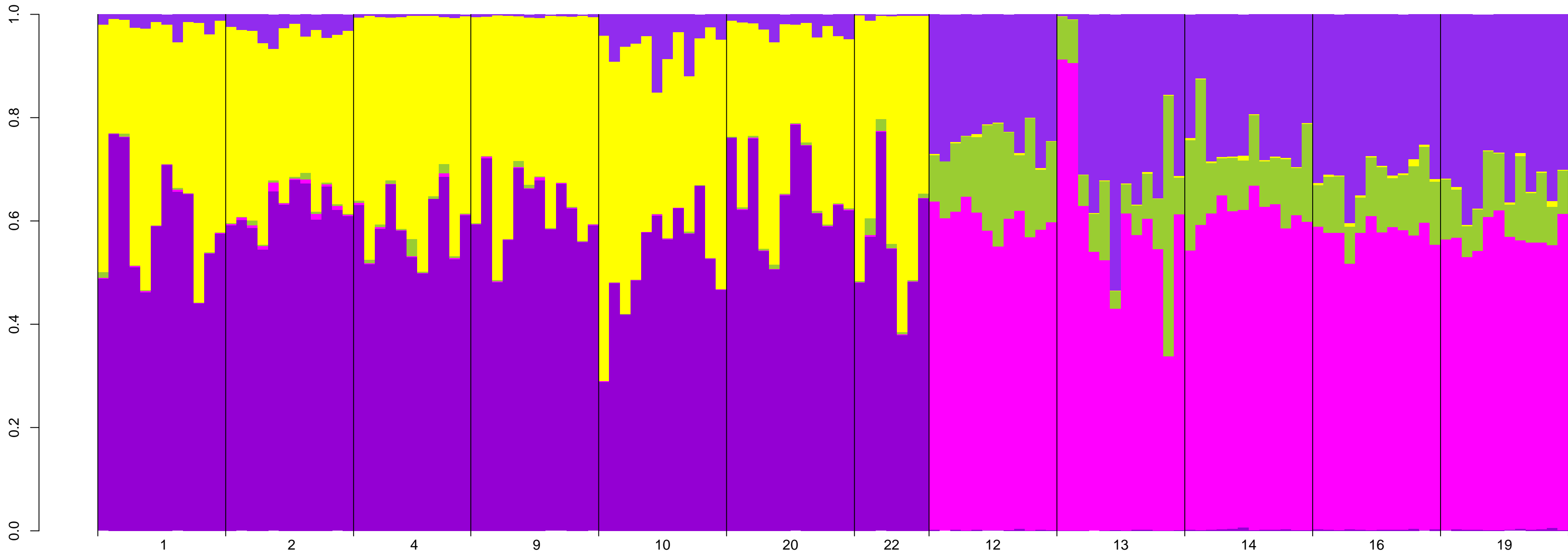

Supplement: Supplementary file 6 — Appendix S1 [file ECE3-10-4314-s006.zip › Appendix S1, STRUCTURE and PCA Plots, Dryad/STRUCTURE/C. austriacus & C. melapterus/job_T25.pdf]

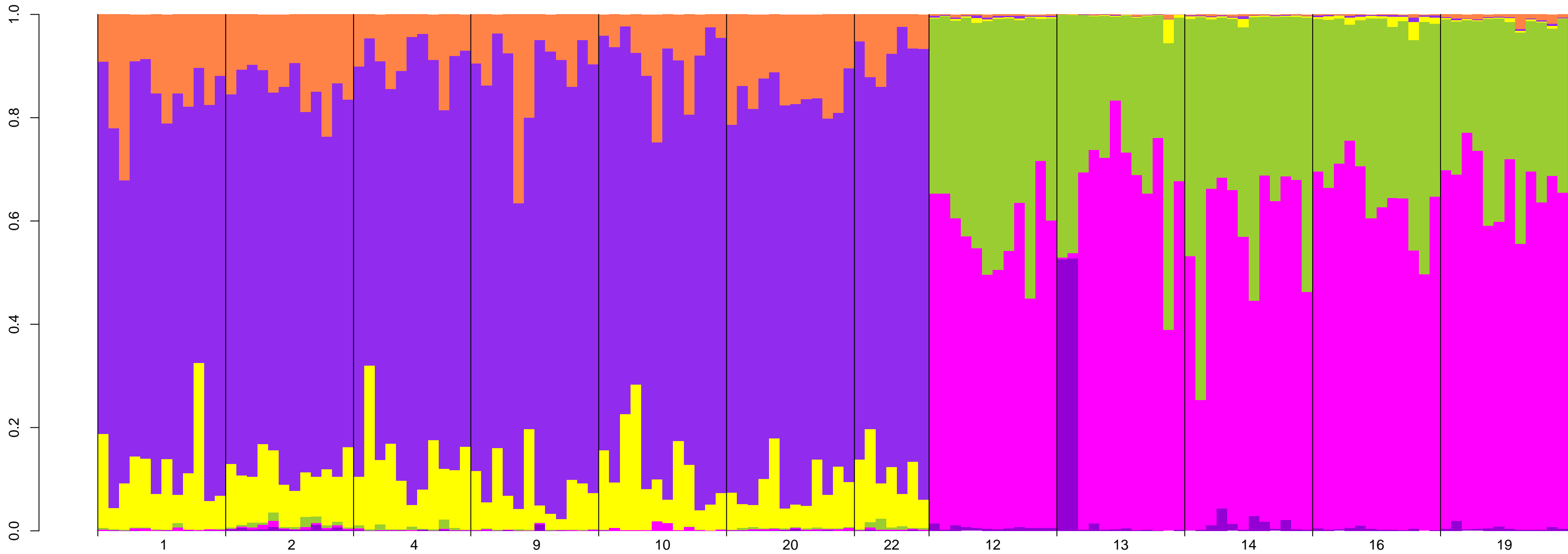

Supplement: Supplementary file 6 — Appendix S1 [file ECE3-10-4314-s006.zip › Appendix S1, STRUCTURE and PCA Plots, Dryad/STRUCTURE/C. austriacus & C. melapterus/job_T26.pdf]

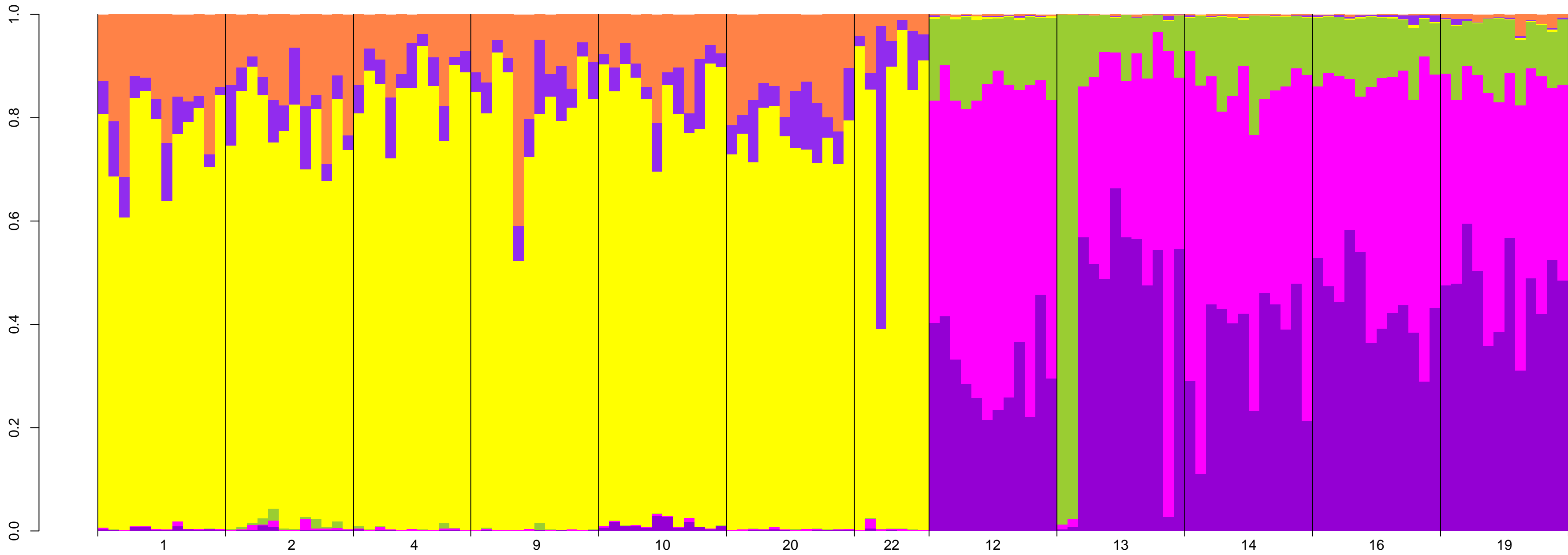

Supplement: Supplementary file 6 — Appendix S1 [file ECE3-10-4314-s006.zip › Appendix S1, STRUCTURE and PCA Plots, Dryad/STRUCTURE/C. austriacus & C. melapterus/job_T27.pdf]

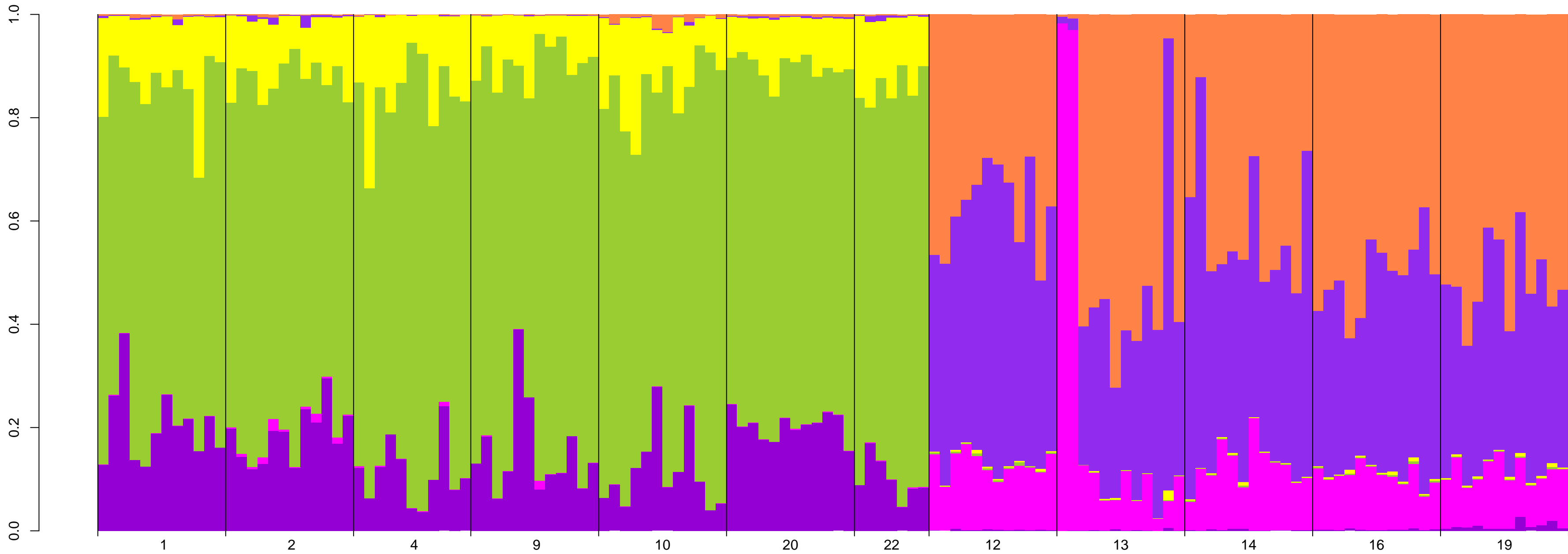

Supplement: Supplementary file 6 — Appendix S1 [file ECE3-10-4314-s006.zip › Appendix S1, STRUCTURE and PCA Plots, Dryad/STRUCTURE/C. austriacus & C. melapterus/job_T28.pdf]

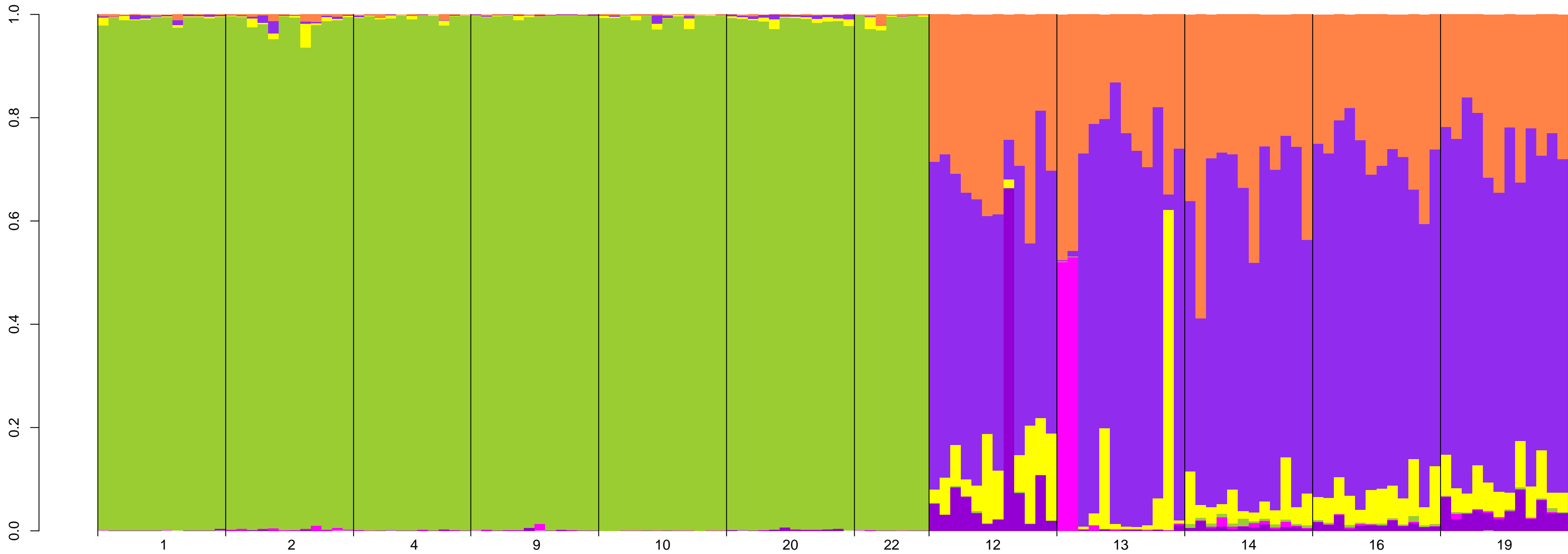

Supplement: Supplementary file 6 — Appendix S1 [file ECE3-10-4314-s006.zip › Appendix S1, STRUCTURE and PCA Plots, Dryad/STRUCTURE/C. austriacus & C. melapterus/job_T29.pdf]

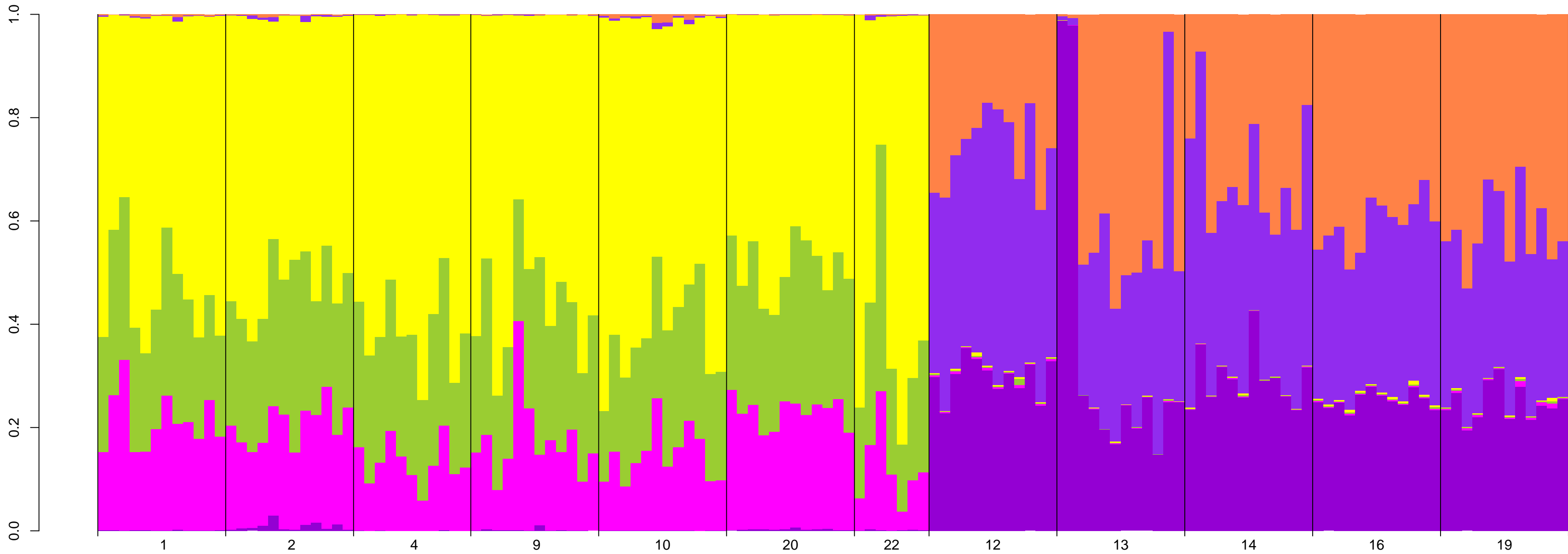

Supplement: Supplementary file 6 — Appendix S1 [file ECE3-10-4314-s006.zip › Appendix S1, STRUCTURE and PCA Plots, Dryad/STRUCTURE/C. austriacus & C. melapterus/job_T30.pdf]

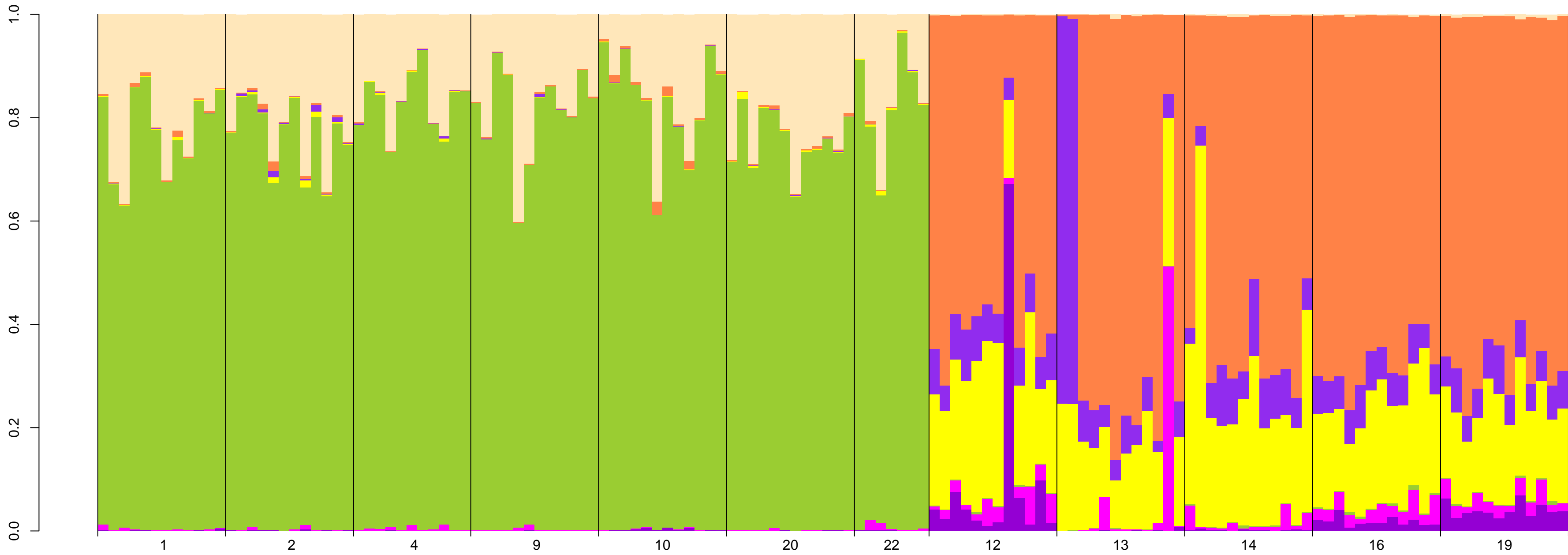

Supplement: Supplementary file 6 — Appendix S1 [file ECE3-10-4314-s006.zip › Appendix S1, STRUCTURE and PCA Plots, Dryad/STRUCTURE/C. austriacus & C. melapterus/job_T31.pdf]

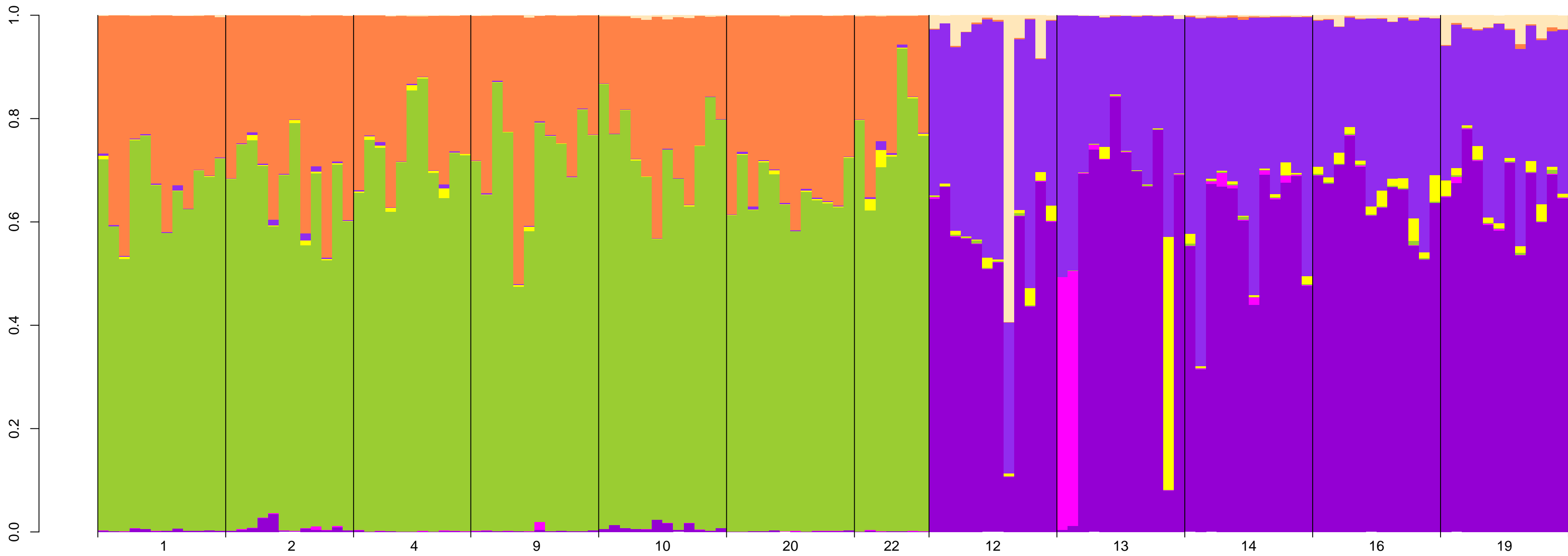

Supplement: Supplementary file 6 — Appendix S1 [file ECE3-10-4314-s006.zip › Appendix S1, STRUCTURE and PCA Plots, Dryad/STRUCTURE/C. austriacus & C. melapterus/job_T32.pdf]

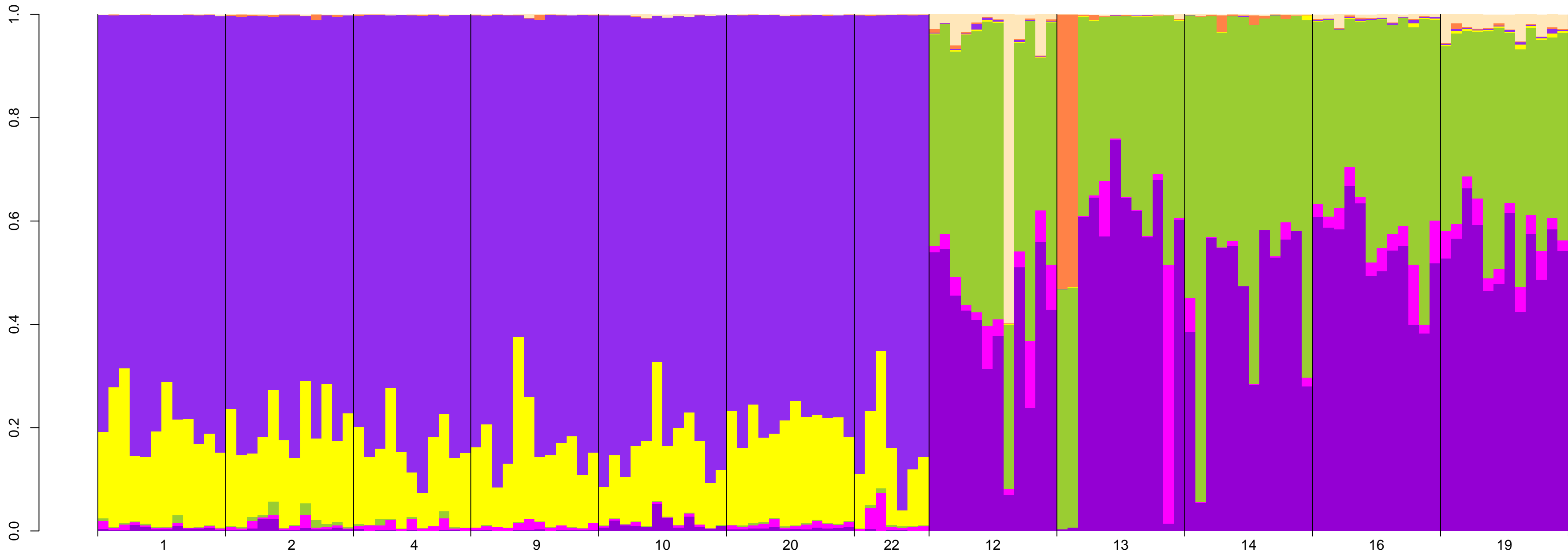

Supplement: Supplementary file 6 — Appendix S1 [file ECE3-10-4314-s006.zip › Appendix S1, STRUCTURE and PCA Plots, Dryad/STRUCTURE/C. austriacus & C. melapterus/job_T33.pdf]

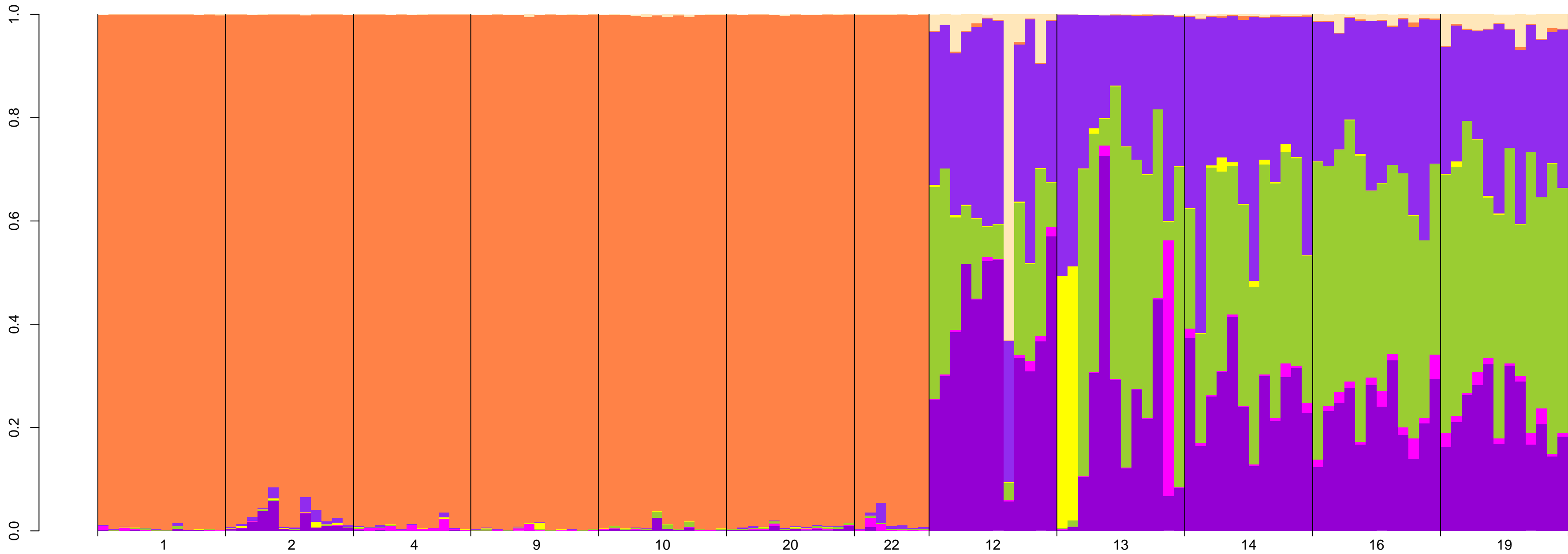

Supplement: Supplementary file 6 — Appendix S1 [file ECE3-10-4314-s006.zip › Appendix S1, STRUCTURE and PCA Plots, Dryad/STRUCTURE/C. austriacus & C. melapterus/job_T34.pdf]

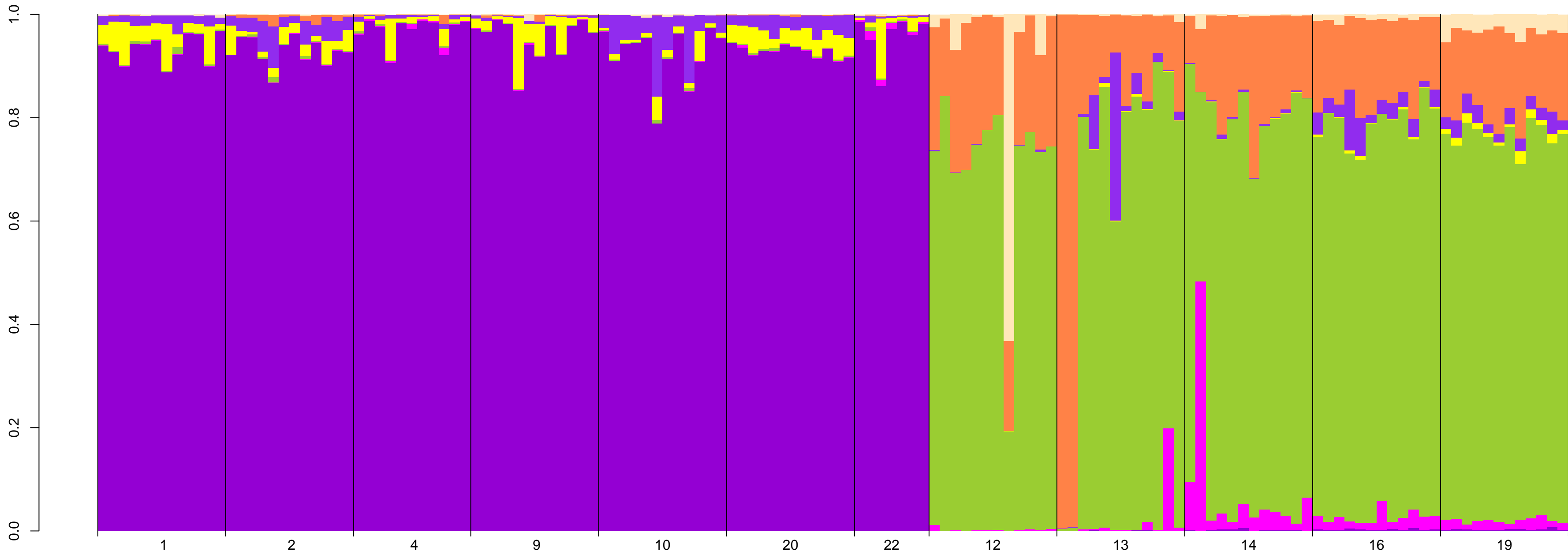

Supplement: Supplementary file 6 — Appendix S1 [file ECE3-10-4314-s006.zip › Appendix S1, STRUCTURE and PCA Plots, Dryad/STRUCTURE/C. austriacus & C. melapterus/job_T35.pdf]

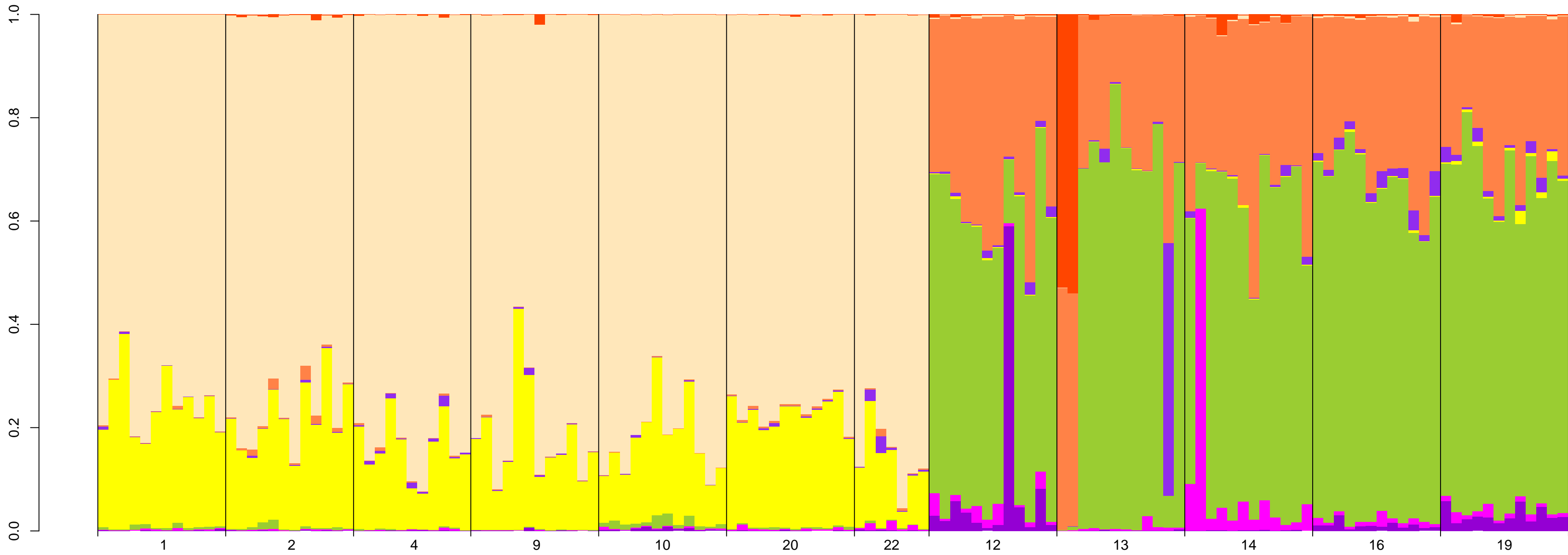

Supplement: Supplementary file 6 — Appendix S1 [file ECE3-10-4314-s006.zip › Appendix S1, STRUCTURE and PCA Plots, Dryad/STRUCTURE/C. austriacus & C. melapterus/job_T36.pdf]

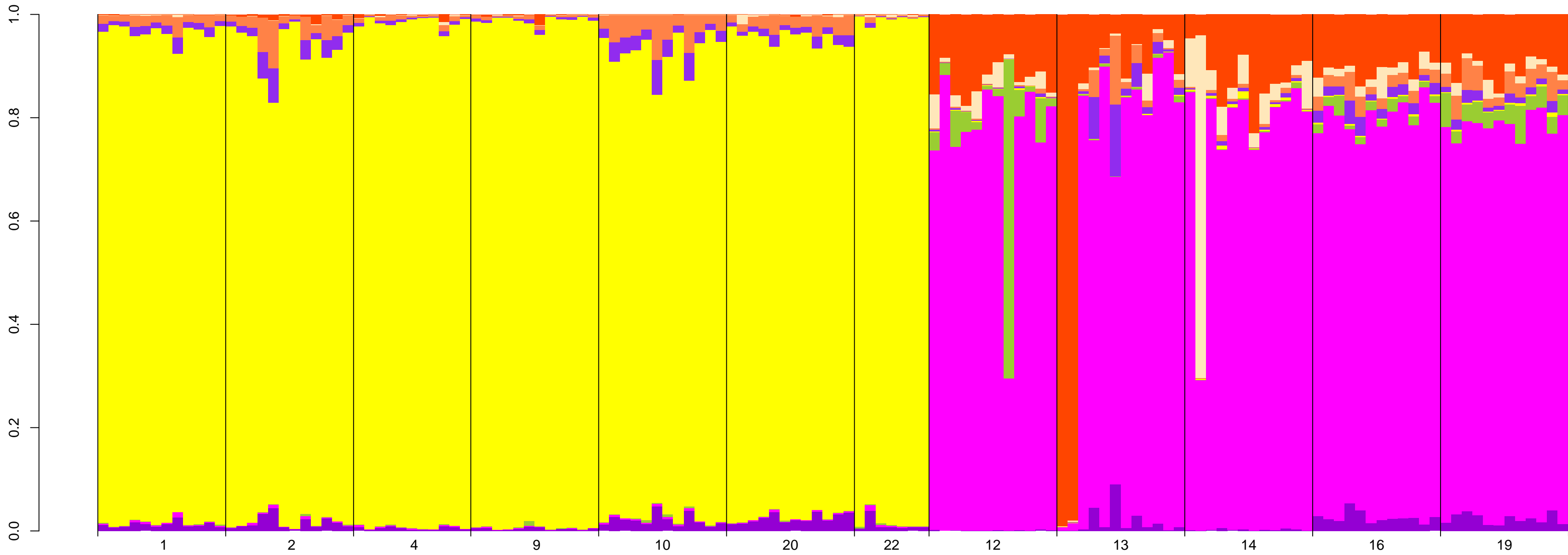

Supplement: Supplementary file 6 — Appendix S1 [file ECE3-10-4314-s006.zip › Appendix S1, STRUCTURE and PCA Plots, Dryad/STRUCTURE/C. austriacus & C. melapterus/job_T37.pdf]

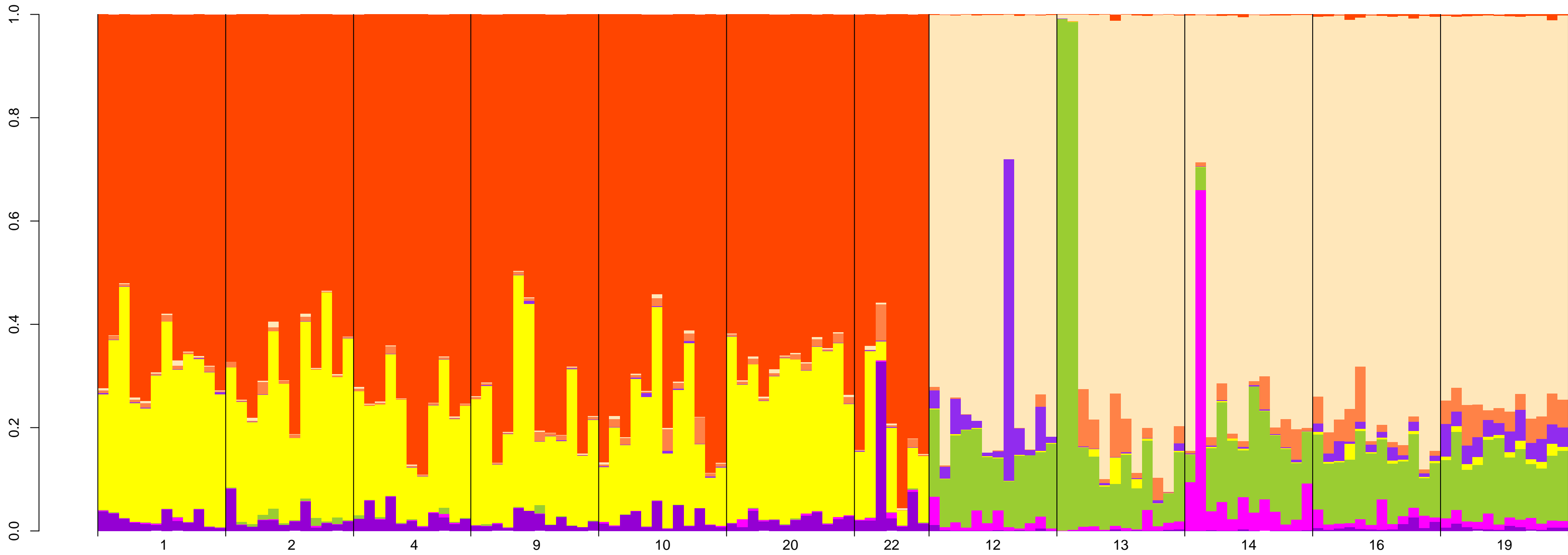

Supplement: Supplementary file 6 — Appendix S1 [file ECE3-10-4314-s006.zip › Appendix S1, STRUCTURE and PCA Plots, Dryad/STRUCTURE/C. austriacus & C. melapterus/job_T38.pdf]

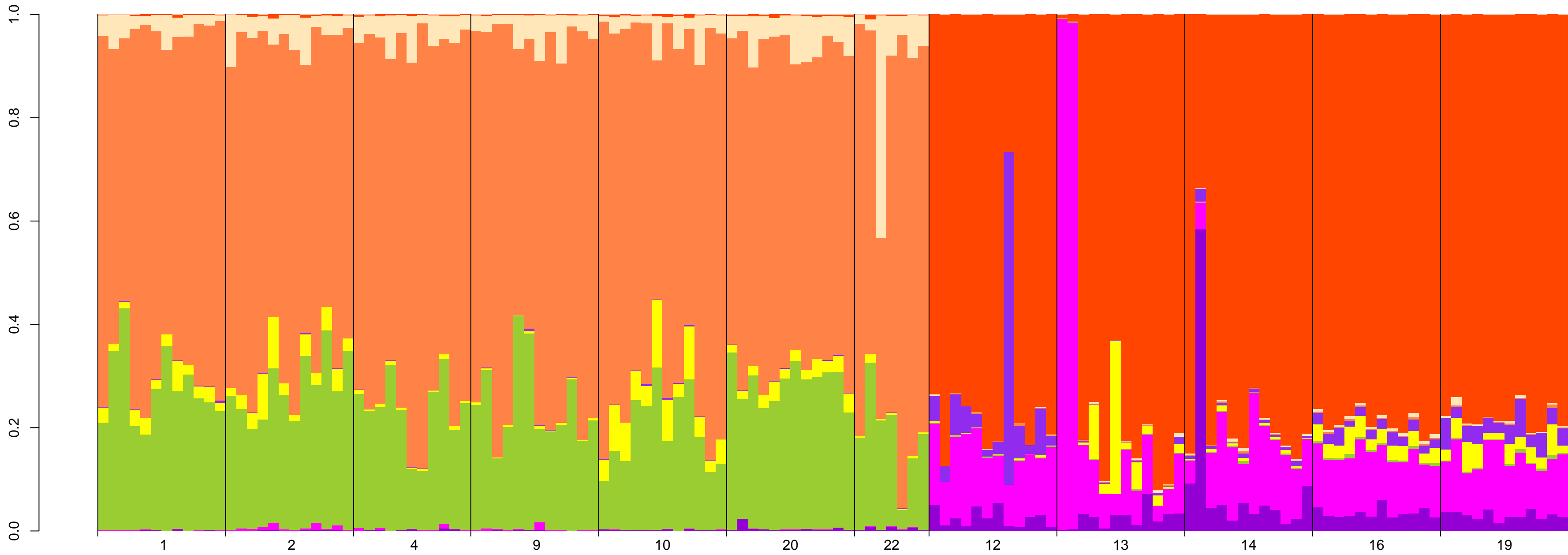

Supplement: Supplementary file 6 — Appendix S1 [file ECE3-10-4314-s006.zip › Appendix S1, STRUCTURE and PCA Plots, Dryad/STRUCTURE/C. austriacus & C. melapterus/job_T39.pdf]

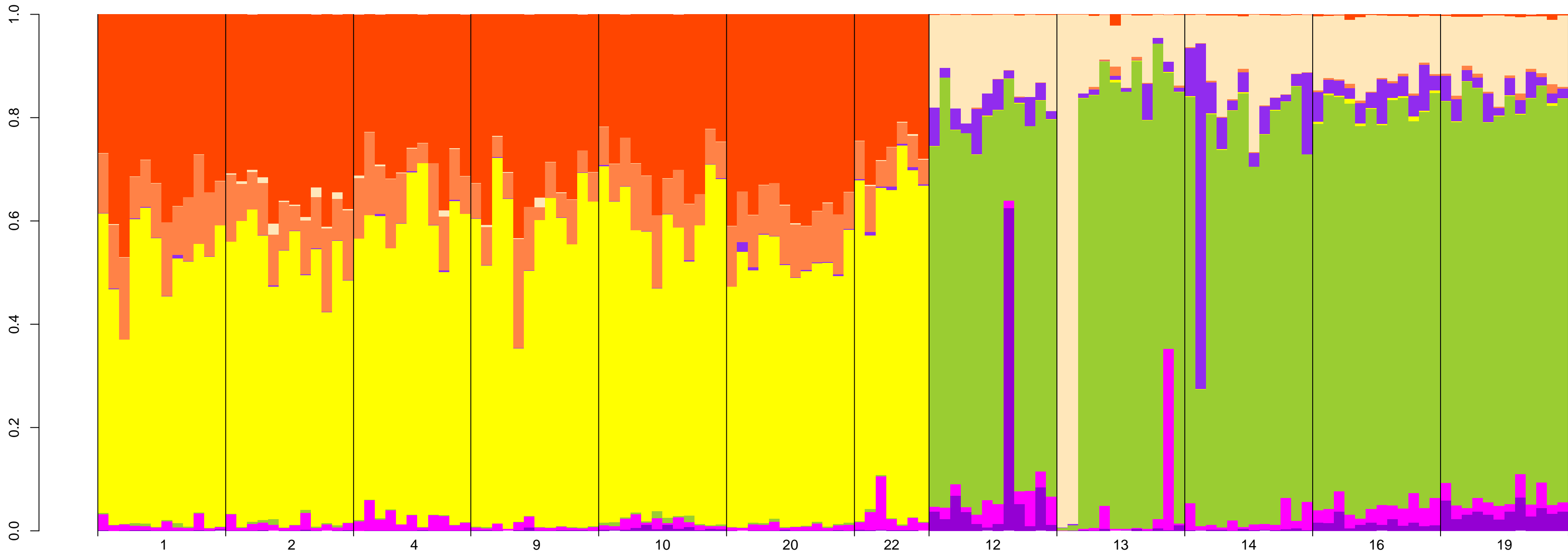

Supplement: Supplementary file 6 — Appendix S1 [file ECE3-10-4314-s006.zip › Appendix S1, STRUCTURE and PCA Plots, Dryad/STRUCTURE/C. austriacus & C. melapterus/job_T40.pdf]

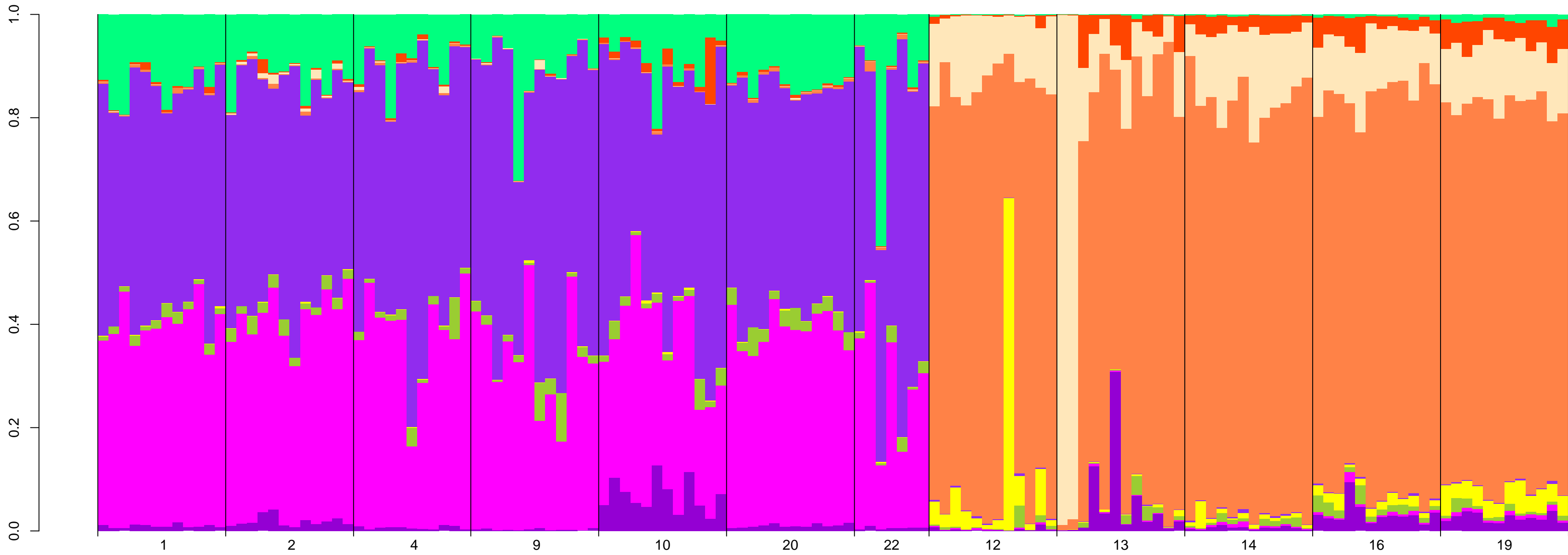

Supplement: Supplementary file 6 — Appendix S1 [file ECE3-10-4314-s006.zip › Appendix S1, STRUCTURE and PCA Plots, Dryad/STRUCTURE/C. austriacus & C. melapterus/job_T41.pdf]

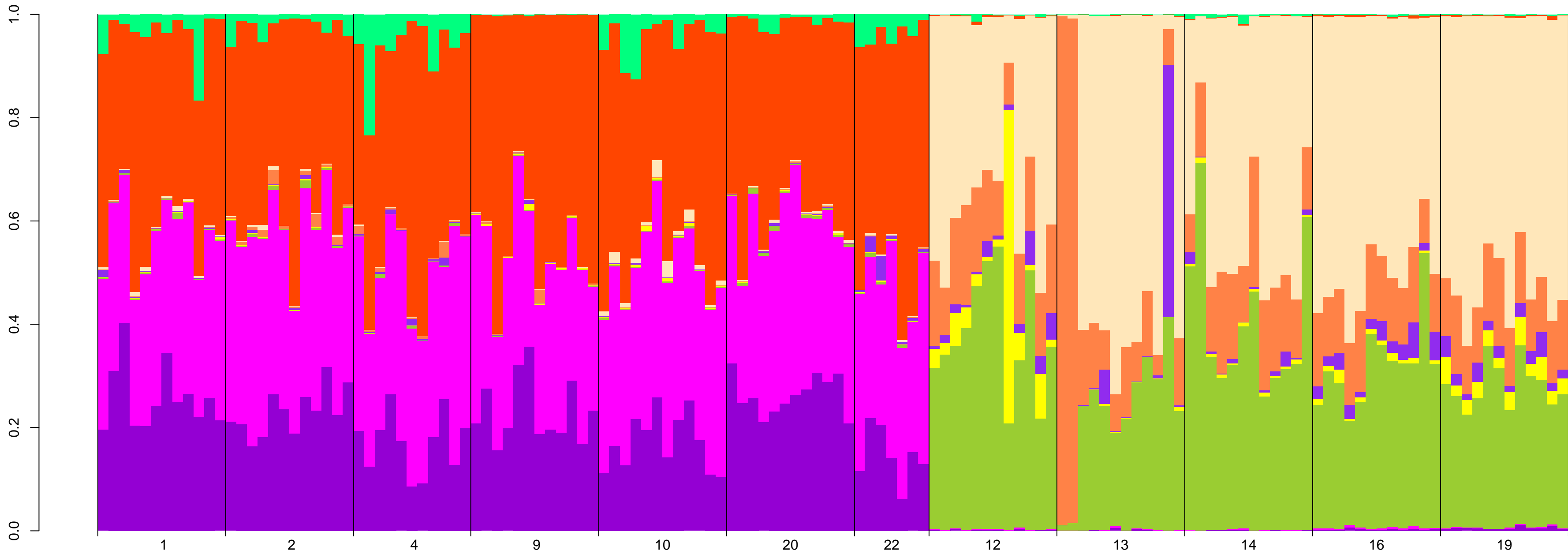

Supplement: Supplementary file 6 — Appendix S1 [file ECE3-10-4314-s006.zip › Appendix S1, STRUCTURE and PCA Plots, Dryad/STRUCTURE/C. austriacus & C. melapterus/job_T42.pdf]

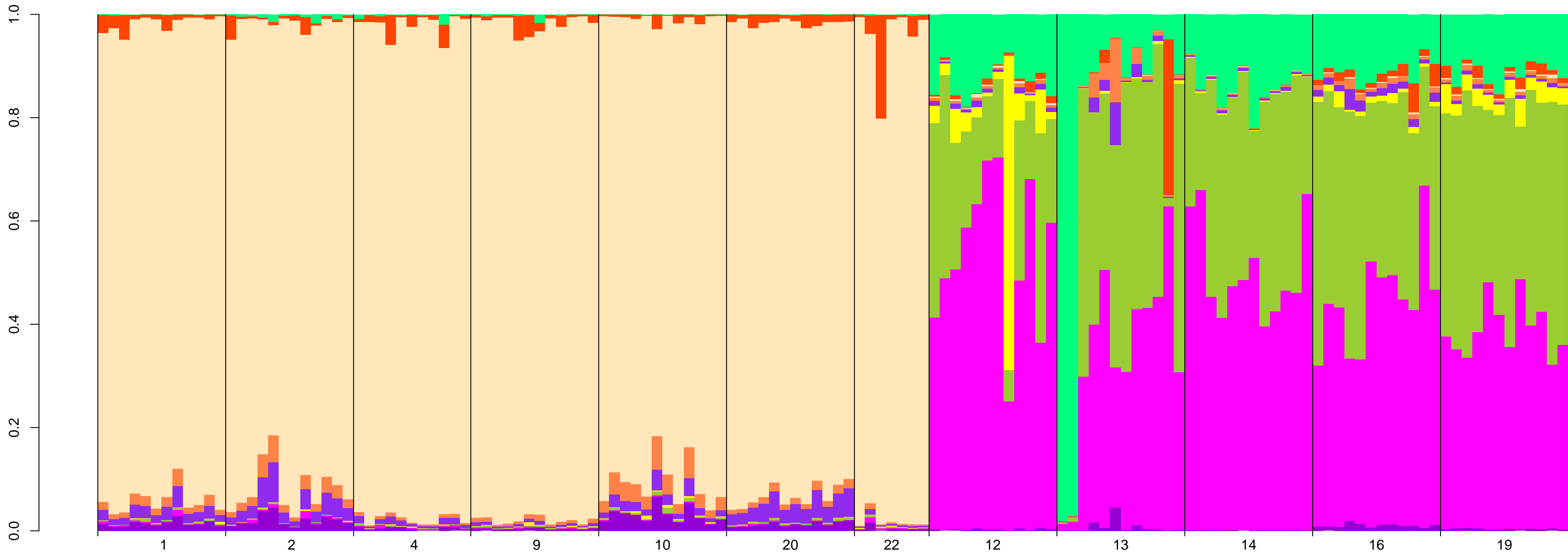

Supplement: Supplementary file 6 — Appendix S1 [file ECE3-10-4314-s006.zip › Appendix S1, STRUCTURE and PCA Plots, Dryad/STRUCTURE/C. austriacus & C. melapterus/job_T43.pdf]

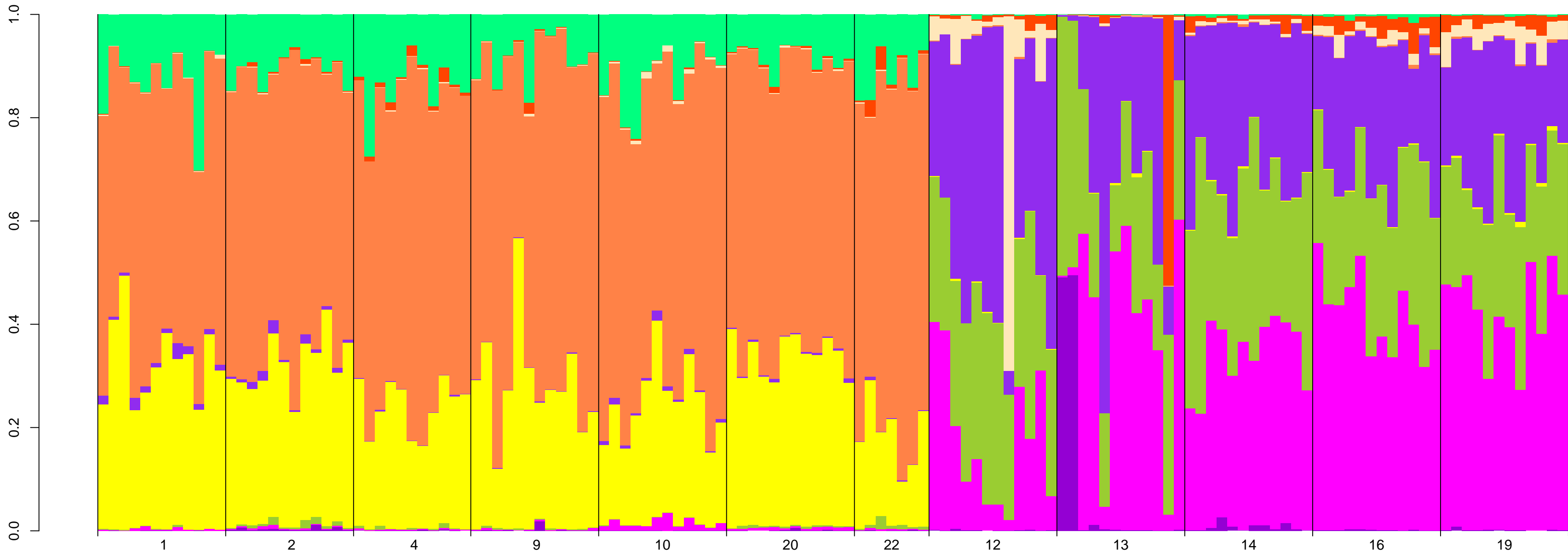

Supplement: Supplementary file 6 — Appendix S1 [file ECE3-10-4314-s006.zip › Appendix S1, STRUCTURE and PCA Plots, Dryad/STRUCTURE/C. austriacus & C. melapterus/job_T44.pdf]

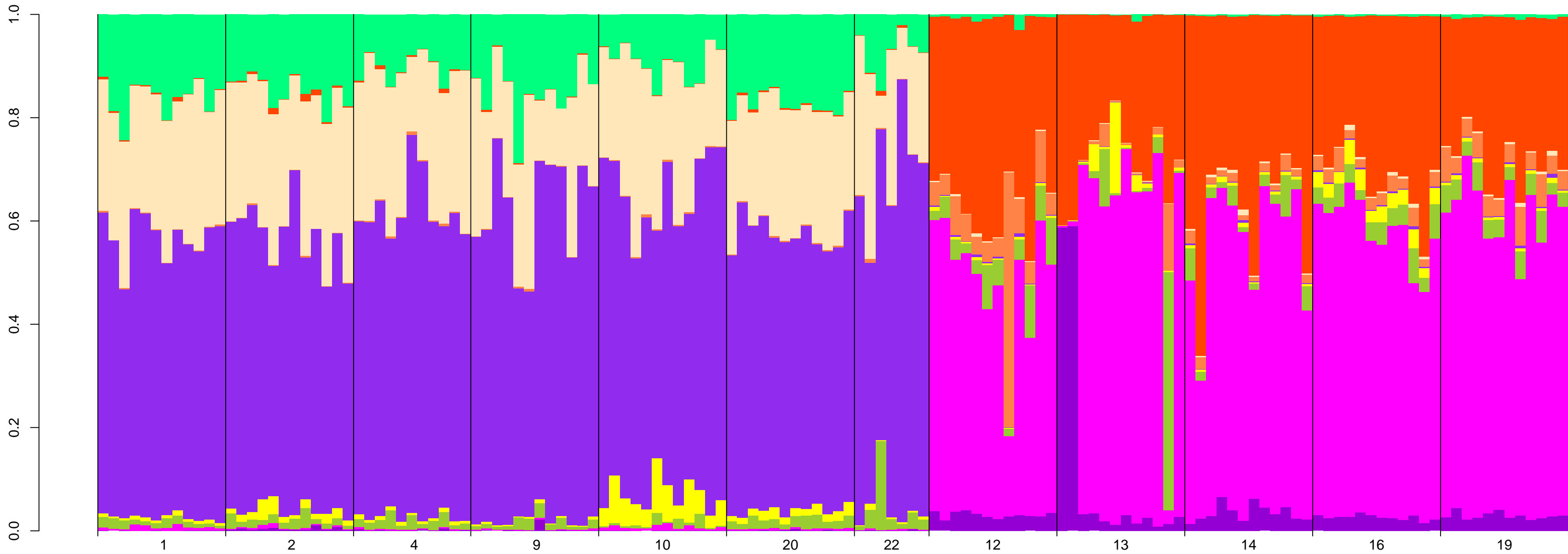

Supplement: Supplementary file 6 — Appendix S1 [file ECE3-10-4314-s006.zip › Appendix S1, STRUCTURE and PCA Plots, Dryad/STRUCTURE/C. austriacus & C. melapterus/job_T45.pdf]

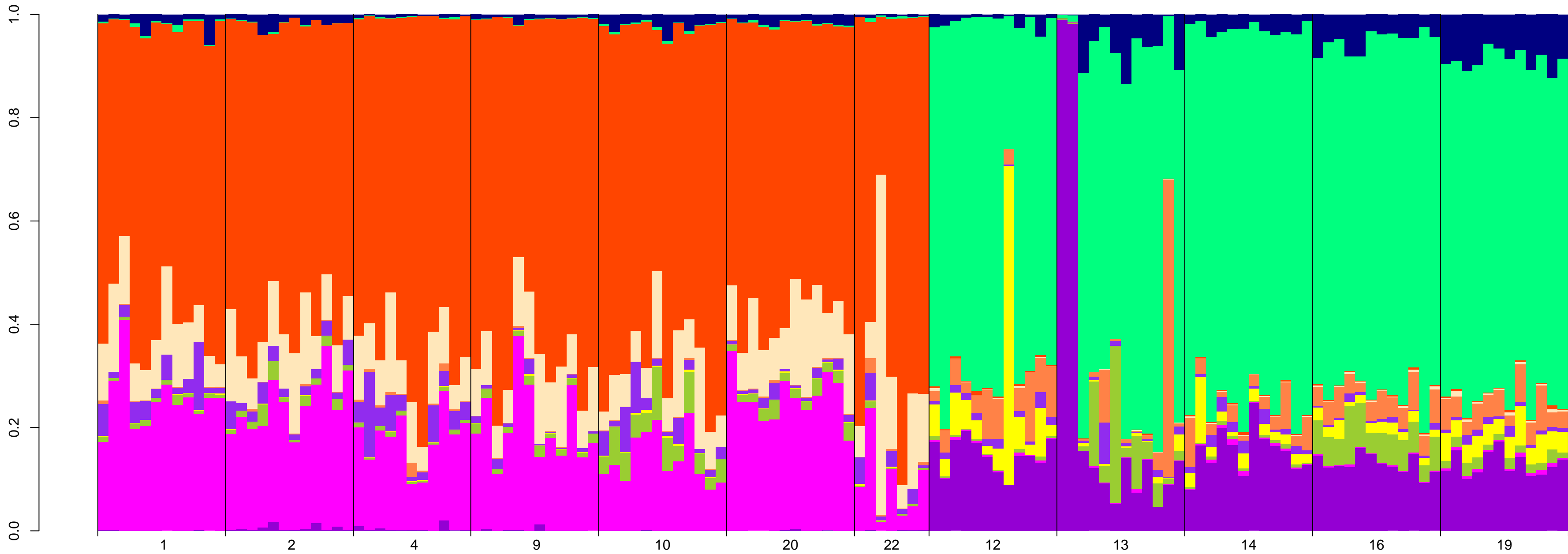

Supplement: Supplementary file 6 — Appendix S1 [file ECE3-10-4314-s006.zip › Appendix S1, STRUCTURE and PCA Plots, Dryad/STRUCTURE/C. austriacus & C. melapterus/job_T46.pdf]

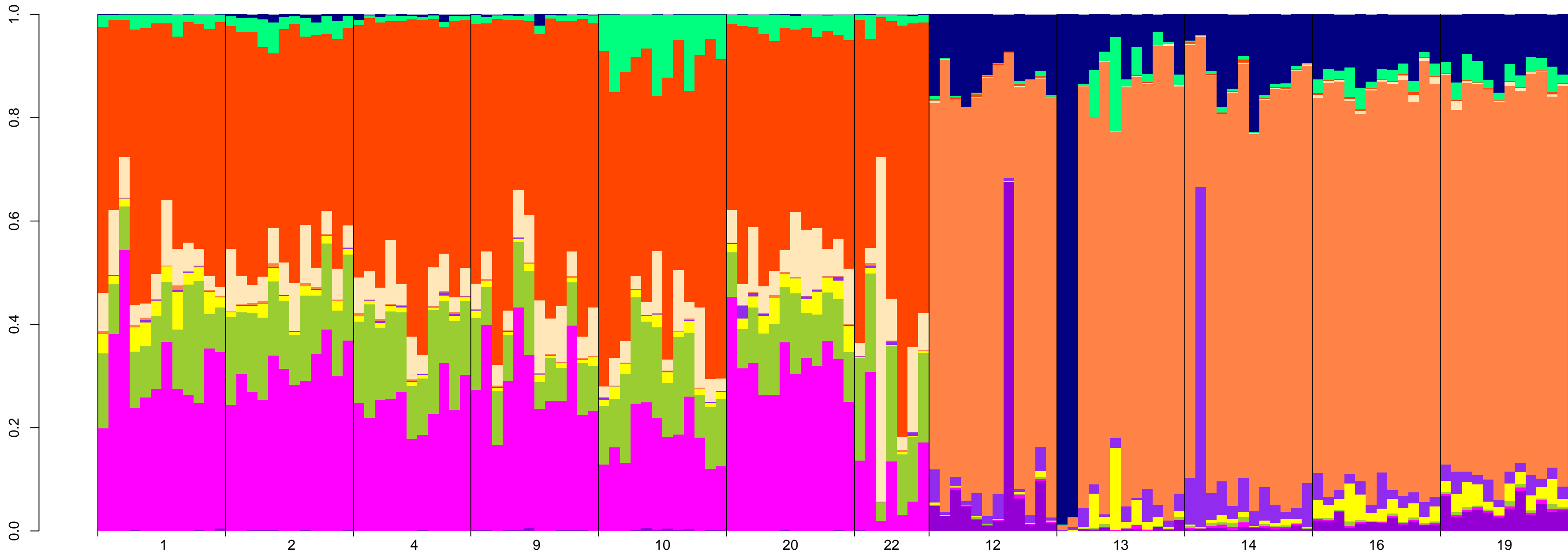

Supplement: Supplementary file 6 — Appendix S1 [file ECE3-10-4314-s006.zip › Appendix S1, STRUCTURE and PCA Plots, Dryad/STRUCTURE/C. austriacus & C. melapterus/job_T47.pdf]

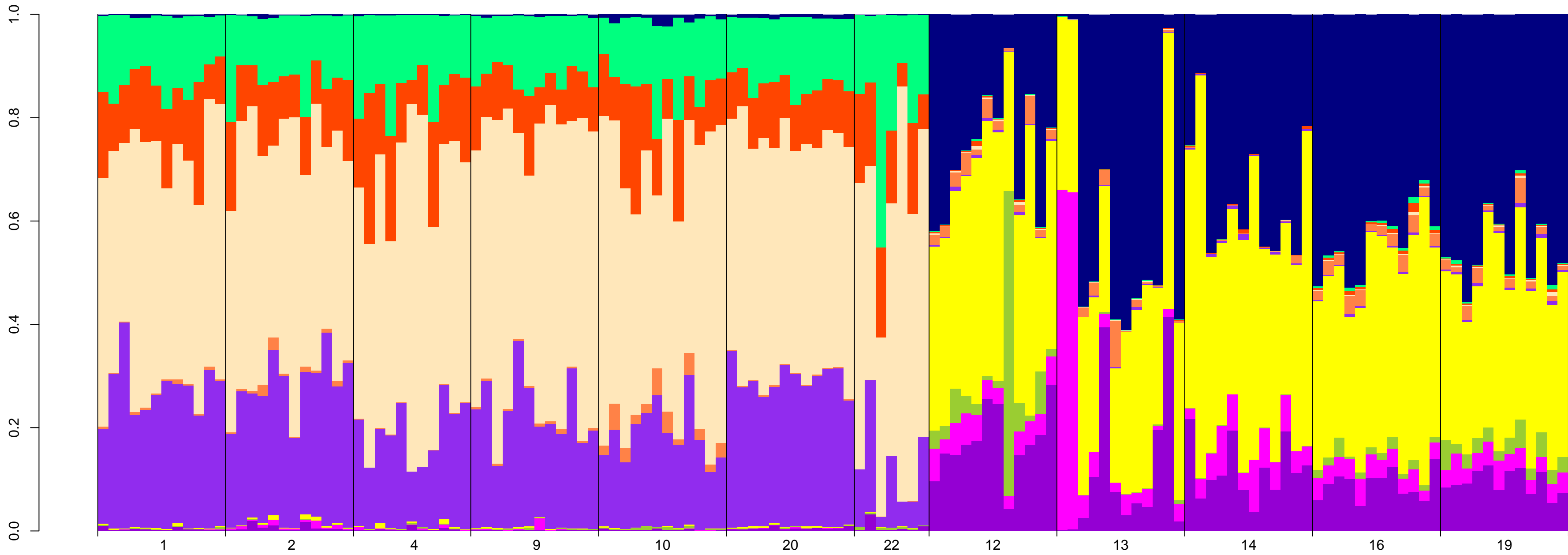

Supplement: Supplementary file 6 — Appendix S1 [file ECE3-10-4314-s006.zip › Appendix S1, STRUCTURE and PCA Plots, Dryad/STRUCTURE/C. austriacus & C. melapterus/job_T48.pdf]

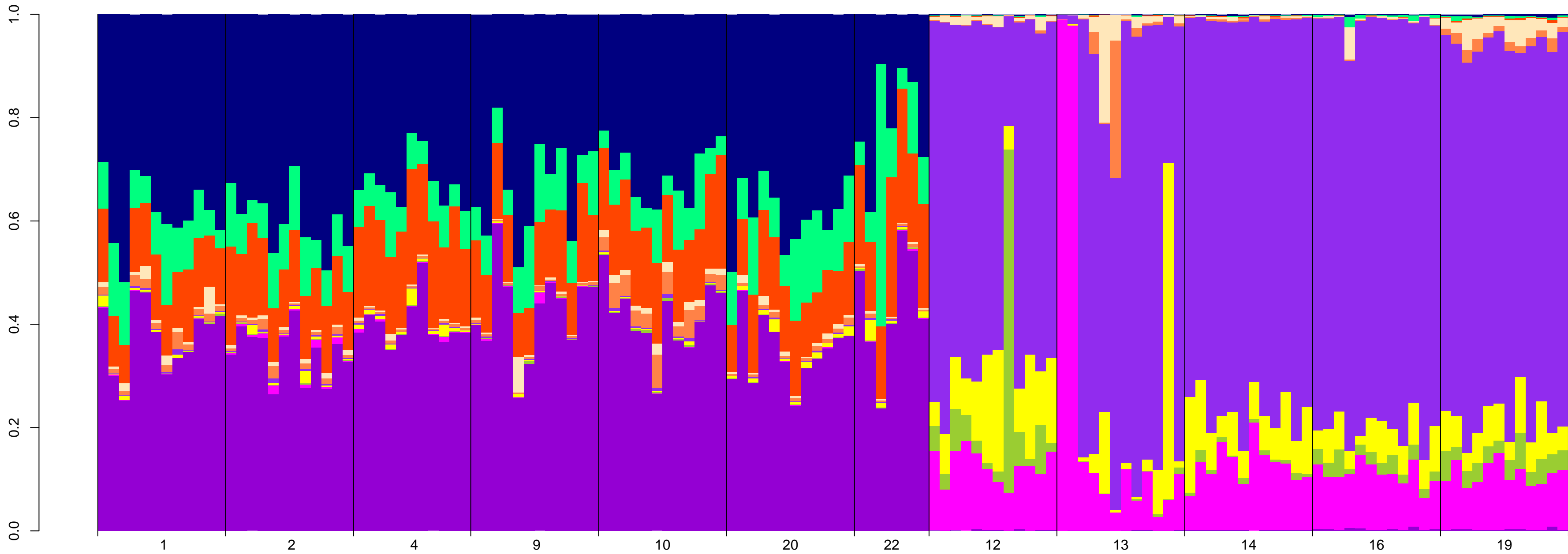

Supplement: Supplementary file 6 — Appendix S1 [file ECE3-10-4314-s006.zip › Appendix S1, STRUCTURE and PCA Plots, Dryad/STRUCTURE/C. austriacus & C. melapterus/job_T49.pdf]

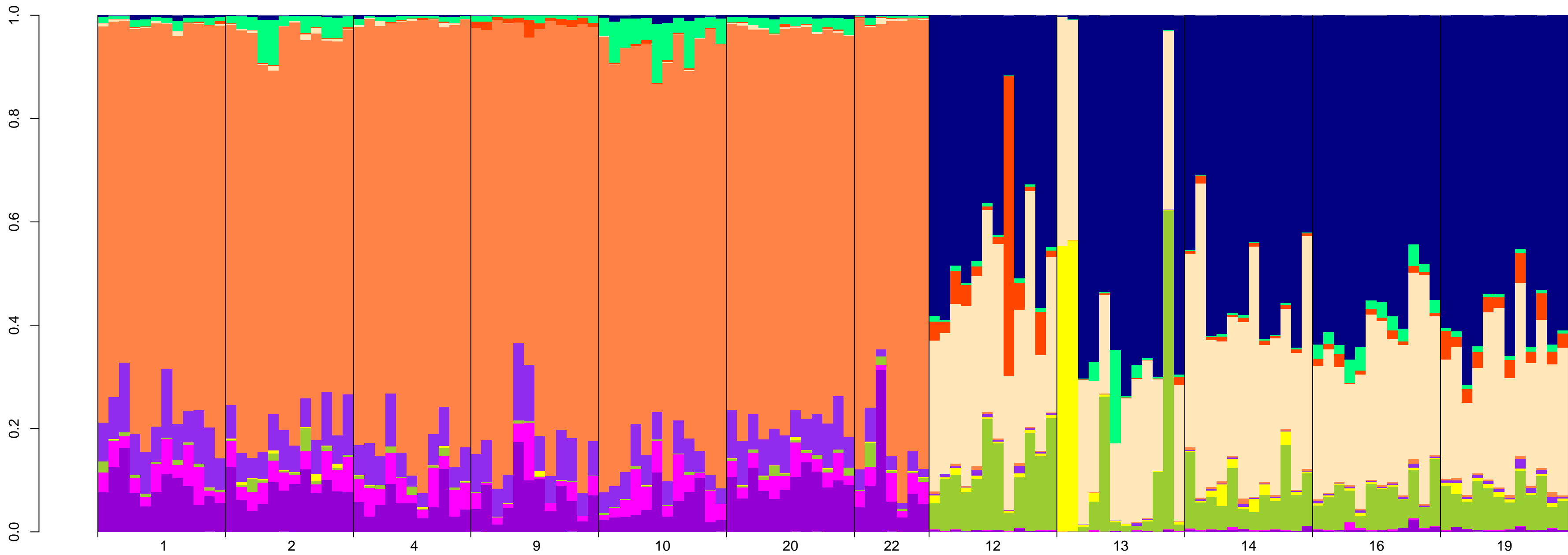

Supplement: Supplementary file 6 — Appendix S1 [file ECE3-10-4314-s006.zip › Appendix S1, STRUCTURE and PCA Plots, Dryad/STRUCTURE/C. austriacus & C. melapterus/job_T50.pdf]

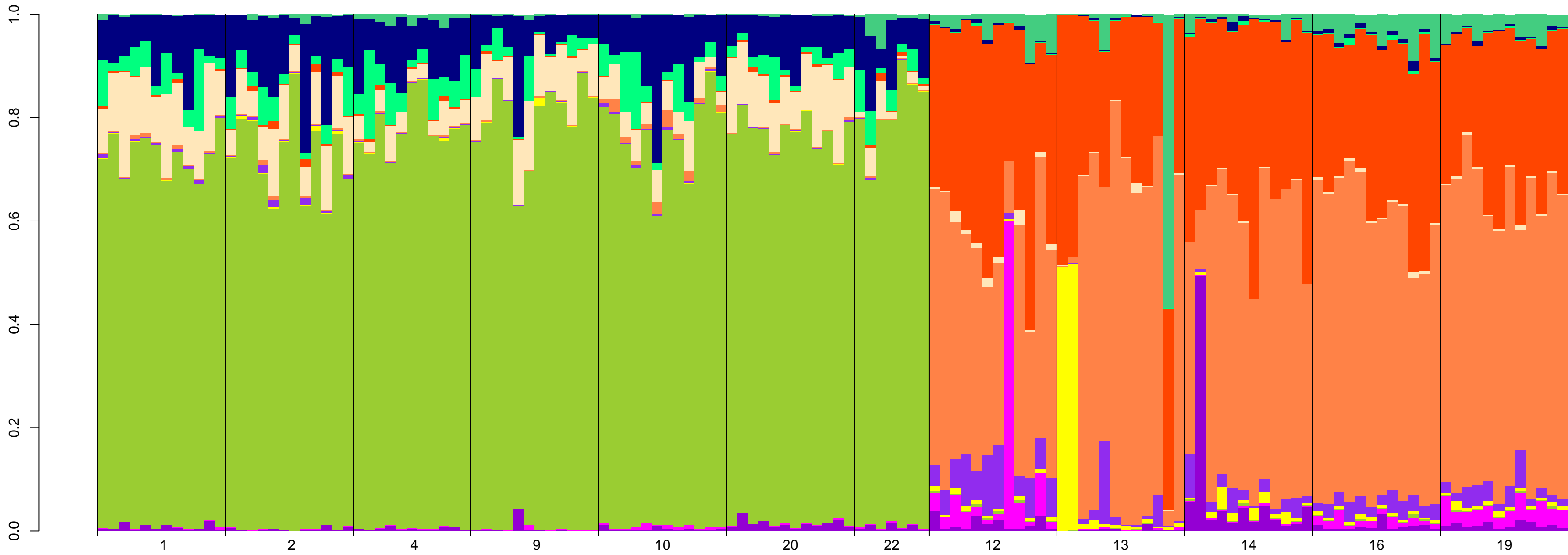

Supplement: Supplementary file 6 — Appendix S1 [file ECE3-10-4314-s006.zip › Appendix S1, STRUCTURE and PCA Plots, Dryad/STRUCTURE/C. austriacus & C. melapterus/job_T51.pdf]

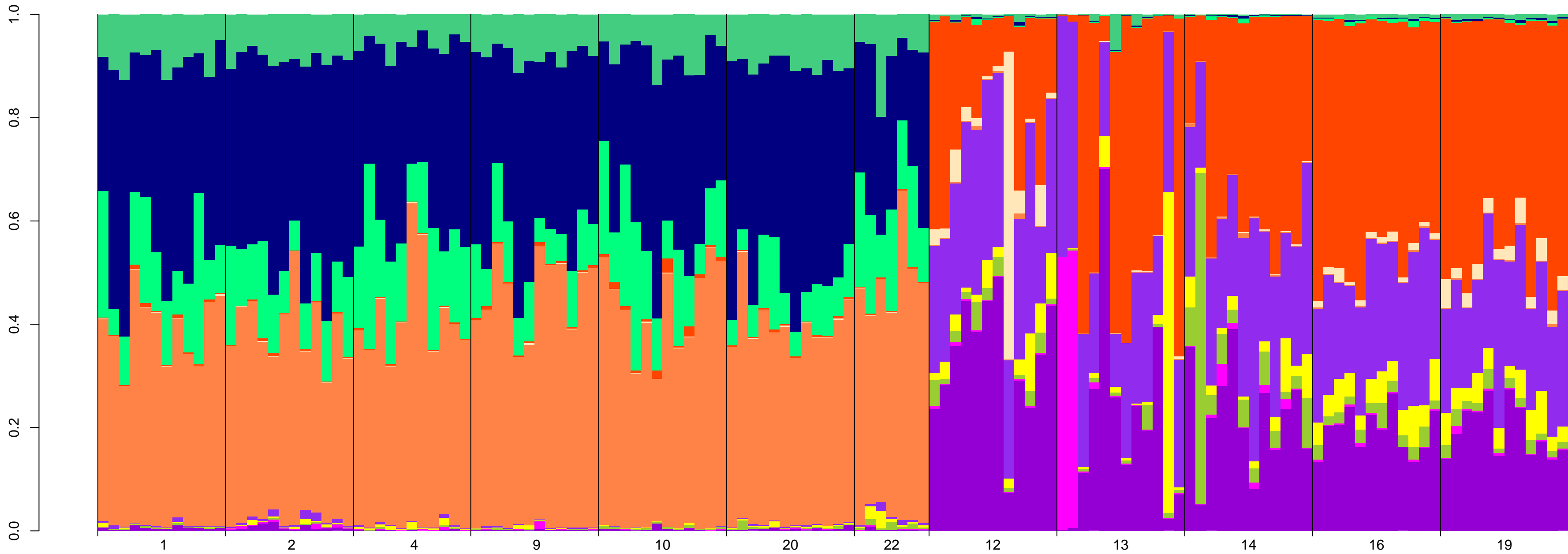

Supplement: Supplementary file 6 — Appendix S1 [file ECE3-10-4314-s006.zip › Appendix S1, STRUCTURE and PCA Plots, Dryad/STRUCTURE/C. austriacus & C. melapterus/job_T52.pdf]

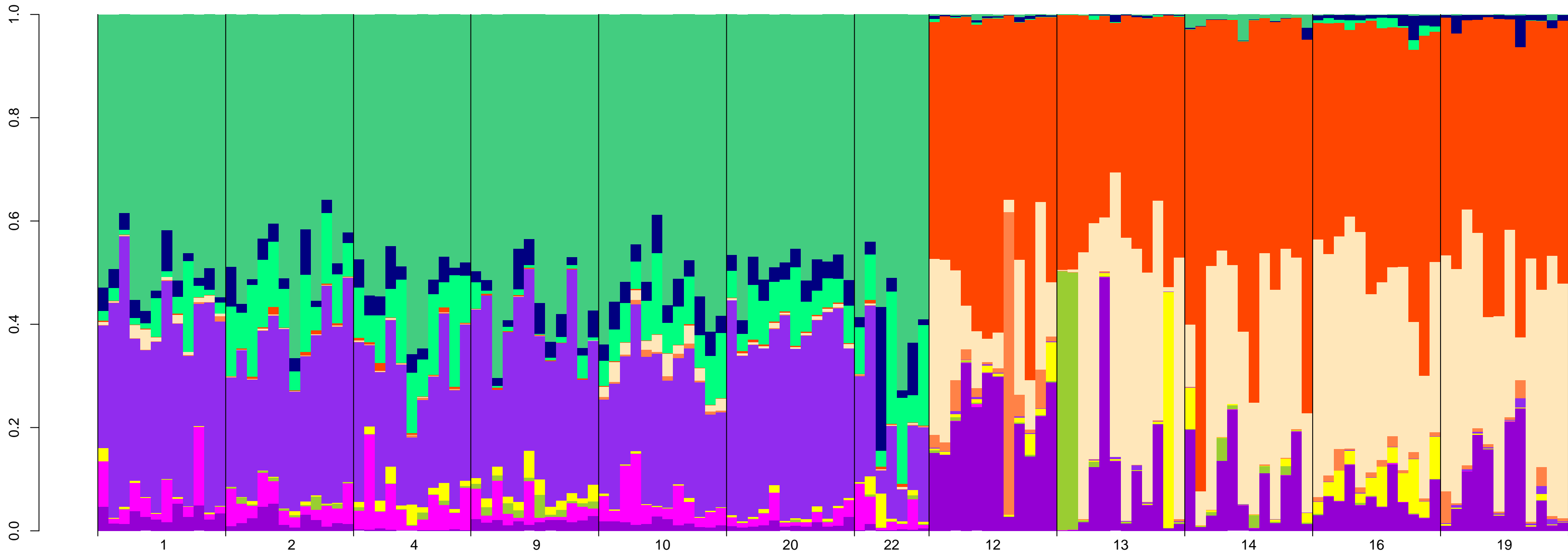

Supplement: Supplementary file 6 — Appendix S1 [file ECE3-10-4314-s006.zip › Appendix S1, STRUCTURE and PCA Plots, Dryad/STRUCTURE/C. austriacus & C. melapterus/job_T53.pdf]

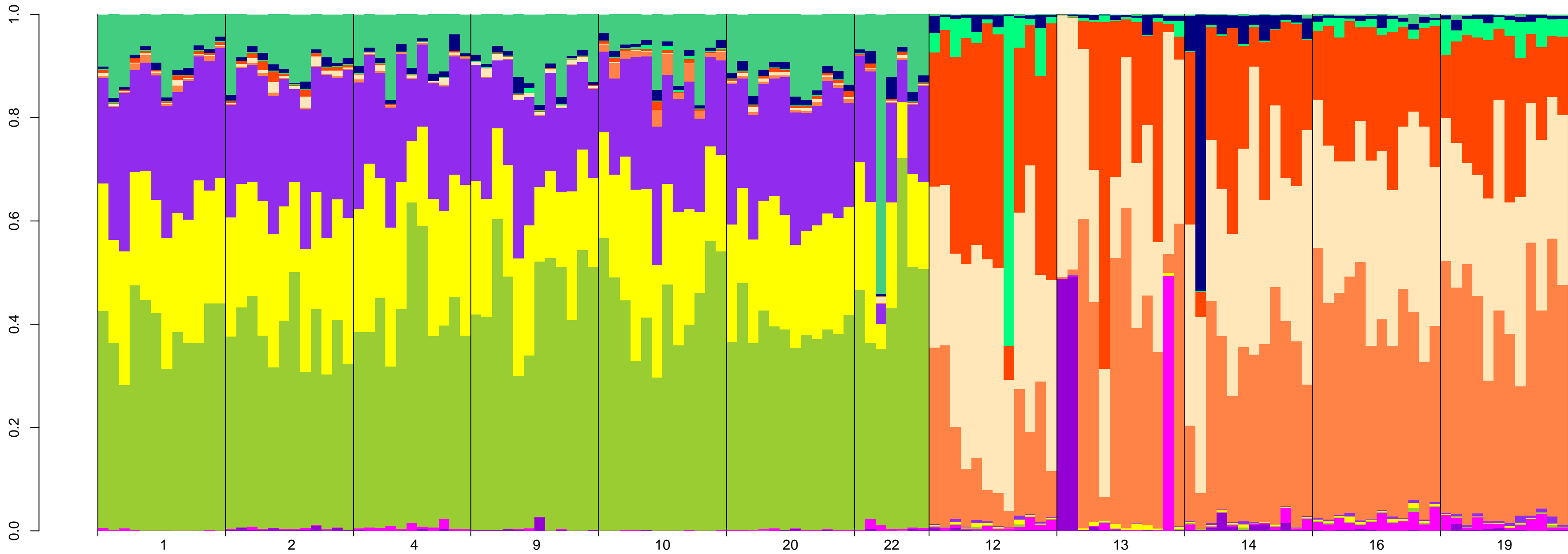

Supplement: Supplementary file 6 — Appendix S1 [file ECE3-10-4314-s006.zip › Appendix S1, STRUCTURE and PCA Plots, Dryad/STRUCTURE/C. austriacus & C. melapterus/job_T54.pdf]

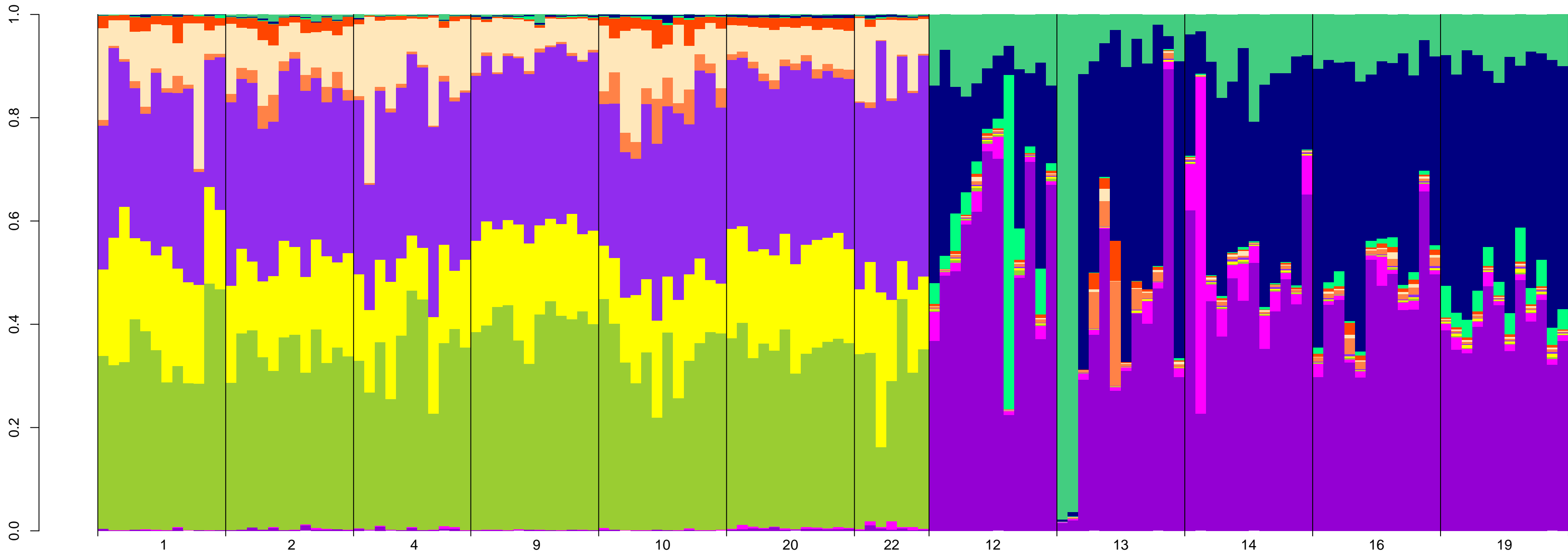

Supplement: Supplementary file 6 — Appendix S1 [file ECE3-10-4314-s006.zip › Appendix S1, STRUCTURE and PCA Plots, Dryad/STRUCTURE/C. austriacus & C. melapterus/job_T55.pdf]

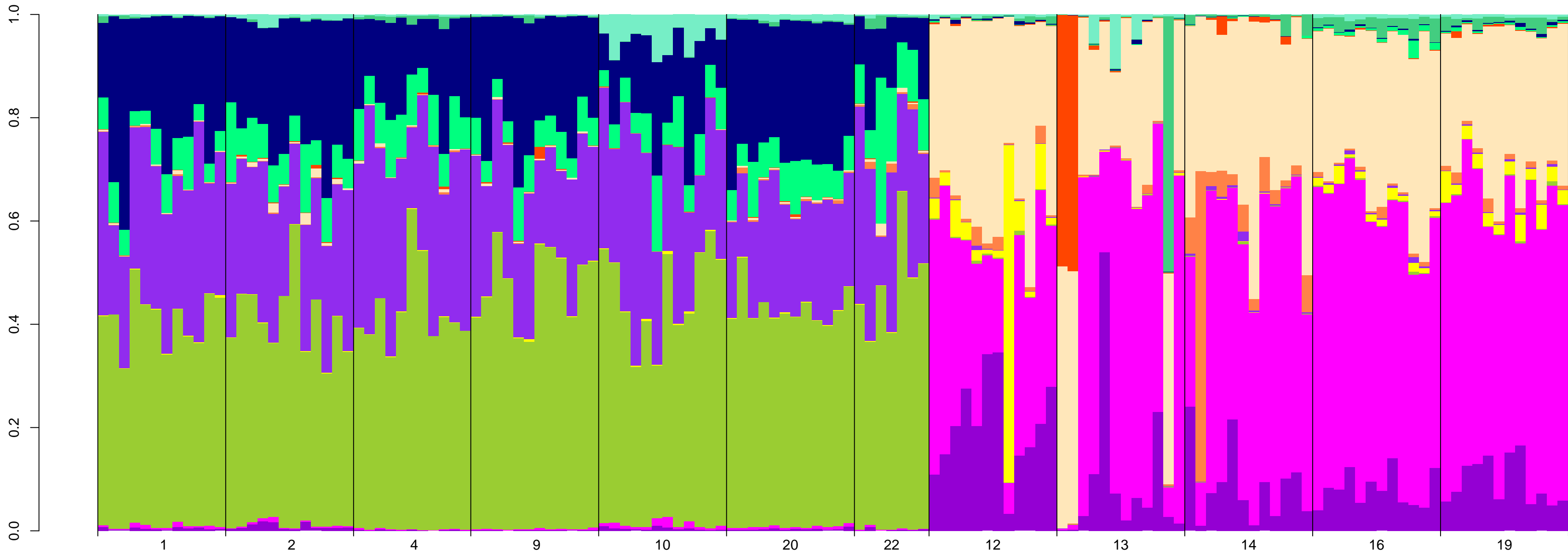

Supplement: Supplementary file 6 — Appendix S1 [file ECE3-10-4314-s006.zip › Appendix S1, STRUCTURE and PCA Plots, Dryad/STRUCTURE/C. austriacus & C. melapterus/job_T56.pdf]

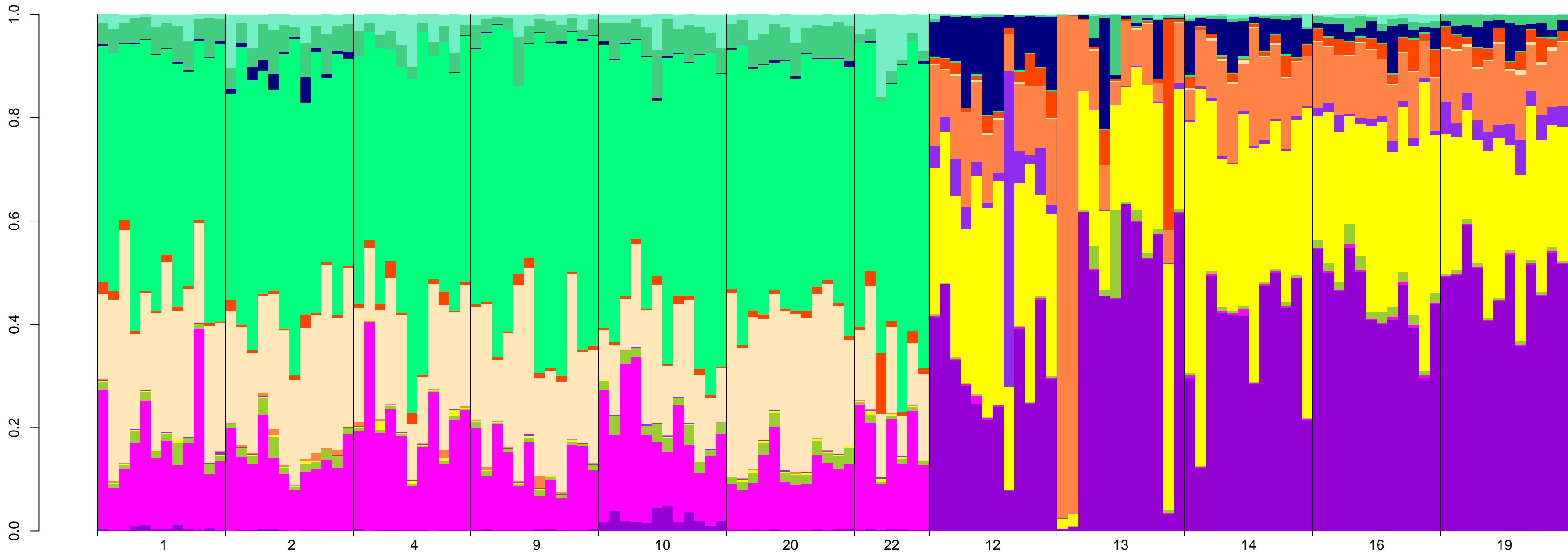

Supplement: Supplementary file 6 — Appendix S1 [file ECE3-10-4314-s006.zip › Appendix S1, STRUCTURE and PCA Plots, Dryad/STRUCTURE/C. austriacus & C. melapterus/job_T57.pdf]

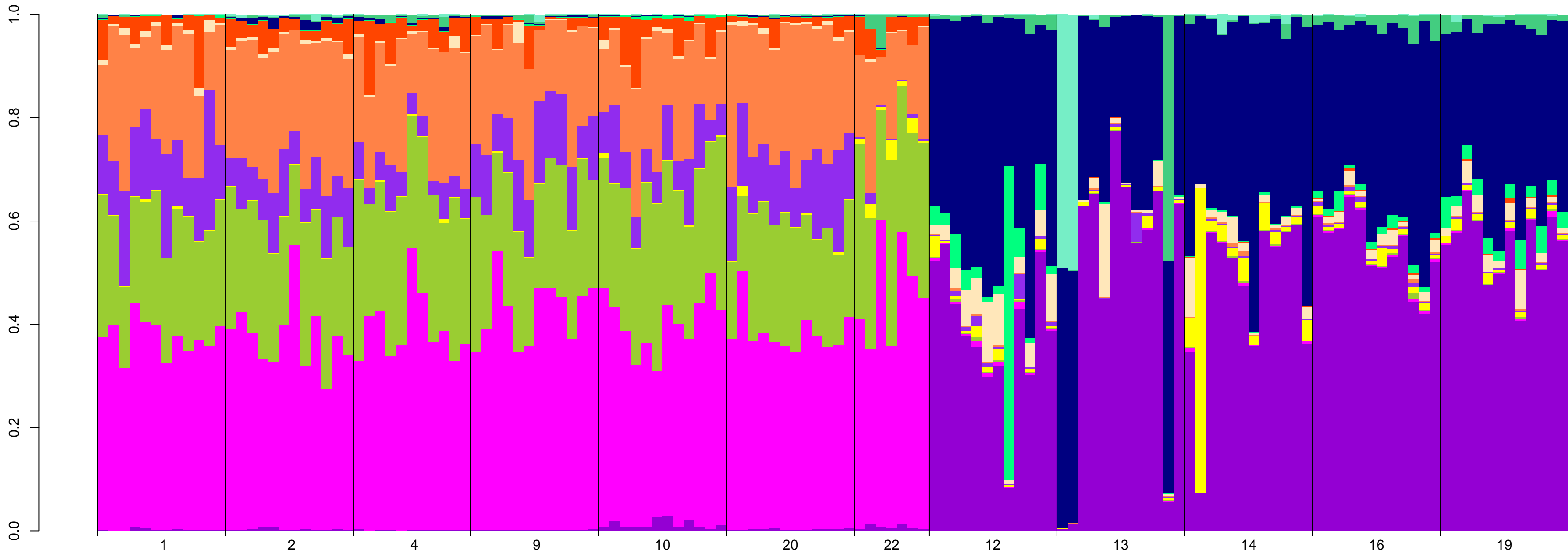

Supplement: Supplementary file 6 — Appendix S1 [file ECE3-10-4314-s006.zip › Appendix S1, STRUCTURE and PCA Plots, Dryad/STRUCTURE/C. austriacus & C. melapterus/job_T58.pdf]

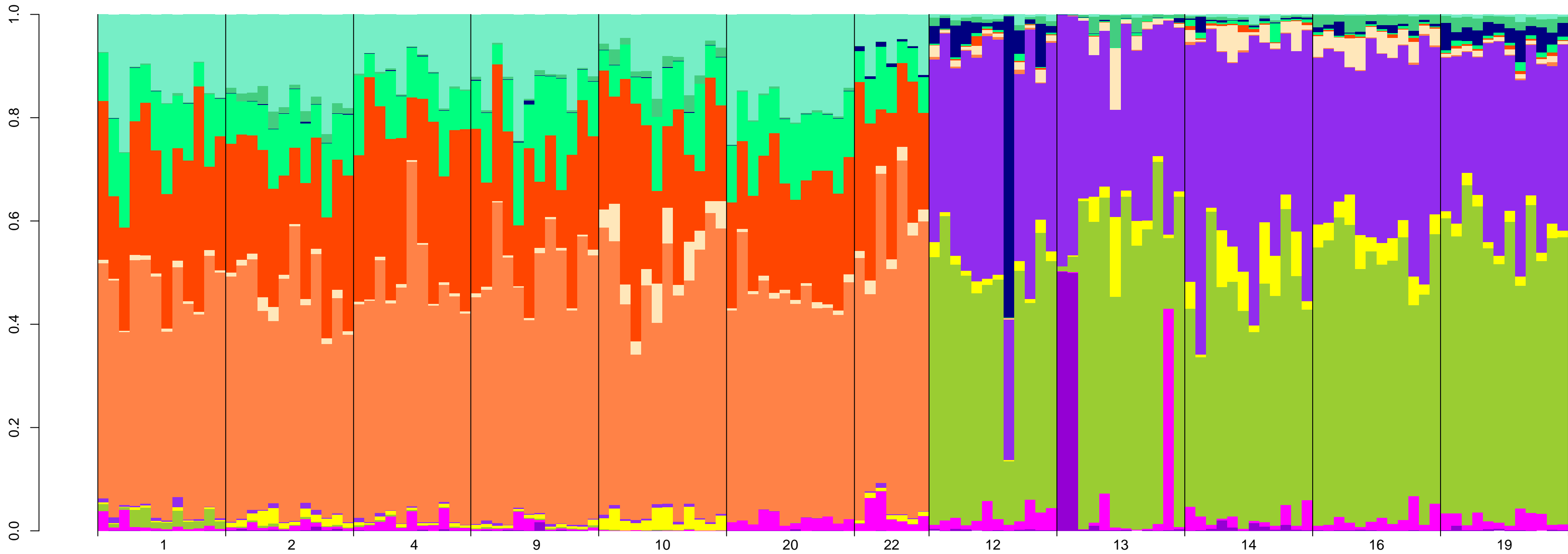

Supplement: Supplementary file 6 — Appendix S1 [file ECE3-10-4314-s006.zip › Appendix S1, STRUCTURE and PCA Plots, Dryad/STRUCTURE/C. austriacus & C. melapterus/job_T59.pdf]

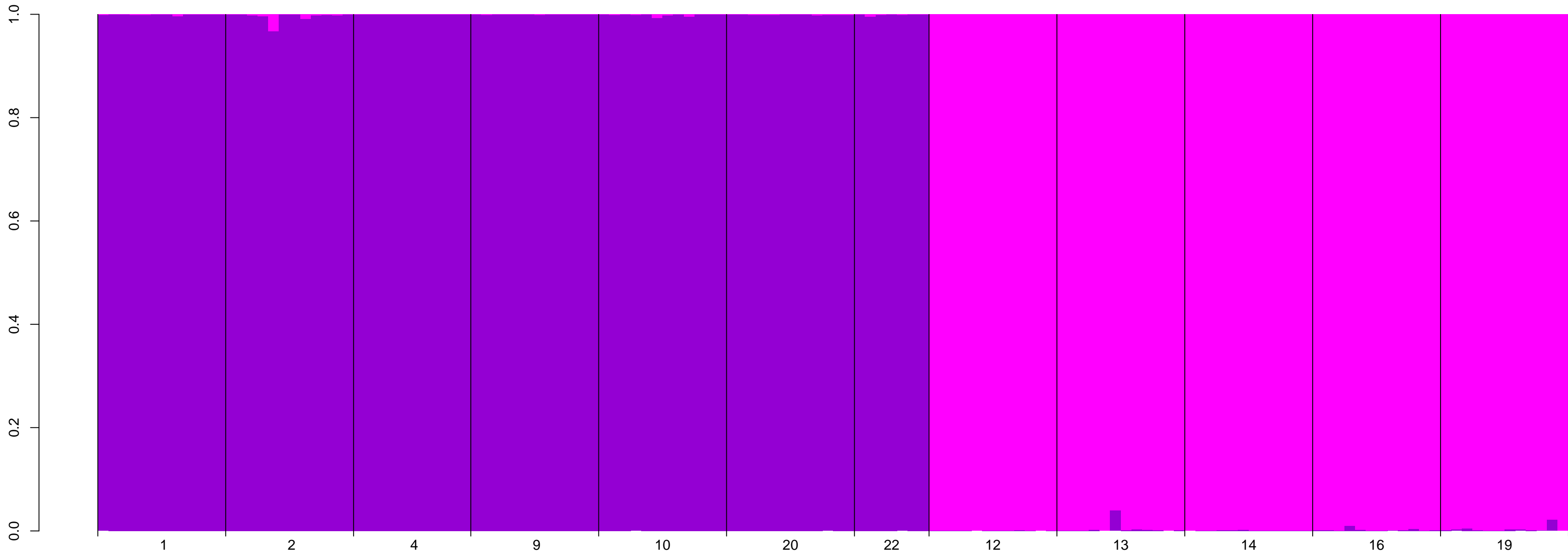

Supplement: Supplementary file 6 — Appendix S1 [file ECE3-10-4314-s006.zip › Appendix S1, STRUCTURE and PCA Plots, Dryad/STRUCTURE/C. austriacus & C. melapterus/job_T6.pdf]

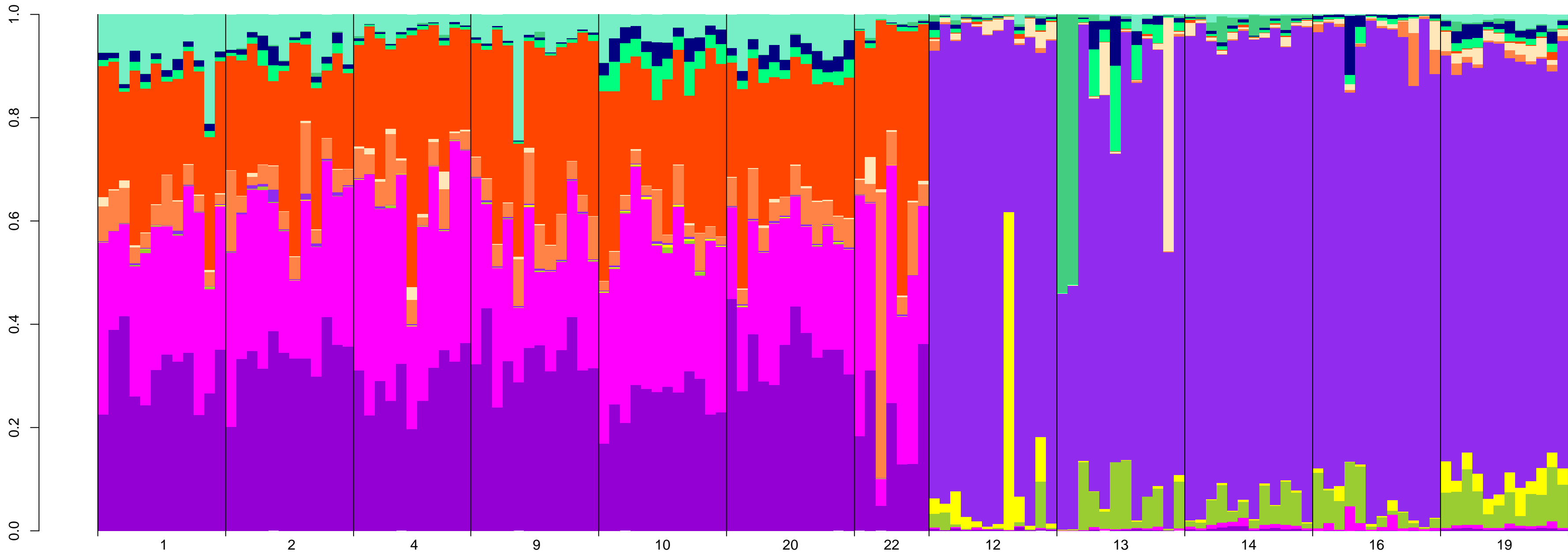

Supplement: Supplementary file 6 — Appendix S1 [file ECE3-10-4314-s006.zip › Appendix S1, STRUCTURE and PCA Plots, Dryad/STRUCTURE/C. austriacus & C. melapterus/job_T60.pdf]

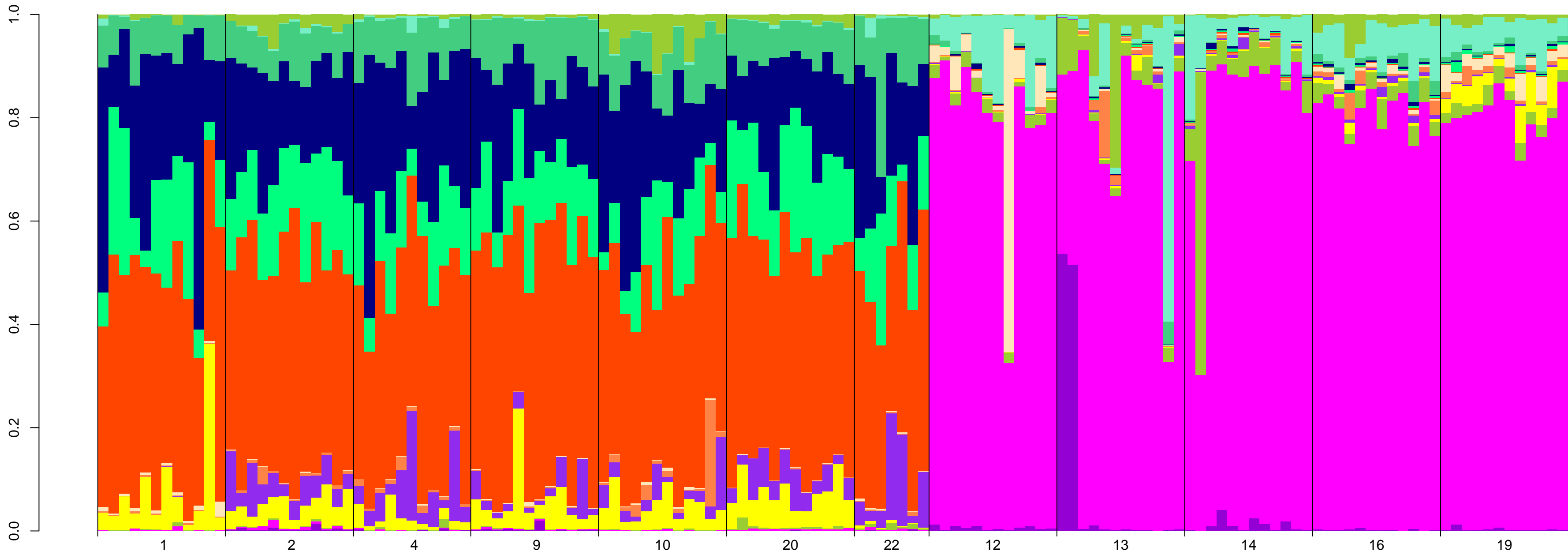

Supplement: Supplementary file 6 — Appendix S1 [file ECE3-10-4314-s006.zip › Appendix S1, STRUCTURE and PCA Plots, Dryad/STRUCTURE/C. austriacus & C. melapterus/job_T61.pdf]

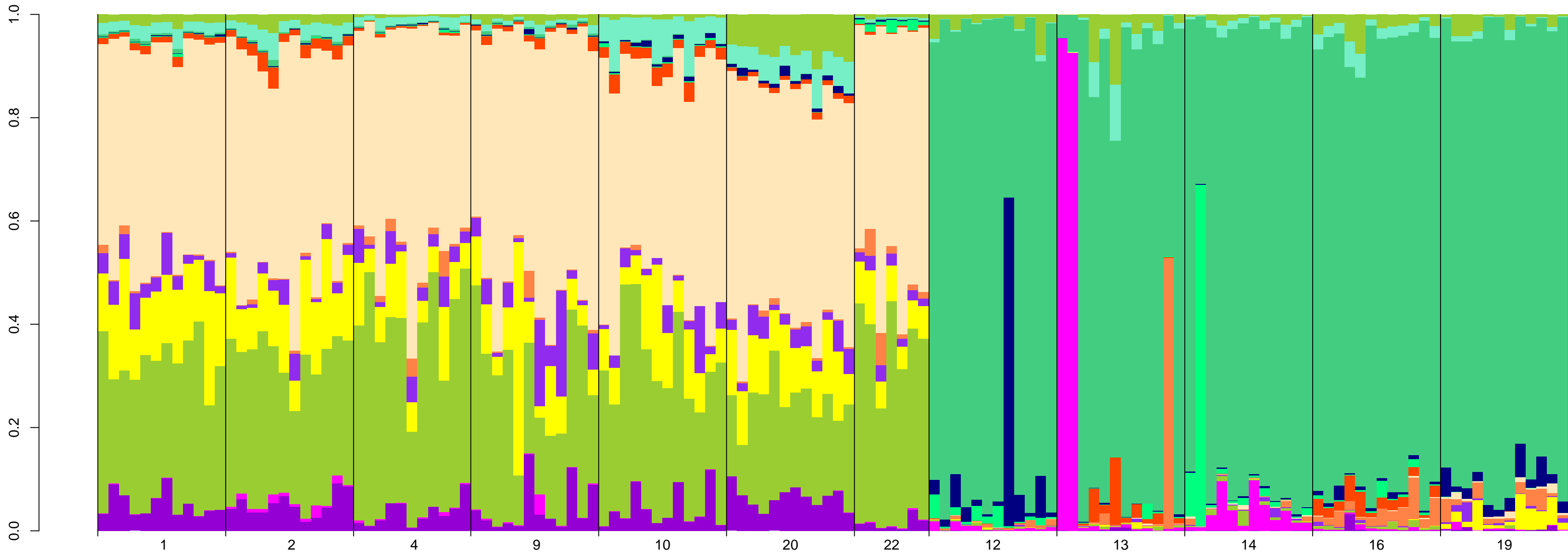

Supplement: Supplementary file 6 — Appendix S1 [file ECE3-10-4314-s006.zip › Appendix S1, STRUCTURE and PCA Plots, Dryad/STRUCTURE/C. austriacus & C. melapterus/job_T62.pdf]

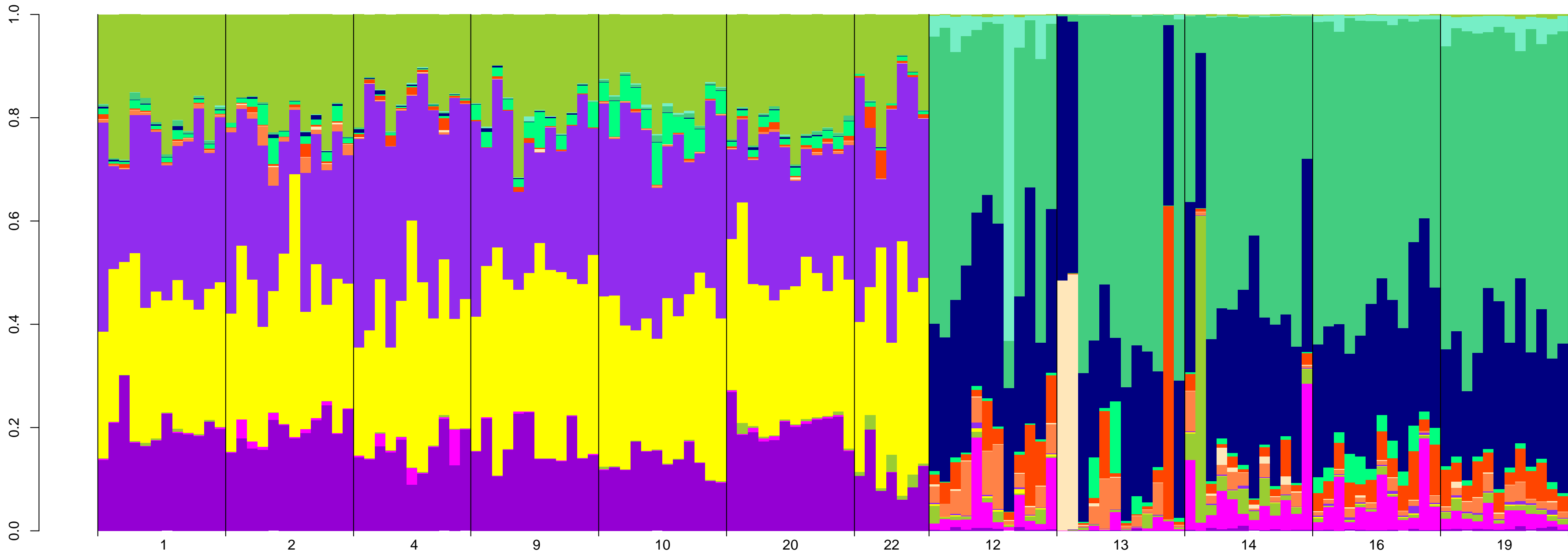

Supplement: Supplementary file 6 — Appendix S1 [file ECE3-10-4314-s006.zip › Appendix S1, STRUCTURE and PCA Plots, Dryad/STRUCTURE/C. austriacus & C. melapterus/job_T63.pdf]

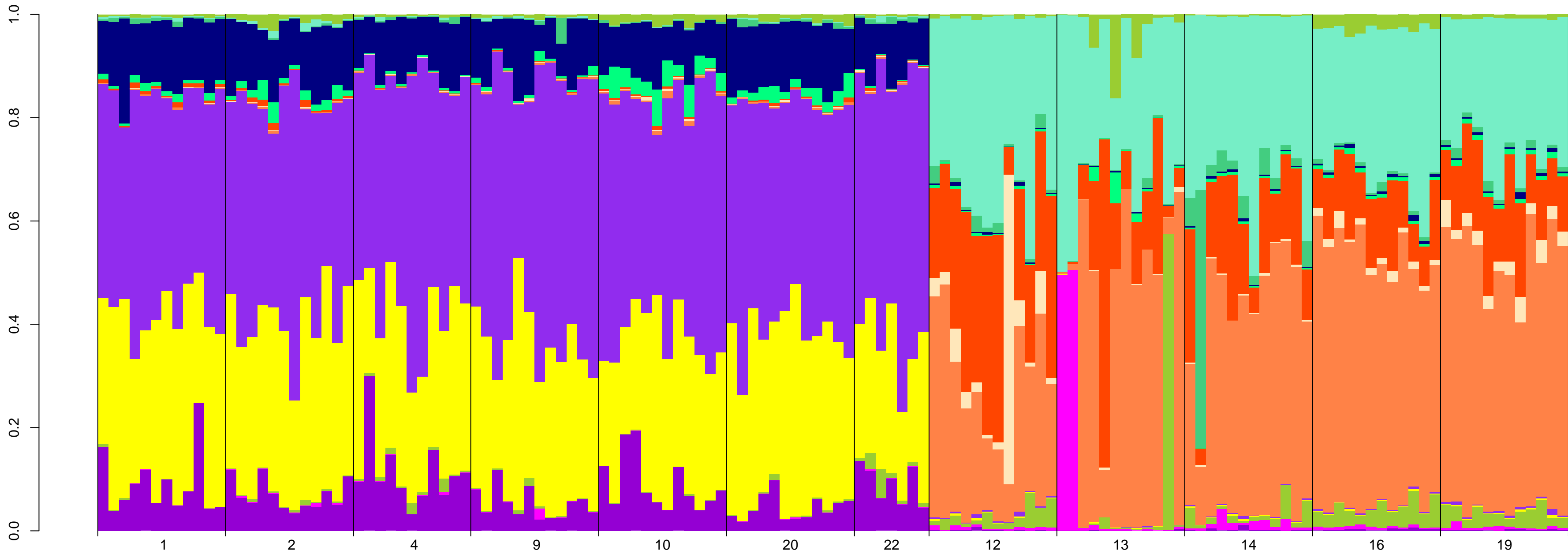

Supplement: Supplementary file 6 — Appendix S1 [file ECE3-10-4314-s006.zip › Appendix S1, STRUCTURE and PCA Plots, Dryad/STRUCTURE/C. austriacus & C. melapterus/job_T64.pdf]

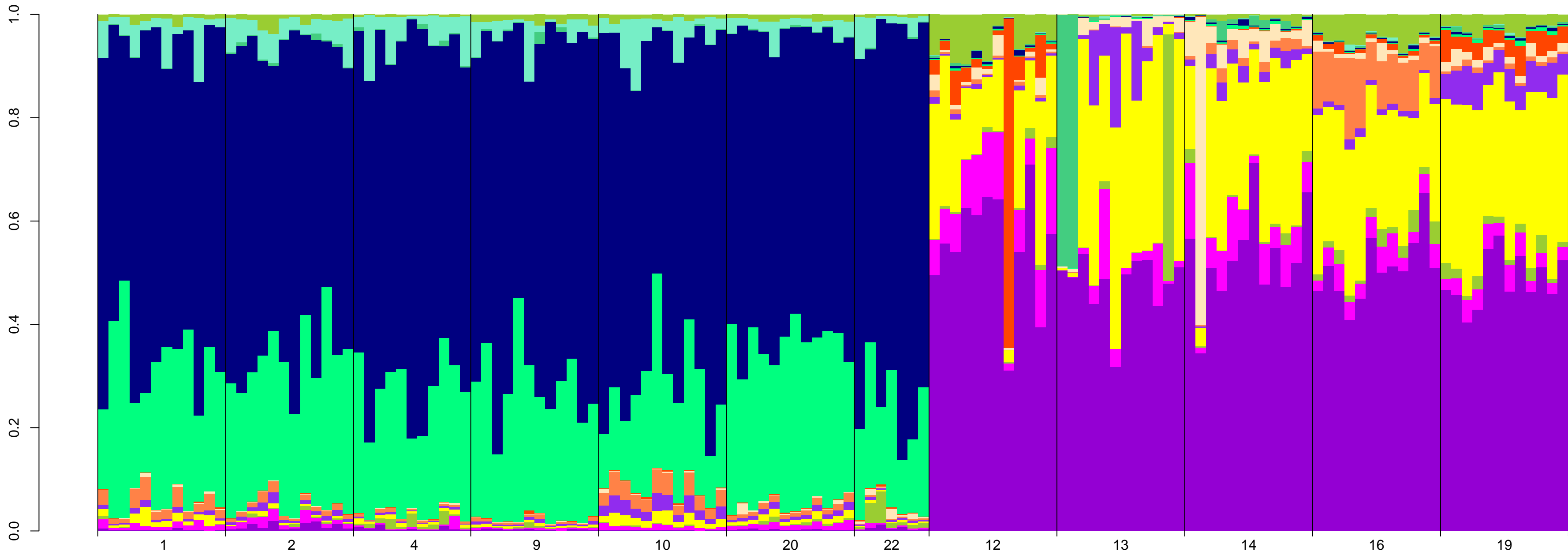

Supplement: Supplementary file 6 — Appendix S1 [file ECE3-10-4314-s006.zip › Appendix S1, STRUCTURE and PCA Plots, Dryad/STRUCTURE/C. austriacus & C. melapterus/job_T65.pdf]

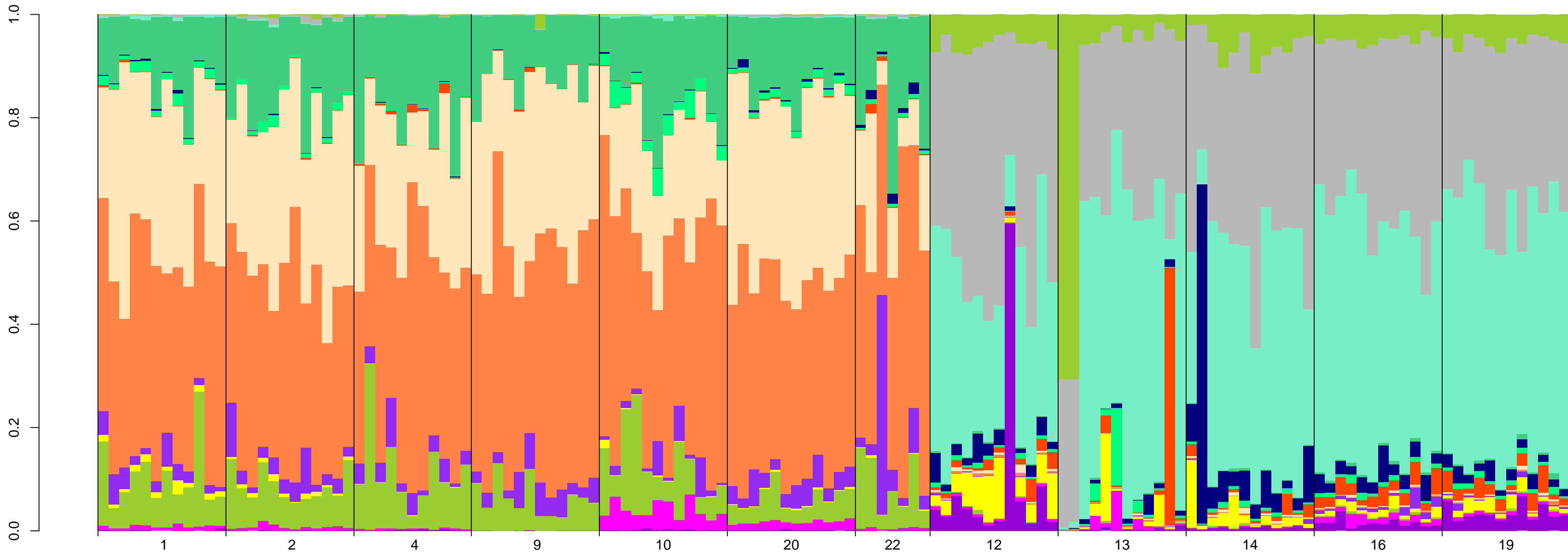

Supplement: Supplementary file 6 — Appendix S1 [file ECE3-10-4314-s006.zip › Appendix S1, STRUCTURE and PCA Plots, Dryad/STRUCTURE/C. austriacus & C. melapterus/job_T66.pdf]

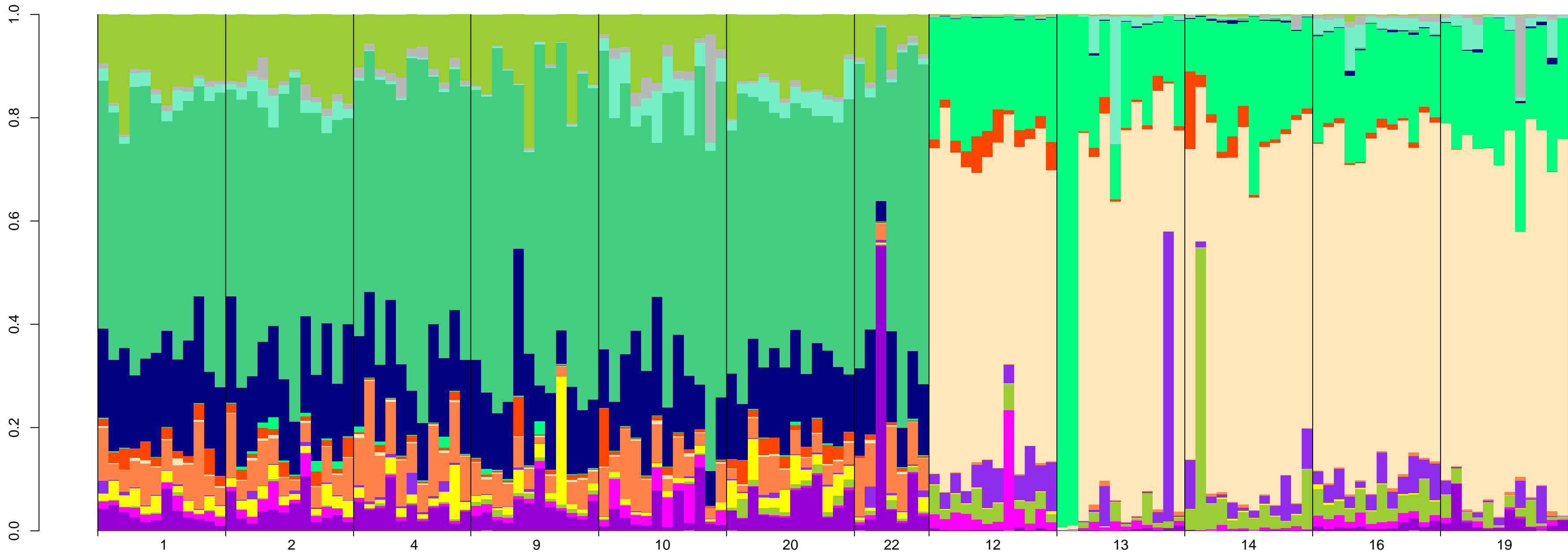

Supplement: Supplementary file 6 — Appendix S1 [file ECE3-10-4314-s006.zip › Appendix S1, STRUCTURE and PCA Plots, Dryad/STRUCTURE/C. austriacus & C. melapterus/job_T67.pdf]

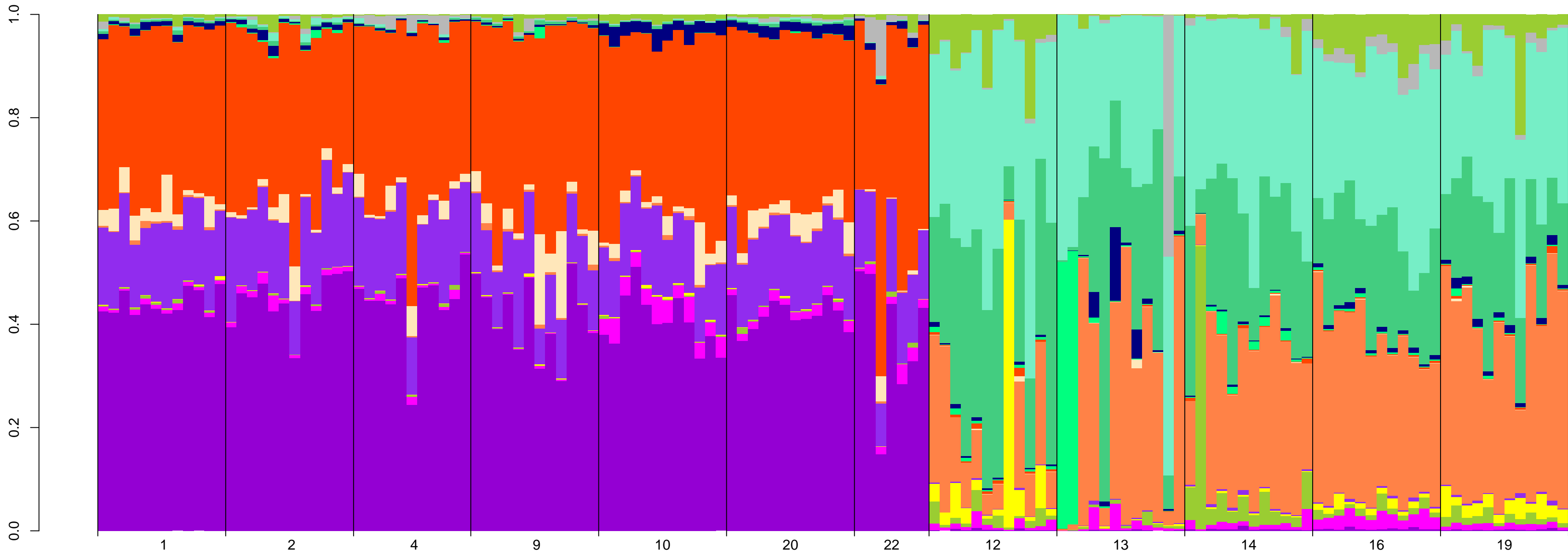

Supplement: Supplementary file 6 — Appendix S1 [file ECE3-10-4314-s006.zip › Appendix S1, STRUCTURE and PCA Plots, Dryad/STRUCTURE/C. austriacus & C. melapterus/job_T68.pdf]

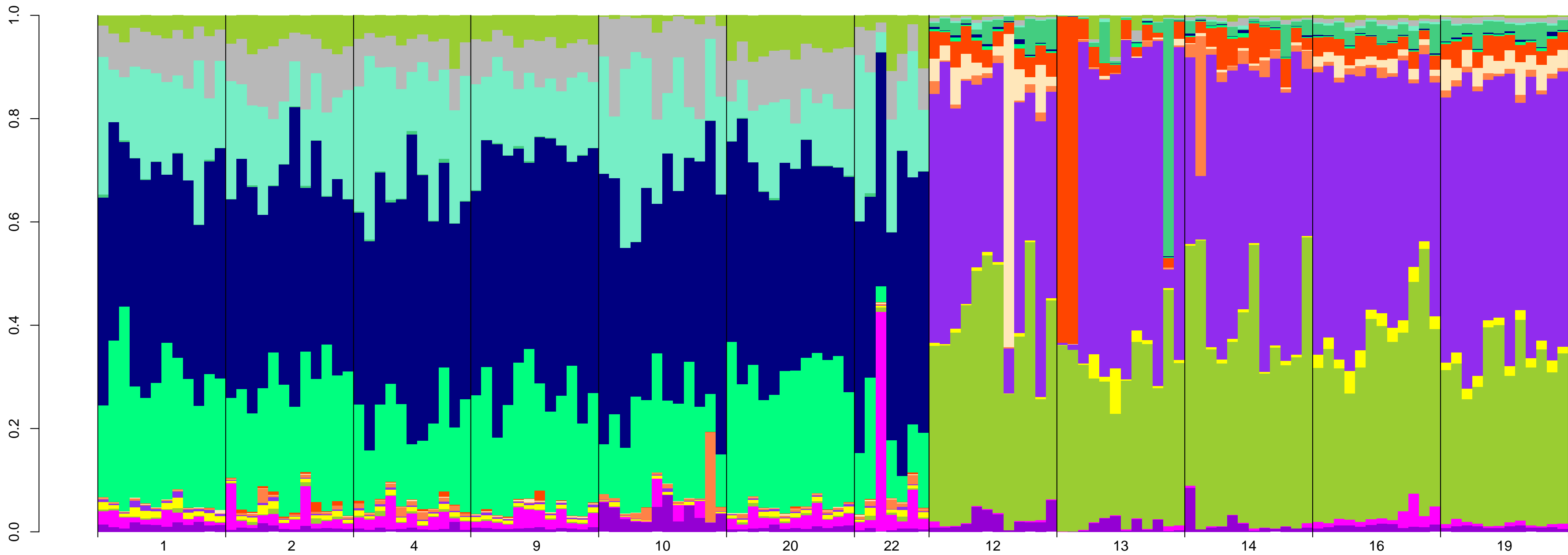

Supplement: Supplementary file 6 — Appendix S1 [file ECE3-10-4314-s006.zip › Appendix S1, STRUCTURE and PCA Plots, Dryad/STRUCTURE/C. austriacus & C. melapterus/job_T69.pdf]

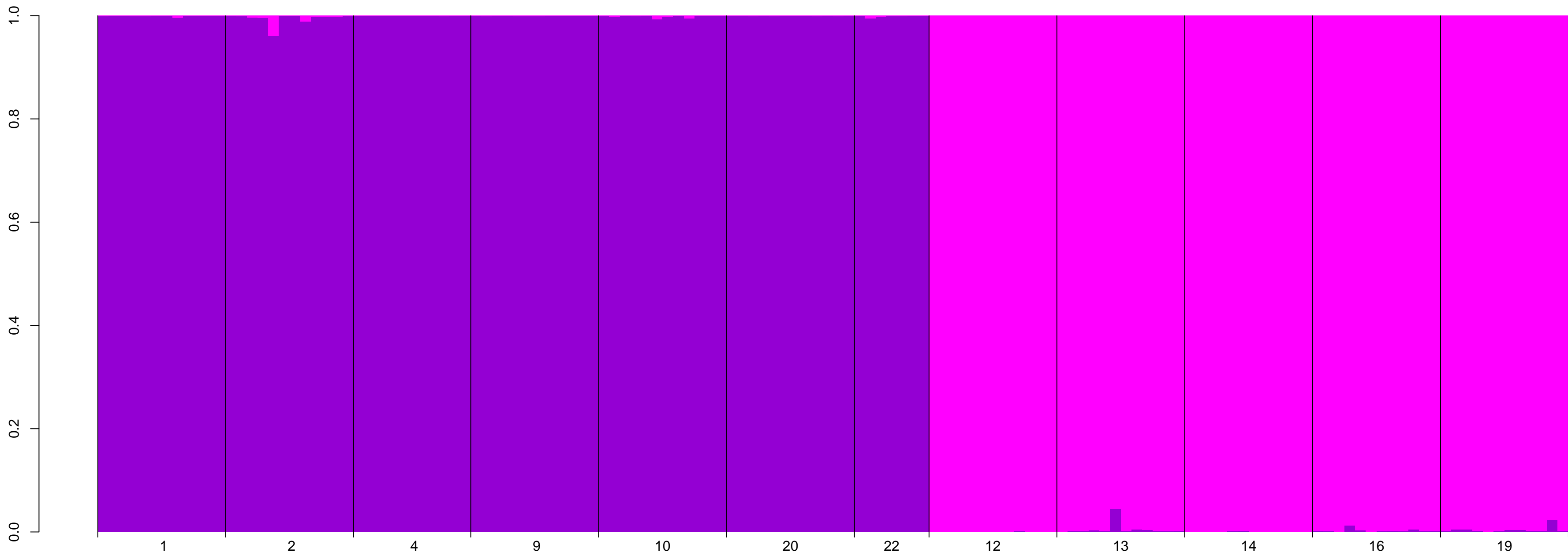

Supplement: Supplementary file 6 — Appendix S1 [file ECE3-10-4314-s006.zip › Appendix S1, STRUCTURE and PCA Plots, Dryad/STRUCTURE/C. austriacus & C. melapterus/job_T7.pdf]

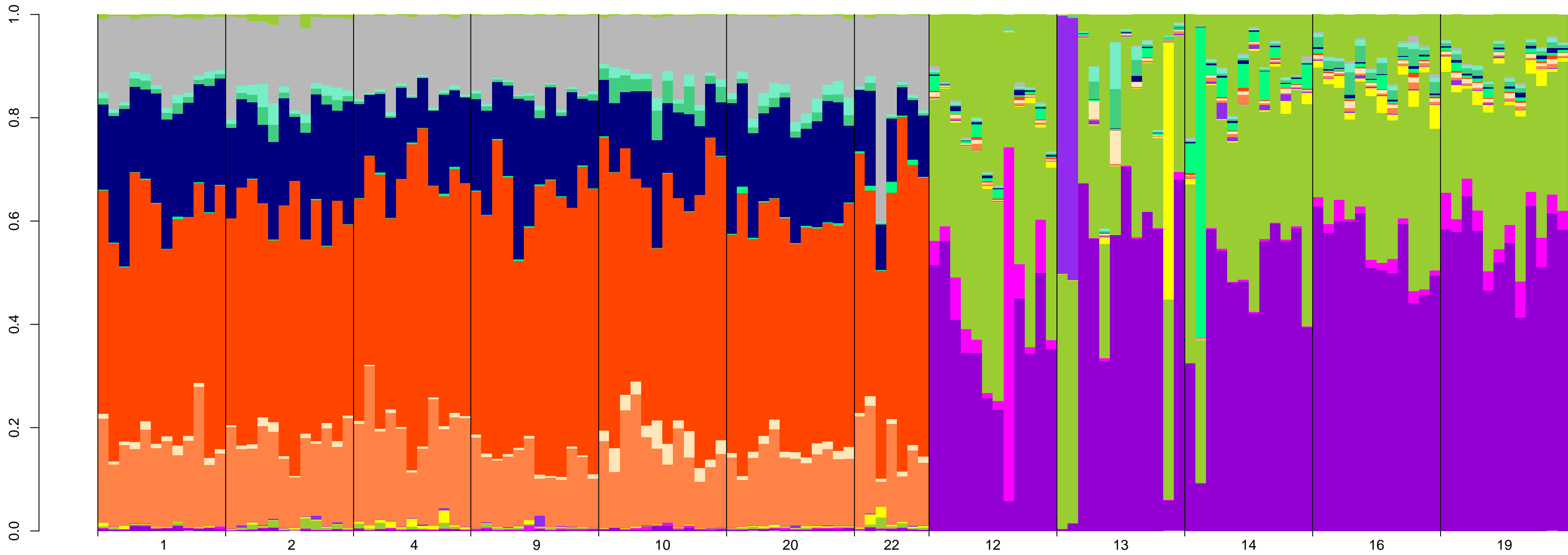

Supplement: Supplementary file 6 — Appendix S1 [file ECE3-10-4314-s006.zip › Appendix S1, STRUCTURE and PCA Plots, Dryad/STRUCTURE/C. austriacus & C. melapterus/job_T70.pdf]

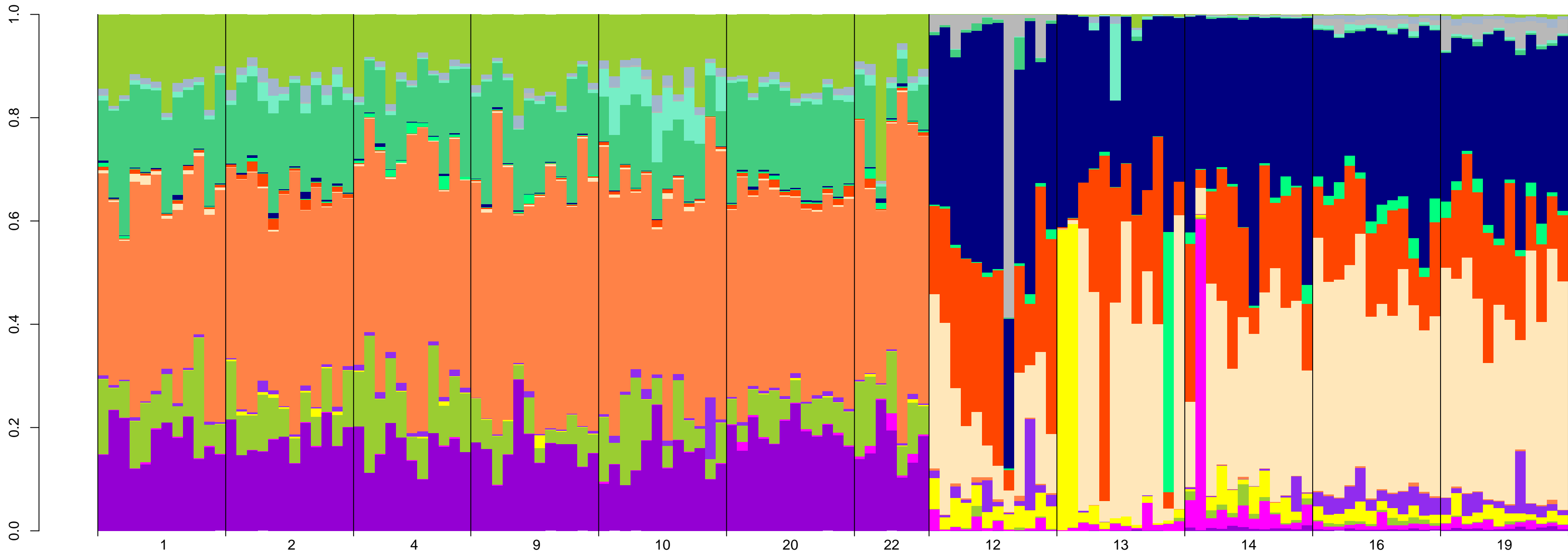

Supplement: Supplementary file 6 — Appendix S1 [file ECE3-10-4314-s006.zip › Appendix S1, STRUCTURE and PCA Plots, Dryad/STRUCTURE/C. austriacus & C. melapterus/job_T71.pdf]

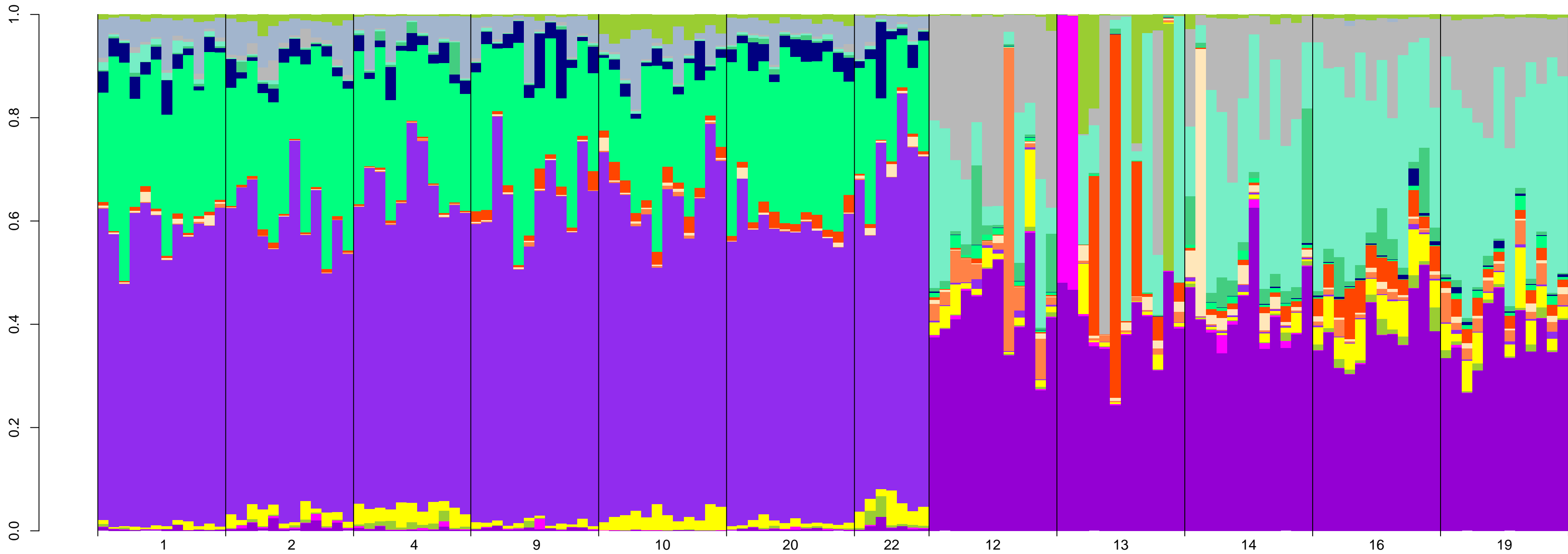

Supplement: Supplementary file 6 — Appendix S1 [file ECE3-10-4314-s006.zip › Appendix S1, STRUCTURE and PCA Plots, Dryad/STRUCTURE/C. austriacus & C. melapterus/job_T72.pdf]

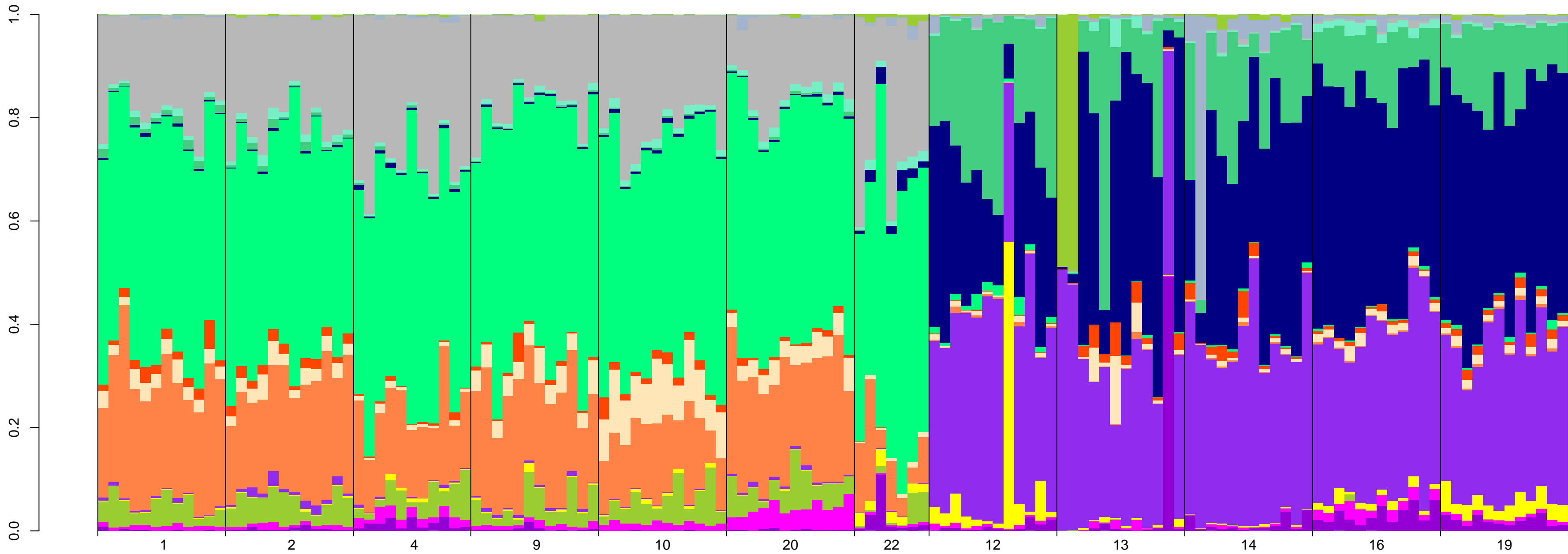

Supplement: Supplementary file 6 — Appendix S1 [file ECE3-10-4314-s006.zip › Appendix S1, STRUCTURE and PCA Plots, Dryad/STRUCTURE/C. austriacus & C. melapterus/job_T73.pdf]

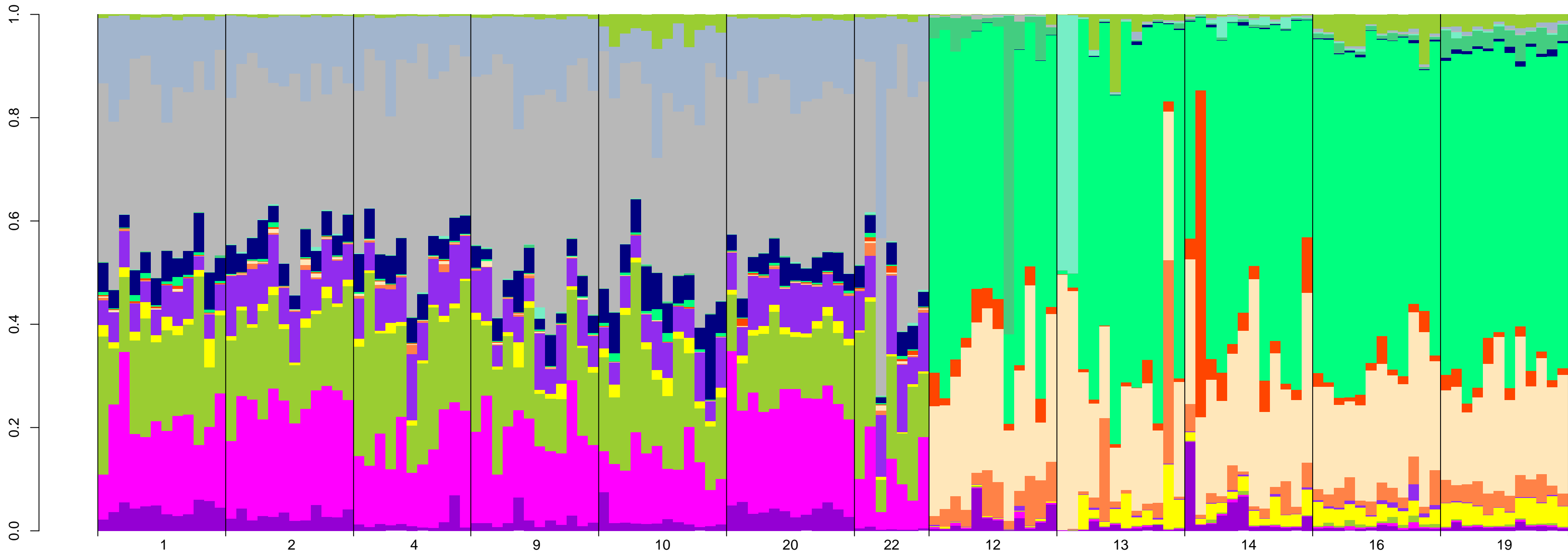

Supplement: Supplementary file 6 — Appendix S1 [file ECE3-10-4314-s006.zip › Appendix S1, STRUCTURE and PCA Plots, Dryad/STRUCTURE/C. austriacus & C. melapterus/job_T74.pdf]

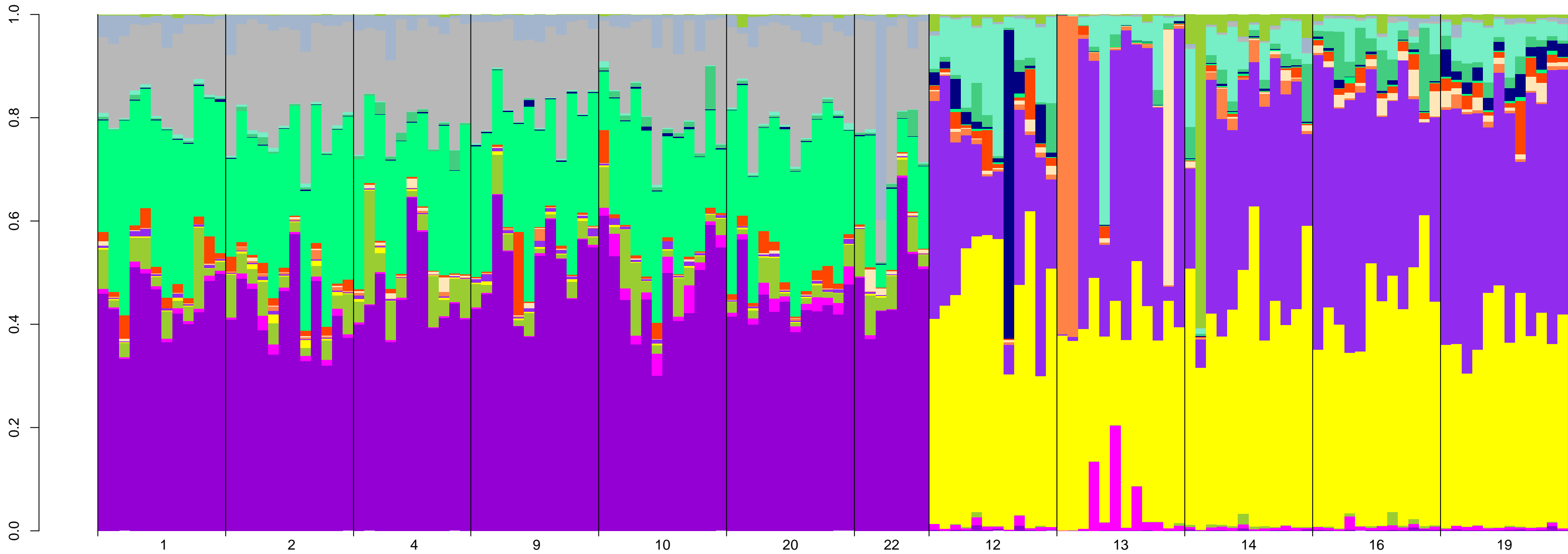

Supplement: Supplementary file 6 — Appendix S1 [file ECE3-10-4314-s006.zip › Appendix S1, STRUCTURE and PCA Plots, Dryad/STRUCTURE/C. austriacus & C. melapterus/job_T75.pdf]

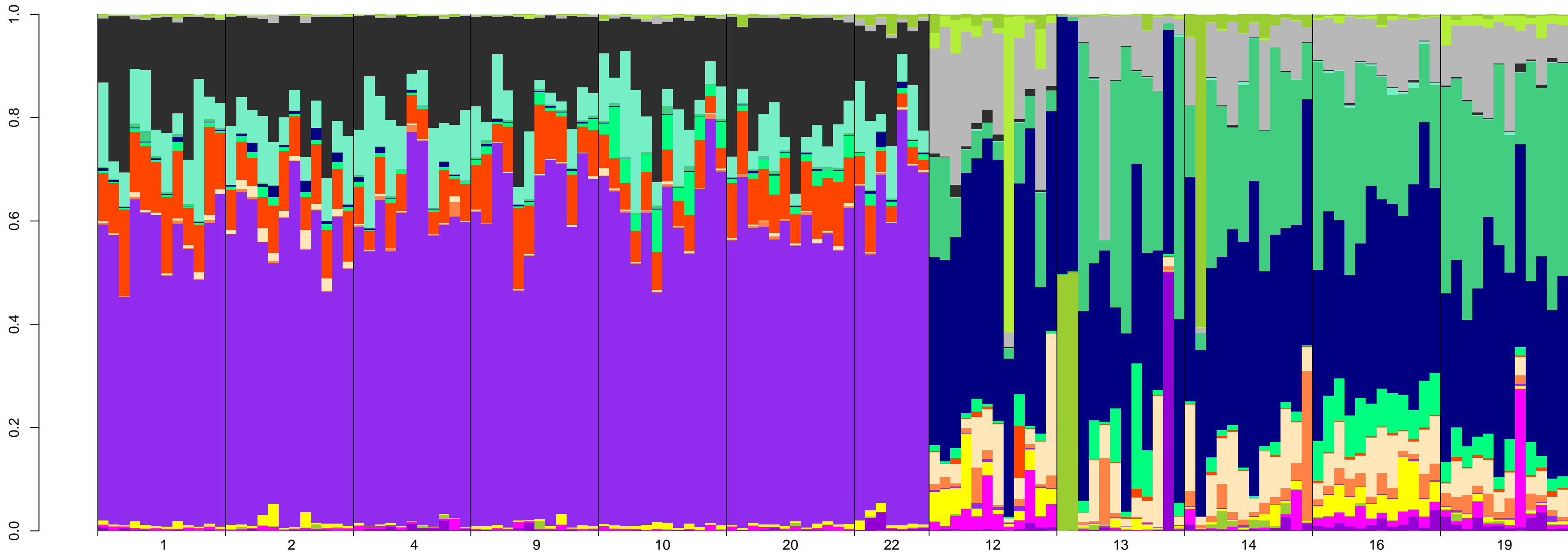

Supplement: Supplementary file 6 — Appendix S1 [file ECE3-10-4314-s006.zip › Appendix S1, STRUCTURE and PCA Plots, Dryad/STRUCTURE/C. austriacus & C. melapterus/job_T76.pdf]

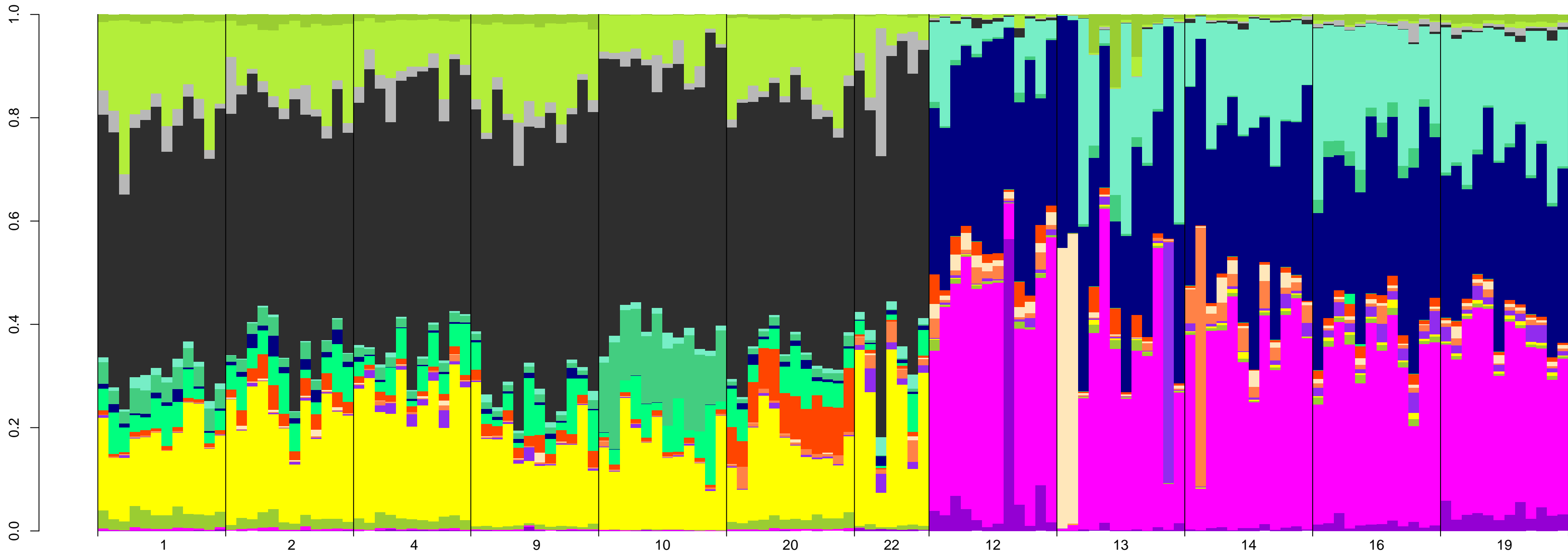

Supplement: Supplementary file 6 — Appendix S1 [file ECE3-10-4314-s006.zip › Appendix S1, STRUCTURE and PCA Plots, Dryad/STRUCTURE/C. austriacus & C. melapterus/job_T77.pdf]

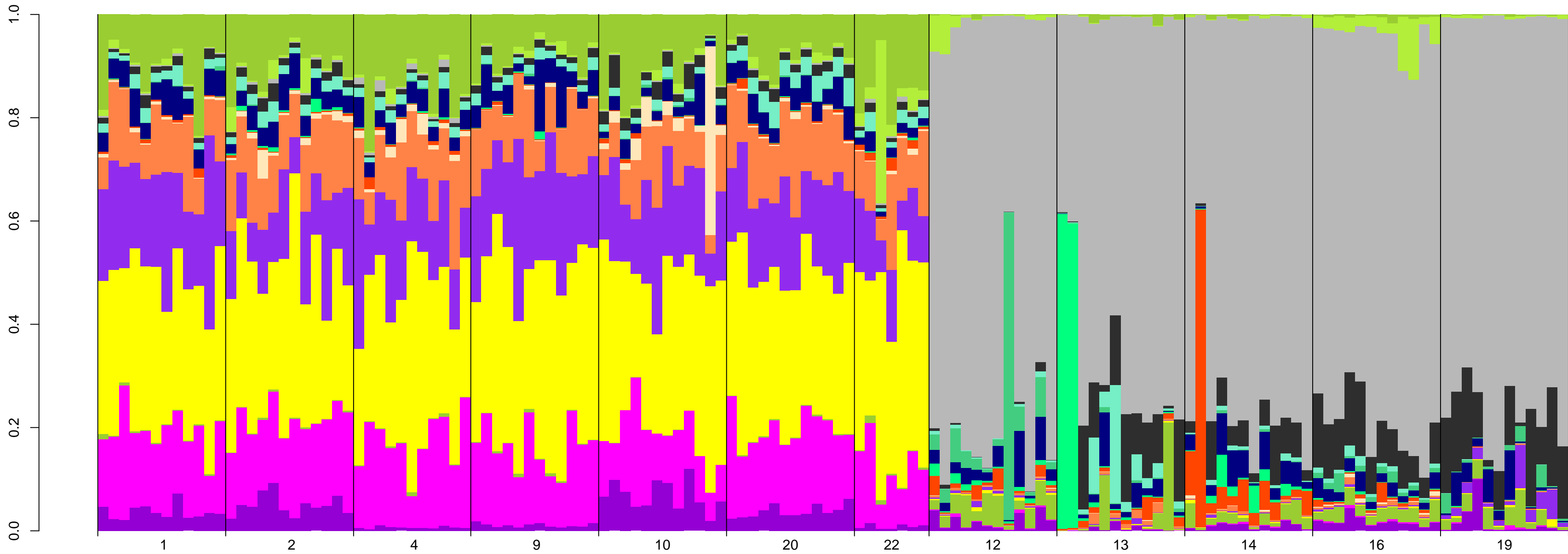

Supplement: Supplementary file 6 — Appendix S1 [file ECE3-10-4314-s006.zip › Appendix S1, STRUCTURE and PCA Plots, Dryad/STRUCTURE/C. austriacus & C. melapterus/job_T78.pdf]

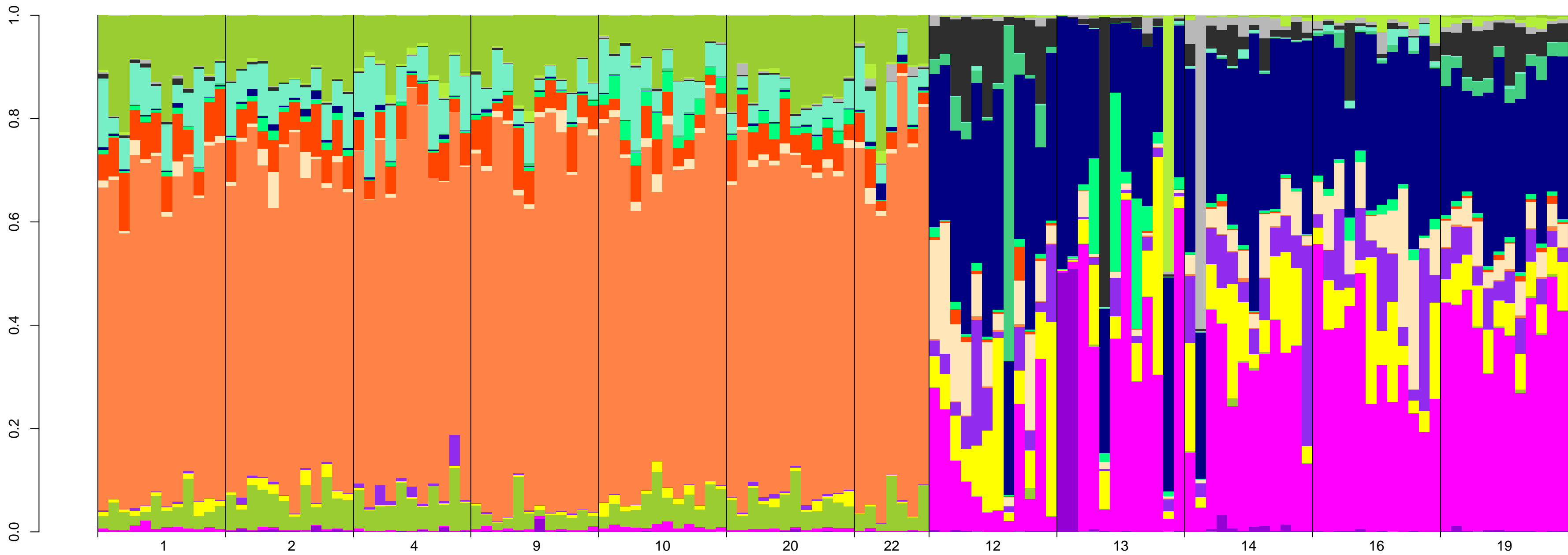

Supplement: Supplementary file 6 — Appendix S1 [file ECE3-10-4314-s006.zip › Appendix S1, STRUCTURE and PCA Plots, Dryad/STRUCTURE/C. austriacus & C. melapterus/job_T79.pdf]

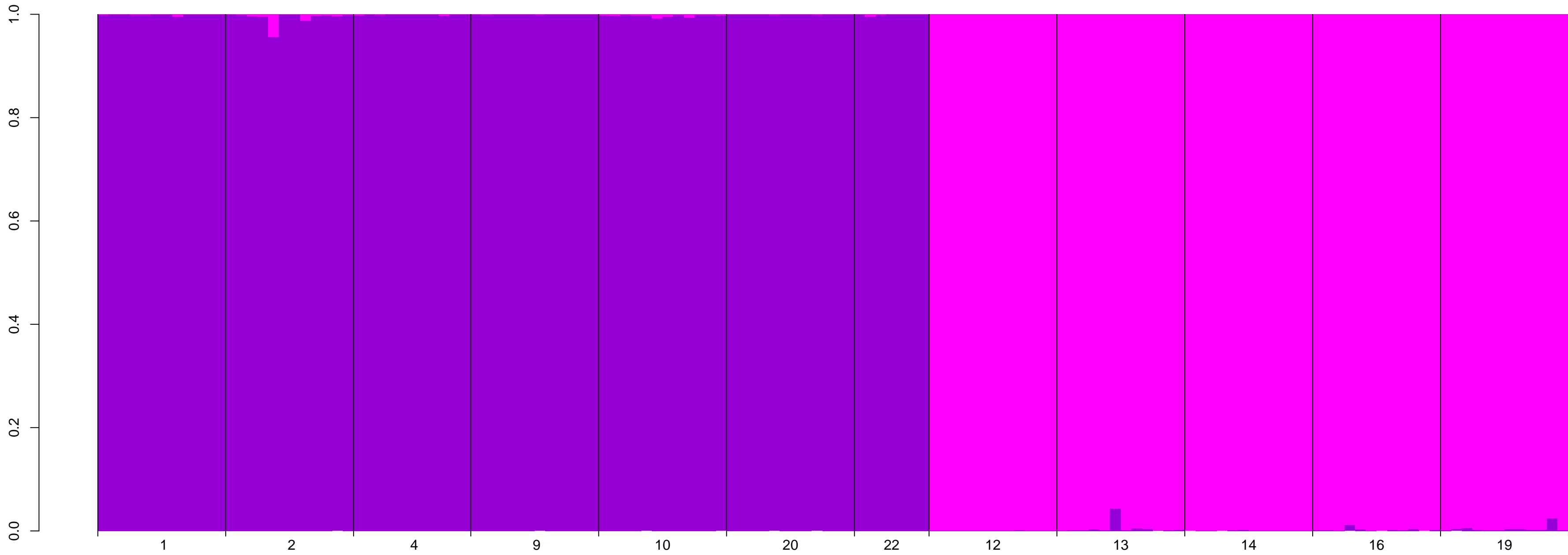

Supplement: Supplementary file 6 — Appendix S1 [file ECE3-10-4314-s006.zip › Appendix S1, STRUCTURE and PCA Plots, Dryad/STRUCTURE/C. austriacus & C. melapterus/job_T8.pdf]

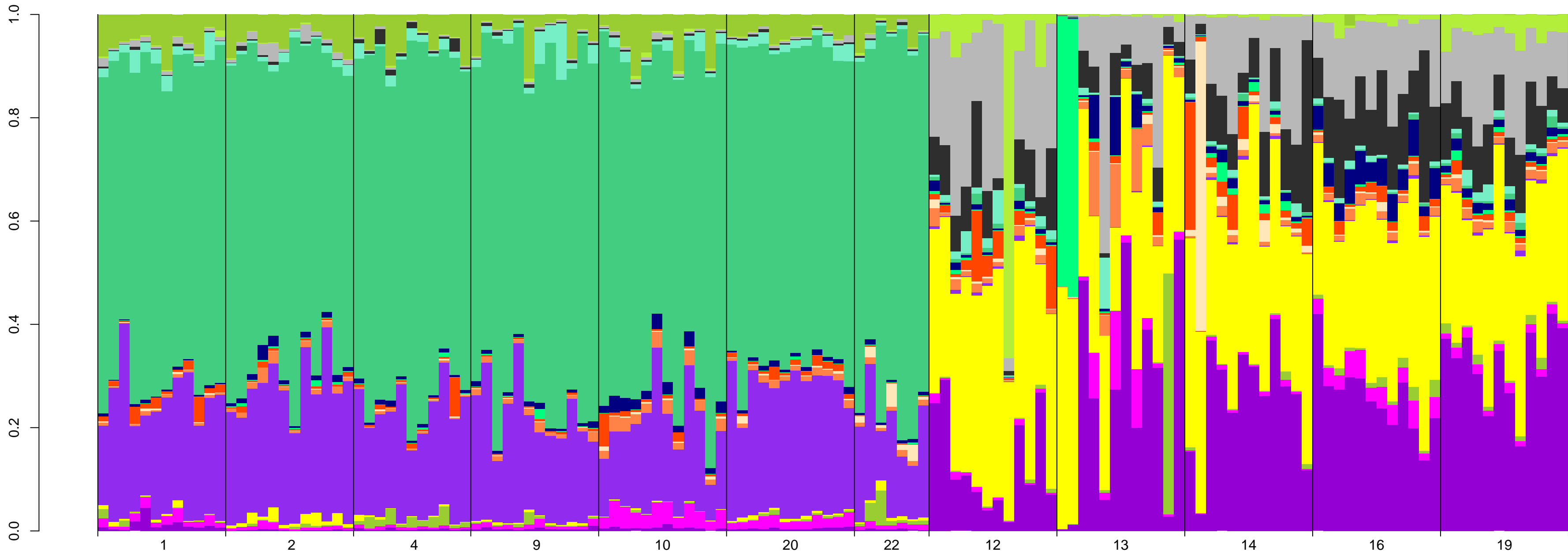

Supplement: Supplementary file 6 — Appendix S1 [file ECE3-10-4314-s006.zip › Appendix S1, STRUCTURE and PCA Plots, Dryad/STRUCTURE/C. austriacus & C. melapterus/job_T80.pdf]

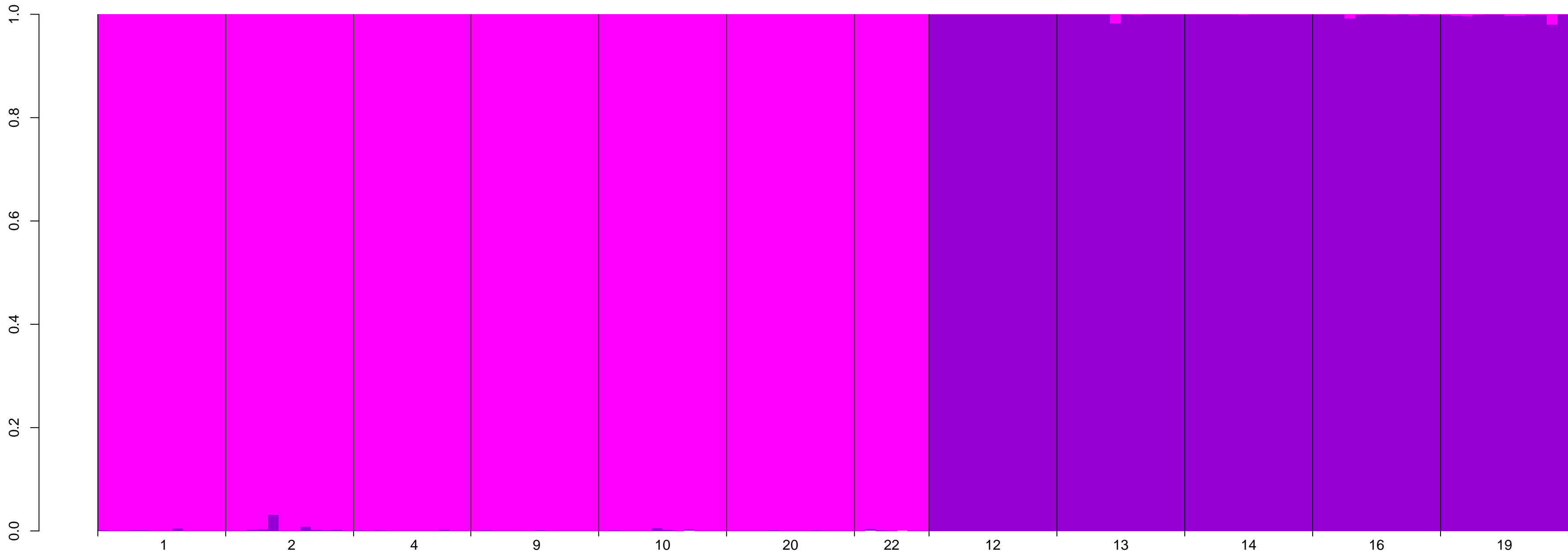

Supplement: Supplementary file 6 — Appendix S1 [file ECE3-10-4314-s006.zip › Appendix S1, STRUCTURE and PCA Plots, Dryad/STRUCTURE/C. austriacus & C. melapterus/job_T9.pdf]
